# Supplementary material for: Direct in situ protein tagging in Chlamydomonas reinhardtii utilizing TIM, a method for CRISPR/Cas9-based targeted insertional mutagenesis
Source: PLoS One. 2022 Dec 9;17(12):e0278972. doi: 10.1371/journal.pone.0278972 (PMC9733891; doi:10.1371/journal.pone.0278972)
Supplement: S1 Appendix — (DOCX) [file pone.0278972.s001.docx]

**S1 Appendix: Sequences of PCR products for 1) initial screening of LF5-HA strains (related to Fig 5 of main text) and 2) to check integrity of 3’ UTR of LF5-HA strains (related to Fig 7 of main text)**

Primer LF5-37: CACGAGTATCGCGTCGTCTG

Primer LF5-28: CAGTCCAGTCCTTTTGTTGTCG

Primer LF5-30: GGCTCGAACTCCTAGTCAACG

Yellow highlight and underline: PAM or mutated sequence

Grey highlight: HA tag

Double underline: last 108 nucleotides from the donor DNA

Underline: sequence matching wild-type *LF5* gene.

Bright green highlight: sequence matching paromomycin-resistance cassette

**Part I: Sequences of PCR screening products (related to Figure 5 of main text)**

Wild-type sequence (455 bp):

CACGAGTATCGCGTCGTCTGGGCAGCCGGTGCTGTACCAGACCAATGCGGCTGCGGGCGCCAGTAAGCTCAGTCGCGCGCCCAGCCGCGGCGACCCCTGGCAACAGTCGGGCGGTGGGCAGCAAGGGCGCGGCGCGATGCCGCCGCTGCCGCCCGGCGGTGGGCCGCGCATGAGCGGGCACTGGGACGACGACGGCGGCAACCCGGAGCGGCCGTACTCGCGGGGGATGCTGGGCGGCGGCGGCGGGCCCATGCAGCCGGGCTTTGGGCAGAACCAGATGTGGCCGCAGCTCAACGTGCAGCAGCAGCAACAGCAGCAGCGGAGGGGCAATTACTAGTAGCACAGTGACACTTACTGTGACGGCTTGCTTGCTAGAGGGGCAGCAGCTTCCCTACGCAAGCCCGGTTGTGTGCAGCGGGGCTGACAATGTCGGCGACAACAAAAGGACTGGACTG

Sequence expected for correct insertion of HA tag (563 bp):

CACGAGTATCGCGTCGTCTGGGCAGCCGGTGCTGTACCAGACCAATGCCGCTGCGGGCGCCAGTAAGCTCAGTCGCGCGCCCAGCCGCGGCGACCCCTGGCAACAGTCGGGCGGTGGGCAGCAAGGGCGCGGCGCGATGCCGCCGCTGCCGCCCGGCGGTGGGCCGCGCATGAGCGGGCACTGGGACGACGACGGCGGCAACCCGGAGCGGCCGTACTCGCGGGGGATGCTGGGCGGCGGCGGCGGGCCCATGCAGCCGGGCTTTGGGCAGAACCAGATGTGGCCGCAGCTCAACGTGCAGCAGCAGCAACAGCAGCAGCGGAGGGGCAATTACGGCCTGTCGCGATACCCCTACGACGTGCCCGACTACGCCTACCCCTACGACGTGCCCGACTACGCCGATCGATCCGGACCGTACCCCTACGACGTGCCCGACTACGCCGCTTAGCACAGTGACACTTACTGTGACGGCTTGCTTGCTAGAGGGGCAGCAGCTTCCCTACGCAAGCCCGGTTGTGTGCAGCGGGGCTGACAATGTCGGCGACAACAAAAGGACTGGACTG

Sequence of PCR product marked “S” in Figure 5 (563 bp):

CACGAGTATCGCGTCGTCTGGGCAGCCGGTGCTGTACCAGACCATGGGGATGTGACTATGTATTCGTGTGTTGGCCAACGGGTCAACCCGAACAGATTGATACCCGCCTTGGCATTTCCTGTCAGAATGTAACGTCAGTTGATGGTACCAGCATGCGGCTGCGGGCGCCAGTAAGCTCAGTCGCGCGCCCAGCCGCGGCGACCCCTGGCAACAGTCGGGCGGTGGGCAGCAAGGGCGCGGCGCGATGCCGCCGCTGCCGCCCGGCGGTGGGCCGCGCATGAGCGGGCACTGGGACGACGACGGCGGCAACCCGGAGCGGCCGTACTCGCGGGGGATGCTGGGCGGCGGCGGCGGGCCCATGCAGCCGGGCTTTGGGCAGAACCAGATGTGGCCGCAGCTCAACGTGCAGCAGCAGCAACAGCAGCAGCGGAGGGGCAATTACTAGTAGCACAGTGACACTTACTGTGACGGCTTGCTTGCTAGAGGGGCAGCAGCTTCCCTACGCAAGCCCGGTTGTGTGCAGCGGGGCTGACAATGTCGGCGACAACAAAAGGACTGGACTG

Sequence of PCR product marked “L1” in Figure 5 (1303 bp):

CACGAGTATCGCGTCGTCTGGGCAGCCGGTGCTGTACCAGACCGGGAGGGTGGTTGCAAACATGGTCGAGATTCGAAGCATGGACGATGCGTTGCGTGCACTGCGGGGTCGGTATCCCGGTTGTGAGTGGGTTGTTGTGGAGGATGGGGCCTCGGGGGCTGGTGTTTATCGGCTTCGGGGTGGTGGGCGGGAGTTGTTTGTCAAGGTGGCAGCTCTGGGGGCCGGGGTGGGCTTGTTGGGTGAGGCTGAGCGGCTGGTGTGGTTGGCGGAGGTGGGGATTCCCGTACCTCGTGTTGTGGAGGGTGGTGGGGACGAGAGGGTCGCCTGGTTGGTCACCGAAGCGGTTCCGGGGCGTCCGGCCAGTGCGCGGTGGCCGCGGGAGCAGCGGCTGGACGTGGCGGTGGCGCTCGCGGGGCTCGCTCGTTCGCTGCACGCGCTGGACTGGGAGCGGTGTCCGTTCGATCGCAGTCTCGCGGTGACGGTGCCGCAGGCGGCCCGTGCTGTCGCTGAAGGGAGCGTCGACTTGGAGGATCTGGACGAGGAGCGGAAGGGGTGGTCGGGGGAGCGGCTTCTCGCCGAGCTGGAGCGGACTCGGCCTGCGGACGAGGATCTGGCGGTTTGCCACGGTGACCTGTGCCCGGACAACGTGCTGCTCGACCCTCGTACCTGCGAGGTGACCGGGCTGATCGACGTGGGGCGGGTCGGCCGTGCGGACCGGCACTCCGATCTCGCGCTGGTGCTGCGCGAGCTGGCCCACGAGGAGGACCCGTGGTTCGGGCCGGAGTGTTCCGCGGCGTTCCTGCGGGAGTACGGGCGCGGGTGGGATGGGGCGGTATCGGAGGAAAAGCTGGCGTTTTACCGGCTGTTGGACGAGTTCTTCTGAGGGACCTACTTGCGGCTGCGGGCGCCAGTAAGCTCAGTCGCGCGCCCAGCCGCGGCGACCCCTGGCAACAGTCGGGCGGTGGGCAGCAAGGGCGCGGCGCGATGCCGCCGCTGCCGCCCGGCGGTGGGCCGCGCATGAGCGGGCACTGGGACGACGACGGCGGCAACCCGGAGCGGCCGTACTCGCGGGGGATGCTGGGCGGCGGCGGCGGGCCCATGCAGCCGGGCTTTGGGCAGAACCAGATGTGGCCGCAGCTCAACGTGCAGCAGCAGCAACAGCAGCAGCGGAgGGGCAATTACTAGTAGCACAGTGACACTTACTGTGACGGCTTGCTTGCTAGAGGGGCAGCAGCTTCCCTACGCAAGCCCGGTTGTGTGCAGCGGGGCTGACAATGTCGGCGACAACAAAAGGACTGGACTG

Sequence of PCR product marked “L2” in Figure 5 (1499 bp):

CACGAGTATCGCGTCGTCTGGGCAGCCGGTGCTGTCTTCCGGCGCCTCTTCCTCTTCGTTTCAGTCACAACCCGCAAACATGGTCGAGATTCGAAGCATGGACGATGCGTTGCGTGCACTGCGGGGTCGGTATCCCGGTTGTGAGTGGGTTGTTGTGGAGGATGGGGCCTCGGGGGCTGGTGTTTATCGGCTTCGGGGTGGTGGGCGGGAGTTGTTTGTCAAGGTGGCAGCTCTGGGGGCCGGGGTGGGCTTGTTGGGTGAGGCTGAGCGGCTGGTGTGGTTGGCGGAGGTGGGGATTCCCGTACCTCGTGTTGTGGAGGGTGGTGGGGACGAGAGGGTCGCCTGGTTGGTCACCGAAGCGGTTCCGGGGCGTCCGGCCAGTGCGCGGTGGCCGCGGGAGCAGCGGCTGGACGTGGCGGTGGCGCTCGCGGGGCTCGCTCGTTCGCTGCACGCGCTGGACTGGGAGCGGTGTCCGTTCGATCGCAGTCTCGCGGTGACGGTGCCGCAGGCGGCCCGTGCTGTCGCTGAAGGGAGCGTCGACTTGGAGGATCTGGACGAGGAGCGGAAGGGGTGGTCGGGGGAGCGGCTTCTCGCCGAGCTGGAGCGGACTCGGCCTGCGGACGAGGATCTGGCGGTTTGCCACGGTGACCTGTGCCCGGACAACGTGCTGCTCGACCCTCGTACCTGCGAGGTGACCGGGCTGATCGACGTGGGGCGGGTCGGCCGTGCGGACCGGCACTCCGATCTCGCGCTGGTGCTGCGCGAGCTGGCCCACGAGGAGGACCCGTGGTTCGGGCCGGAGTGTTCCGCGGCGTTCCTGCGGGAGTACGGGCGCGGGTGGGATGGGGCGGTATCGGAGGAAAAGCTGGCGTTTTACCGGCTGTTGGACGAGTTCTTCTGAGGGACCTGATGGTGTTGGTGGCTGGGTAGGGTTGCGTCGCGTGGGTGACGGCACAGTGTGGACGTTGGGATCCGGCAAGACTGGCCCCGCTTGGCAACGCAACAGTGAGCCCCTCCCTAGTGTGTTTGGGGATGTGACTATGTATTCGTGTGTTGGCCAACGGGTCAACCCGAACAGATTGATACCCCGGCGGCTGCGGGCGCCAGTAAGCTCAGTCGCGCGCCCAGCCGCGGCGACCCCTGGCAACAGTCGGGCGGTGGGCAGCAAGGGCGCGGCGCGATGCCGCCGCTGCCGCCCGGCGGTGGGCCGCGCATGAGCGGGCACTGGGACGACGACGGCGGCAACCCGGAGCGGCCGTACTCGCGGGGGATGCTGGGCGGCGGCGGCGGGCCCATGCAGCCGGGCTTTGGGCAGAACCAGATGTGGCCGCAGCTCAACGTGCAGCAGCAGCAACAGCAGCAGCGGagGGGCAATTACTAGTAGCACAGTGACACTTACTGTGACGGCTTGCTTGCTAGAGGGGCAGCAGCTTCCCTACGCAAGCCCGGTTGTGTGCAGCGGGGCTGACAATGTCGGCGACAACAAAAGGACTGGACTG

Sequence of PCR product marked “L3” in Figure 5 (1390 bp):

CACGAGTATCGCGTCGTCTGGGCAGCCGGTGCCCGGACAACGTGCTGCTCGACCCTCGTACCTGCGAGGTGACCGGGCTGATCGACGTGGGGCGGGTCGGCCGtGCGGACCGGCACTCCGATCTCGCGCTGGTGCTGCGTTGCGTGCACTGCGGGGTCGGTATCCCGGTTGTGAGTGGGTTGTTGTGGAGGATGGGGCCTCGGGGGCTGGTGTTTATCGGCTTCGGGGTGGTGGGCGGGAGTTGTTTGTCAAGGTGGCAGCTCTGGGGGCCGGGGTGGGCTTGTTGGGTGAGGCTGAGCGGCTGGTGTGGTTGGCGGAGGTGGGGATTCCCGTACCTCGTGTTGTGGAGGGTGGTGGGGACGAGAGGGTCGCCTGGTTGGTCACCGAAGCGGTTCCGGGGCGTCCGGCCAGTGCGCGGTGGCCGCGGGAGCAGCGGCTGGACGTGGCGGTGGCGCTCGCGGGGCTCGCTCGTTCGCTGCACGCGCTGGACTGGGAGCGGTGTCCGTTCGATCGCAGTCTCGCGGTGACGGTGCCGCAGGCGGCCCGTGCTGTCGCTGAAGGGAGCGTCGACTTGGAGGATCTGGACGAGGAGCGGAAGGGGTGGTCGGGGGAGCGGCTTCTCGCCGAGCTGGAGCGGACTCGGCCTGCGGACGAGGATCTGGCGGTTTGCCACGGTGACCTGTGCCCGGACAACGTGCTGCTCGACCCTCGTACCTGCGAGGTGACCGGGCTGATCGACGTGGGGCGGGGTCGGCCGTGCGGACCGGCACTCCGATCTCGCGCTGGTGCTGCGCGAGCTGGCCCACGAGGAGGACCCGTGGTTCGGGCCGGAGTGTTCCGCGGCGTTCCTGCGGGAGTACGGGCGCGGGTGGGATGGGGCGGTATCGGAGGAAAAGCTGGCGTTTTACCGGCTGTTGGACGAGTTCTTCTGAGGGACCTGATGGTGTTGGTGGCTGGGTAGGGTTGCGTCGCGTGGGTGATGCGGCTGCGGGCGCCAGTAAGCTCAGTCGCGCGCCCAGCCGCGGCGACCCCTGGCAACAGTCGGGCGGTGGGCAGCAAGGGCGCGGCGCGATGCCGCCGCTGCCGCCCGGCGGTGGGCCGCGCATGAGCGGGCACTGGGACGACGACGGCGGCAACCCGGAGCGGCCGTACTCGCGGGGGATGCTGGGCGGCGGCGGCGGGCCCATGCAGCCGGGCTTTGGGCAGAACCAGATGTGGCCGCAGCTCAACGTGCAGCAGCAGCAACAGCAGCAGCGGAgGGGCAATTACTAGTAGCACAGTGACACTTACTGTGACGGCTTGCTTGCTAGAGGGGCAGCAGCTTCCCTACGCAAGCCCGGTTGTGTGCAGCGGGGCTGACAATGTCGGCGACAACAAAAGGACTGGACTG

Sequence of PCR product marked “L4” in Figure 5 (1808 bp):

CACGAGTATCGCGTCGTCTGGGCAGCCGGTGCTGTACCAGACCAATGCCGCTGCGGGCGCCAGTAAGCTCAGTCGCGCGCCCAGCCGCGGCGACCCCTGGCAACAGTCGGGCGGTGGGCAGCAAGGGCGCGGCGCGATGCCGCCGCTGCCGCCCGGCGGTGGGCCGCGCATGAGCGGGCACTGGGACGACGACGGCGGCAACCCGGAGCGGCCGTACTCGCGGGGGATGCTGGGCGGCGGCGGCGGGCCCATGCAGCCGGGCTTTGGGCAGAACCAGATGTGGCCGCAGCTCAACGTGCAGCAGCAGCAACAGCAGCAGCGGAGGGGCAATTACGGCCTGTCGCGATACCCCTACGACGTGCCCGACTACGCGAAGATTCGAAGCATGGACGATGCGTTGCGTGCACTGCGGGGTCGGTATCCCGGTTGTGAGTGGGTTGTTGTGGAGGATGGGGCCTCGGGGGCTGGTGTTTATCGGCTTCGGGGTGGTGGGCGGGAGTTGTTTGTCAAGGTGGCAGCTCTGGGGGCCGGGGTGGGCTTGTTGGGTGAGGCTGAGCGGCTGGTGTGGTTGGCGGAGGTGGGGATTCCCGTACCTCGTGTTGTGGAGGGTGGTGGGGACGAGAGGGTCGCCTGGTTGGTCACCGAAGCGGTTCCGGGGCGTCCGGCCAGTGCGCGGTGGCCGCGGGAGCAGCGGCTGGACGTGGCGGTGGCGCTCGCGGGGCTCGCTCGTTCGCTGCACGCGCTGGACTGGGAGCGGTGTCCGTTCGATCGCNGTCTCGCGGTGACGGTGCCGCAGGCGGCCCGTGCTGTCGCTGAAGGGAGCGTCGACTTGGAGGATCTGGACGAGGAGCGGAAGGGGTGGTCGGGGGAGCGGCTTCTCGCCGAGCTGGAGCGGACTCGGCCTGCGGACGAGGATCTGGCGGTTTGCCACGGTGACCTGTGCCCGGACAACGTGCTGCTCGACCCTCGTACCTGCGAGGTGACCGGGCTGATCGACGTGGGGCGGGTCGGCCGTGCGGACCGGCACTCCGATCTCGCGCTGGTGCTGCGCGAGCTGGCCCACGAGGAGGACCCGTGGTTCGGGCCGGAGTGTTCCGCGGCGTTCCTGCGGGAGTACGGGCGCGGGTGGGATGGGGCGGTATCGGAGGAAAAGCTGGCGTTTTACCGGCTGTTGGACGAGTTCTTCTGAGGGACCTGATGGTGTTGGTGGCTGGGTAGGGTTGCGTCGCGTGGGTGACAGCACAGTGTGGACGTTGGGATCCGGCAAGACTGGCCCCGCTTGGCAACGCAACAGTGAGCCCCTCCCTAGTGTGTTTGGGGATGTGACTATGTATTCGTGTGTTGGCCAACGGGTCAACCCGAACAGATTGATACCCGCCTTGGCATTTCCTGTCAGAATGCGGCTGCGGGCGCCAGTAAGCTCAGTCGCGCGCCCAGCCGCGGCGACCCCTGGCAACAGTCGGGCGGTGGGCAGCAAGGGCGCGGCGCGATGCCGCCGCTGCCGCCCGGCGGTGGGCCGCGCATGAGCGGGCACTGGGACGACGACGGCGGCAACCCGGAGCGGCCGTACTCGCGGGGGATGCTGGGCGGCGGCGGCGGGCCCATGCAGCCGGGCTTTGGGCAGAACCAGATGTGGCCGCAGCTCAACGTGCAGCAGCAGCAACAGCAGCAGCGGAGGGGCAATTACTAGTAGCACAGTGACACTTACTGTGACGGCTTGCTTGCTAGAGGGGCAGCAGCTTCCCTACGCAAGCCCGGTTGTGTGCAGCGGGGCTGACAATGTCGGCGACAACAAAAGGACTGGACTG

Sequence of PCR product marked “L5” in Figure 5 (1236 bp):

CACGAGTATCGCGTCGTCTGGGCAGCCGGTGCTGTACCAGACCATCGAGAttcGAAGCATGGACGATGCGTTGCGTGCACTGCGGGGTCGGTATCCCGGTTGTGAGTGGGTTGTTGTGGAGGATGGGGCCTCGGGGGCTGGTGTTTATCGGCTTCGGGGTGGTGGGCGGGAGTTGTTTGTCAAGGTGGCAGCTCTGGGGGCCGGGGTGGGCTTGTTGGGTGAGGCTGAGCGGCTGGTGTGGTTGGCGGAGGTGGGGATTCCCGTACCTCGTGTTGTGGAGGGTGGTGGGGACGAGAGGGTCGCCTGGTTGGTCACCGAAGCGGTTCCGGGGCGTCCGGCCAGTGCGCGGTGGCCGCGGGAGCAGCGGCTGGACGTGGCGGTGGCGCTCGCGGGGCTCGCTCGTTCGCTGCACGCGCTGGACTGGGAGCGGTGTCCGTTCGATCGCAGTCTCGCGGTGACGGTGCCGCAGGCGGCCCGTGCTGTCGCTGAAGGGAGCGTCGACTTGGAGGATCTGGACGAGGAGCGGAAGGGGTGGTCGGGGGAGCGGCTTCTCGCCGAGCTGGAGCGGACTCGGCCTGCGGACGAGGATCTGGCGGTTTGCCACGGTGACCTGTGCCCGGACAACGTGCTGCTCGACCCTCGTACCTGCGAGGTGACCGGGCTGATCGACGTGGGGCGGGTCGGCCGTGCGGACCGGCACTCCGATCTCGCGCTGGTGCTGCGCGAGCTGGCCCACGAGGAGGACCCGTGGTTCGGGCCGGAGTGTTCCGCGGCGTTCCTGCGGGAGTACGGGCGCGGGTGGGATGGGGCGGTATCGGAGGAAAAGCTGGCGTTTTACCGGCTGTTGGACGAGTTCTTCTGAGGGACCTGATGGTGTTGGTGGTCAGTCGGGCGGTGGGCAGCAAGGGCGCGGCGCGATGCCGCCGCTGCCGCCCGGCGGTGGGCCGCGCATGAGCGGGCACTGGGACGACGACGGCGGCAACCCGGAGCGGCCGTACTCGCGGGGGATGCTGGGCGGCGGCGGCGGGCCCATGCAGCCGGGCTTTGGGCAGAACCAGATGTGGCCGCAGCTCAACGTGCAGCAGCAGCAACAGCAGCAGCGGAgGGGCAATTACTAGTAGCACAGTGACACTTACTGTGACGGCTTGCTTGCTAGAGGGGCAGCAGCTTCCCTACGCAAGCCCGGTTGTGTGCAGCGGGGCTGACAATGTCGGCGACAACAAAAGGACTGGACTG

Sequence of PCR product marked “L6” in Figure 5 (1628 bp):

CACGAGTATCGCGTCGTCTGGGCAGCCGGTGCTGTACCAGAttCGAAGCATGGACGATGCGTTGCGTGCACTGCGGGGTCGGTATCCCGGTTGTGAGTGGGTTGTTgTGGAGGATGGGGCCTCGGGGGCTGGTGTTTATCGGCTTCggGGTGGTGGGCGGGAGTTGTTTGTCAAGGTGGCAGCTCTGGGGGCCGGGGTGGGCTTGTTGGGTGAGGCTGagCGgcTGGTGTGGTTGGCGGAGGTGGGGATTCCCGTACCTCGTGTTGTGGAGGGTGGTGGGGACGAGAGGGTCGCCTGGTTGGTCACCGAAGCGGTTCCGGGGCGTCCGGCCAGTGCGCGGTGGCCGCGGGAGCAGCGGCTGGACGTGGCGGTGGCGCTCGCGGGGCTCGCTCGTTCGCTGCACGCGCTGGACTGGGAGCGGTGTCCGTTCGATCGCAGTCTCGCGGTGACGGTGCCGCAGGCGGCCCGTGCTGTCGCTGAAGGGAGCGTCGACTTGGAGGATCTGGACGAGGAGCGGAAGGGGTGGTCGGGGGAGCGGCTTCTCGCCGAGCTGGAGCGGACTCGGCCTGTGGACGAGGATCTGGCGGTTTGCCACGGTGACCTGTGCCCGGACAACGTGCTGCTCGACCCTCGTACCTGCGAGGTGACCGGGCTGATCGACGTGGGGCGGGTCGACCGTGCGGACCGGCACTcCGATCTCGCGCTGTGCTGCgCGAANNTGGCCCACGAAGAGGACCCGTGGTTCGGGCCCGGAGTGTTCCGCGGCGTTCCTGCGGGGAGTACGGGCGCGGGTGGGATGGGGCGGTATCGGAGGAAAAGCTGGCGTTTTACCGGCTGNTGGACGAGTTCTTCTGAGGGACCTGATGGTGTTGGTGGCTGGGTAGGGTTGCGTCGCGTGGGTGACAGCACAgtGTgGACGtTGGGATCCGGCAAGACtGGCCCCGCTTGGCAACGCAACAGTGAGCCCCTCCCTAGTGTGTTTGGGGATGTGACTATGTATTCGTGTGTTGGCCAACGGGTCAACCCGAACAGATTGATACCCGCCTTGGCATTTCCTGTCAGAATGTAACGTCAGTTGATGGTACCAGCAGCCGGTGGTGTACCAGACCAATGCCGCTGCGGGCGCCAGTAAGNTCAGTCGCGCGCCCAGCCGCGGCGACCCCTGGCAACAGTCGGGCGGTGGGCAGCAAGGGCGCGGCGCGATGCCGCCGCTGCCGCCCGGCGGTGGGCCGCGCATGAGCGGGCACTGGGACGACGACGGCGGCAACCCGGAGCGGCCGTACTCGCGGGGGATGCTGGGCGGCGGCGGCGGGCCCATGCAGCCGGGCTTTGGGCAGAACCAGATGTGGCCGCAGCTCAACGTGCAGCAGCAGCAACAGCAGCAGCGGAGGGGCAATTACGGCCTGTCGCGATACCCCTACGACGTGCCCGACTACGCCTACCCCTACGACGTGCCCGACTACGCCGATCGATCCGGACCGTACCCCTACGACGTGCCCGACTACGCCGCTTAGCACAGTGACACTTACTGTGACGGCTTGCTTGCTAGAGGGGCAGCAGCTTCCCTACGCAAGCCCGGTTGTGTGCAGCGGGGCTGACAATGTCGGCGACAACAAAAGGACTGGACTG

**Part II: Sequence of PCR product from seven strains that yielded product of expected size (related to Figure 7 of main text)**

CACGAGTATCGCGTCGTCTGGGCAGCCGGTGCTGTACCAGACCAATGCGGCTGCGGGCGCCAGTAAGCTCAGTCGCGCGCCCAGCCGCGGCGACCCCTGGCAACAGTCGGGCGGTGGGCAGCAAGGGCGCGGCGCGATGCCGCCGCTGCCGCCCGGCGGTGGGCCGCGCATGAGCGGGCACTGGGACGACGACGGCGGCAACCCGGAGCGGCCGTACTCGCGGGGGATGCTGGGCGGCGGCGGCGGGCCCATGCAGCCGGGCTTTGGGCAGAACCAGATGTGGCCGCAGCTCAACGTGCAGCAGCAGCAACAGCAGCAGCGGAGGGGCAATTACGGCCTGTCGCGATACCCCTACGACGTGCCCGACTACGCCTACCCCTACGACGTGCCCGACTACGCCGATCGATCCGGACCGTACCCCTACGACGTGCCCGACTACGCCGCTTAGCACAGTGACACTTACTGTGACGGCTTGCTTGCTAGAGGGGCAGCAGCTTCCCTACGCAAGCCCGGTTGTGTGCAGCGGGGCTGACAATGTCGGCGACAACAAAAGGACTGGACTGTACGGTACTTTCTGGTGGTGGATGCAGCCTCACTGCTAAAGACGCGGTCGTTTGCGCACTTCCGGCGTCCTGGTGGACTGCGGCTGACCAGCATTTGGCTCTGTGCAGCCGCTGACTGCGGCTTCGCTATTGTCACTGAAGCTCGATGACTACAGCTGGCGCCTGGGATTCTTAGCAGGATGCATGCGTCACGACATCCCTGGGGGGGACCGGGGAGGCGAGGGTTTGAGAGCGGGGCTTCCTATCTTGAACAGACATGTGAGGAGTGTGCATCATGAAGTAGGATGTCATTGTGATGACAAGAATGGCAGGACCGACAGGCGCCAACAGACACATGCGCCGTCGCCTGTTCGGGGGGGGGGGCGTCCTGCGGCACTGGTTTGCGGGCCAGCAGCTGCAGCTGACGGGCATGCATAAACAGTTGCACTTTTGAAACAGGAACGGTACGTATGGCGGCAAAGCAGGCTTTGTTTGGGACAACAAGACAAGACGCCCTGGGCAAGGGCTTTGTTAGCGGAATGCACCTGCGCGGCACCGCACTTGAGGACGTATGCACATGCGACTTGTTGCGTCCACGAGCTTGGCAGTGTTGACGCGCTGAAAGTAGCGGCGGTGGCATCTGAGAGCGGCCGTGACAAGGGGAGGAGGCATAGGCGTGTTGCTGTCGCACGCCGTCTAACAAGGGGACGCGCCGGCATTGCCATGGGCGGGACGCACGCGTGAGACCTTGCCGCTGACGCTGCGTGGTCATTGACCACATTTGGACGTGATACGGGATTTGGTGGGAGTTTGGTCTCTTCTTCTTACTCTTAAACGCTACTATGGGGGCGGGAAGGGGTGCGCATGCATAGCAATAAGCACGTTTGGCGTGAGAGGCAAAGATTGCGTCACACCCCTGCGCTTCTCAAAGCGAGTGGGCTTTCTTTCATTCTGGTTCTCGTCTTCCTACTTCTCTAGCCGGGGCTCGCAGTACAGCCCTGGCGCTACCCAATTGGCTAACATCGTTTGTAGACTGACGCTGTTGTGTTAGTTGGGGGCACGGTACGGGGGGCATGGTCGTGATGTGTCCACCACGCAATGTGCCACATGGCACCTTGCGTGGCACAGCGAAACGCTAACAAAGGTTCTGGAGTTCGTGATCCGTACACGTGCATGTAATGGACCAATGCTATCGCTTGAATGATTGTGATCACCGATTGAGCGTTGACTAGGAGTTCGAGCC
