# Supplementary material for: Direct in situ protein tagging in Chlamydomonas reinhardtii utilizing TIM, a method for CRISPR/Cas9-based targeted insertional mutagenesis
Source: PLoS One. 2022 Dec 9;17(12):e0278972. doi: 10.1371/journal.pone.0278972 (PMC9733891; doi:10.1371/journal.pone.0278972)
Supplement: S2 Appendix — (DOCX) [file pone.0278972.s002.docx]

**S2 Appendix. Sequences of PCR products amplified from the region surrounding the 3’ end of the inserted donor DNA in LF5-HA strains (related to Fig 6 and 7 of main text)**

**Part I: The region surrounding the site where the 3’ end of the donor DNA integrated was amplified from the eight HA-tagged strains**


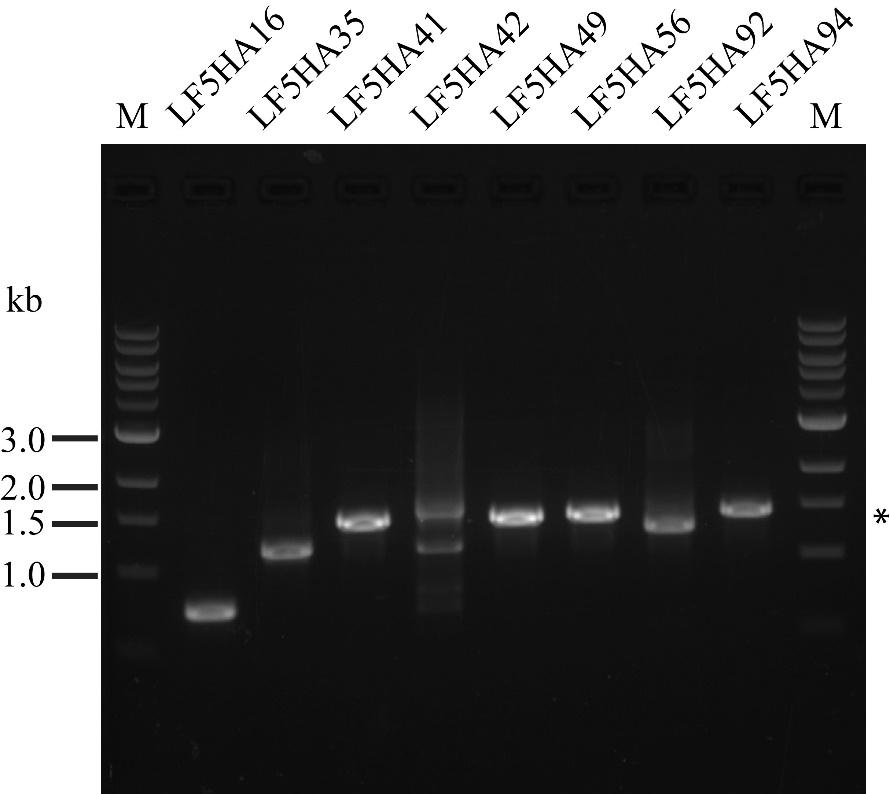


Gel image of PCR products amplified with primer pair AphVIII-3/LF5HA-28 using genomic DNA from the different strains as template. M: 1 kb DNA ladder from NEB (Catalog number NO552). * indicates size expected for a perfect integration event.

**Part II: Sequences of the PCR products**

Primer AphVIII-3: GTCGGTATCCCGGTTGTGA

Primer LF5-28: CAGTCCAGTCCTTTTGTTGTCG

Green highlight: Paromomycin sequence

Arrowhead: Site where the 3’ end of the donor DNA would integrate into the DSB in a perfect insertion event (shown only for expected sequence).

Sequence expected for a perfect insertion:

GTCGGTATCCCGGTTGTGAGTGGGTTGTTGTGGAGGATGGGGCCTCGGGGGCTGGTGTTTATCGGCTTCGGGGTGGTGGGCGGGAGTTGTTTGTCAAGGTGGCAGCTCTGGGGGCCGGGGTGGGCTTGTTGGGTGAGGCTGAgCGGCTGGTGTGGTTGGCGGAGGTGGGGATTCCCGTACCTCGTGTTGTGGAGGGTGGTGGGGACGAGAGGGTCGCCTGGTTGGTCACCGAAGCGGTTCCGGGGCGTCCGGCCAGTGCGCGGTGGCCGCGGGAGCAGCGGCTGGACGTGGCGGTGGCGCTCGCGGGGCTCGCTCGTTCGCTGCACGCGCTGGACTGGGAGCGGTGTCCGTTCGATCGCAGTCTCGCGGTGACGGTGCCGCAGGCGGCCCGTGCTGTCGCTGAAGGGAGCGTCGACTTGGAGGATCTGGACGAGGAGCGGAAGGGGTGGTCGGGGGAGCGGCTTCTCGCCGAGCTGGAGCGGACTCGGCCTGCGGACGAGGATCTGGCGGTTTGCCACGGTGACCTGTGCCCGGACAACGTGCTGCTCGACCCTCGTACCTGCGAGGTGACCGGGCTGATCGACGTGGGGCGGGTCGGCCGTGCGGACCGGCACTCCGATCTCGCGCTGGTGCTGCGCGAGCTGGCCCACGAGGAGGACCCGTGGTTCGGGCCGGAGTGTTCCGCGGCGTTCCTGCGGGAGTACGGGCGCGGGTGGGATGGGGCGGTATCGGAGGAAAAGCTGGCGTTTTACCGGCTGTTGGACGAGTTCTTCTGAGGGACCTGATGGTGTTGGTGGCTGGGTAGGGTTGCGTCGCGTGGGTGACAGCACAGTGTGGACGTTGGGATCCGGCAAGACTGGCCCCGCTTGGCAACGCAACAGTGAGCCCCTCCCTAGTGTGTTTGGGGATGTGACTATGTATTCGTGTGTTGGCCAACGGGTCAACCCGAACAGATTGATAC

CCGCCTTGGCATTTCCTGTCAGAATGTAACGTCAGTTGATGGTACCAGCATGCGGCTGCGGGCGCCAGTAAGCTCAGTCGCGCGCCCAGCCGCGGCGACCCCTGGCAACAGTCGGGCGGTGGGCAGCAAGGGCGCGGCGCGATGCCGCCGCTGCCGCCCGGCGGTGGGCCGCGCATGAGCGGGCACTGGGACGACGACGGCGGCAACCCGGAGCGGCCGTACTCGCGGGGGATGCTGGGCGGCGGCGGCGGGCCCATGCAGCCGGGCTTTGGGCAGAACCAGATGTGGCCGCAGCTCAACGTGCAGCAGCAGCAACAGCAGCAGCGGAGGGGCAATTACTAGTAGCACAGTGACACTTACTGTGACGGCTTGCTTGCTAGAGGGGCAGCAGCTTCCCTACGCAAGCCCGGTTGTGTGCAGCGGGGCTGACAATGTCGGCGACAACAAAAGGACTGGACTG

Strain LF5HA16

GTCGGTATCCCGGTTGTGAGTGGGTTGTTGTGGAGGATGGGGCCTCGGGGGCTGGTGTTTATCGGCTTCGGGGTGGTGGGCGGGAGTTGTTTGTCAAGGTGGCAGCTCTGGGGGCCGGGGTGGGCTTGTTGGGTGAGGCTGAGCGGCTGGTGTGGTTGGCGGAGGTGGGGATTCCCGTACCTCGTGTTGTGGACGGTGGTGTACCAGACCAATGCCGCTGCGGGCGCCAGTAAGCTCAGTCGCGCGCCCAGCCGCGGCGACCCCTGGCAACAGTCGGGCGGTGGGCAGCAAGGGCGCGGCGCGATGCCGCCGCTGCCGCCCGGCGGTGGGCCGCGCATGAGCGGGCACTGGGACGACGACGGCGGCAACCCGGAGCGGCCGTACTCGCGGGGGATGCTGGGCGGCGGCGGCGGGCCCATGCAGCCGGGCTTTGGGCAGAACCAGATGTGGCCGCAGCTCAACGTGCAGCAGCAGCAACAGCAGCAGCGGAGGGGCAATTACGGCCTGTCGCGATACCCCTACGACGTGCCCGACTACGCCTACCCCTACGACGTGCCCGACTACGCCGATCGATCCGGACCGTACCCCTACGACGTGCCCGACTACGCCGCTTAGCACAGTGACACTTACTGTGACGGCTTGCTTGCTAGAGGGGCAGCAGCTTCCCTACGCAAGCCCGGTTGTGTGCAGCGGGGCTGACAATGTCGGCGACAACAAAAGGACTGGACTG

Strain LF5HA35

GTCGGTATCCCGGTTGTGAGTGGGTTGTTGTGGAGGATGGGGCCTCGGGGGCTGGTGTTTATCGGCTTCGGGGTGGTGGGCGGGAGTTGTTTGTCAAGGTGGCAGCTCTGGGGGCCGGGGTGGGCTTGTTGGGTGAGGCTGAGCGGCTGGTGTGGTTGGCGGAGGTGGGGATTCCCGTACCTCGTGTTGTGGAGGGTGGTGGGGACGAGAGGGTCGCCTGGTTGGTCACCGAAGCGGTTCCGGGGCGTCCGGCCAGTGCGCGGTGGCCGCGGGAGCAGCGGCTGGACGTGGCGGTGGCGCTCGCGGGGCTCGCTCGTTCGCTGCACGCGCTGGACTGGGAGCGGTGTCCGTTCGATCGCAGTCTCGCGGTGACGGTGCCGCAGGCGGCCCGTGCTGTCGCTGAAGGGAGCGTCGACTTGGAGGATCTGGACGAGGAGCGGAAGGGGTGGTCGGGGGAGCGGCTTCTCGCCGAGCTGGAGCGGACTCGGCCTGCGGACGAGGATGGGGCGGCAAGGCCGGCGCGGGCGGGCAGCAGCGGCTCAACGCCAGCCCGCCGCAGATGCACATGGACGGCGGTGGGTACGCGTCAGGTGGCCGCACGAGTATCGCGTCGTCTGGGCAGCCGGTGCTGTACCAGACCAATGCCGCTGCGGGCGCCAGTAAGCTCAGTCGCGCGCCCAGCCGCGGCGACCCCTGGCAACAGTCGGGCGGTGGGCAGCAAGGGCGCGGCGCGATGCCGCCGCTGCCGCCCGGCGGTGGGCCGCGCATGAGCGGGCACTGGGACGACGACGGCGGCAACCCGGAGCGGCCGTACTCGCGGGGGATGCTGGGCGGCGGCGGCGGGCCCATGCAGCCGGGCTTTGGGCAGAACCAGATGTGGCCGCAGCTCAACGTGCAGCAGCAGCAACAGCAGCAGCGGAGGGGCAATTACGGCCTGTCGCGATACCCCTACGACGTGCCCGACTACGCCTACCCCTACGACGTGCCCGACTACGCCGATCGATCCGGACCGTACCCCTACGACGTGCCCGACTACGCCGCTTAGCACAGTGACACTTACTGTGACGGCTTGCTTGCTAGAGGGGCAGCAGCTTCCCTACGCAAGCCCGGTTGTGTGCAGCGGGGCTGACAATGTCGGCGACAACAAAAGGACTGGACTG

Strain LF5HA41

GTCGGTATCCCGGTTGTGAGTGGGTTGTTGTGGAGGATGGGGCCTCGGGGGCTGGTGTTTATCGGCTTCGGGGTGGTGGGCGGGAGTTGTTTGTCAAGGTGGCAGCTCTGGGGGCCGGGGTGGGCTTGTTGGGTGAGGCTGAGCGGCTGGTGTGGTTGGCGGAGGTGGGGATTCCCGTACCTCGTGTTGTGGAGGGTGGTGGGGACGAGAGGGTCGCCTGGTTGGTCACCGAAGCGGTTCCGGGGCGTCCGGCCAGTGCGCGGTGGCCGCGGGAGCAGCGGCTGGACGTGGCGGTGGCGCTCGCGGGGCTCGCTCGTTCGCTGCACGCGCTGGACTGGGAGCGGTGTCCGTTCGATCGCAGTCTCGCGGTGACGGTGCCGCAGGCGGCCCGTGCTGTCGCTGAAGGGAGCGTCGACTTGGAGGATCTGGACGAGGAGCGGAAGGGGTGGTCGGGGGAGCGGCTTCTCGCCGAGCTGGAGCGGACTCGGCCTGCGGACGAGGATCTGGCGGTTTGCCACGGTGACCTGTGCCCGGACAACGTGCTGCTCGACCCTCGTACCTGCGAGGTGACCGGGCTGATCGACGTGGGGCGGGTCGGCCGTGCGGACCGGCACTCCGATCTCGCGCTGGTGCTGCGCGAGCTGGCCCACGAGGAGGACCCGTGGTTCGGGCCGGAGTGTTCCGCGGCGTTCCTGCGGGAGTACGGGCGCGGGTGGGATGGGGCGGTATCGGAGGAAAAGCTGGCGTTTTACCGGCTGTTGGACGAGTTCTTCTGAGGGACCTGATGGTGTTGGTGGCTGGGTAGGGTTGCGTCGCGTGGGTGACAGCACAGTGTGGACGTTGGGATCCGGCAAGACTGGCCCCGCTTGGCAACGCAACAGTGAGCCCCTCCCTAGTGTGTTTGGGGATGTGACTATGTATTCGTGTGTTGGCCAACGGGTCAACCCGAACAGATTGATACCCGCCTTGGCATTTCCTGTCAGAATGTAACGTCAGTTGATGGTACCAGCAATGCGGCTGCGGGCGCCAGTAAGCTCAGTCGCGCGCCCAGCCGCGGCGACCCCTGGCAACAGTCGGGCGGTGGGCAGCAAGGGCGCGGCGCGATGCCGCCGCTGCCGCCCGGCGGTGGGCCGCGCATGAGCGGGCACTGGGACGACGACGGCGGCAACCCGGAGCGGCCGTACTCGCGGGGGATGCTGGGCGGCGGCGGCGGGCCCATGCAGCCGGGCTTTGGGCAGAACCAGATGTGGCCGCAGCTCAACGTGCAGCAGCAGCAACAGCAGCAGCGGAGGGGCAATTACTAGTAGCACAGTGACACTTACTGTGACGGCTTGCTTGCTAGAGGGGCAGCAGCTTCCCTACGCAAGCCCGGTTGTGTGCAGCGGGGCTGACAATGTCGGCGACAACAAAAGGACTGGACTG

Strain LF5HA49

GTCGGTATCCCGGTTGTGAGTGGGTTGTTGTGGAGGATGGGGCCTCGGGGGCTGGTGTTTATCGGCTTCGGGGTGGTGGGCGGGAGTTGTTTGTCAAGGTGGCAGCTCTGGGGGCCGGGGTGGGCTTGTTGGGTGAGGCTGAgCGGCTGGTGTGGTTGGCGGAGGTGGGGATTCCCGTACCTCGTGTTGTGGAGGGTGGTGGGGACGAGAGGGTCGCCTGGTTGGTCACCGAAGCGGTTCCGGGGCGTCCGGCCAGTGCGCGGTGGCCGCGGGAGCAGCGGCTGGACGTGGCGGTGGCGCTCGCGGGGCTCGCTCGTTCGCTGCACGCGCTGGACTGGGAGCGGTGTCCGTTCGATCGCAGTCTCGCGGTGACGGTGCCGCAGGCGGCCCGTGCTGTCGCTGAAGGGAGCGTCGACTTGGAGGATCTGGACGAGGAGCGGAAGGGGTGGTCGGGGGAGCGGCTTCTCGCCGAGCTGGAGCGGACTCGGCCTGCGGACGAGGATCTGGCGGTTTGCCACGGTGACCTGTGCCCGGACAACGTGCTGCTCGACCCTCGTACCTGCGAGGTGACCGGGCTGATCGACGTGGGGCGGGTCGGCCGTGCGGACCGGCACTCCGATCTCGCGCTGGTGCTGCGCGAGCTGGCCCACGAGGAGGACCCGTGGTTCGGGCCGGAGTGTTCCGCGGCGTTCCTGCGGGAGTACGGGCGCGGGTGGGATGGGGCGGTATCGGAGGAAAAGCTGGCGTTTTACCGGCTGTTGGACGAGTTCTTCTGAGGGACCTGATGGTGTTGGTGGCTGGGTAGGGTTGCGTCGCGTGGGTGACAGCACAGTGTGGACGTTGGGATCCGGCAAGACTGGCCCCGCTTGGCAACGCAACAGTGAGCCCCTCCCTAGTGTGTTTGGGGATGTGACTATGTATTCGTGTGTTGGCCAACGGGTCAACCCGAACAGATTGATACCCGCCTTGGCATTTCCTGTCAGAATGTAACGTCAGTTGATGGTACCAGCGGCTGCGGGCGCCAGTAAGCTCAGTCGCGCGCCCAGCCGCGGCGACCCCTGGCAACAGTCGGGCGGTGGGCAGCAAGGGCGCGGCGCGATGCCGCCGCTGCCGCCCGGCGGTGGGCCGCGCATGAGCGGGCACTGGGACGACGACGGCGGCAACCCGGAGCGGCCGTACTCGCGGGGGATGCTGGGCGGCGGCGGCGGGCCCATGCAGCCGGGCTTTGGGCAGAACCAGATGTGGCCGCAGCTCAACGTGCAGCAGCAGCAACAGCAGCAGCGGAGGGGCAATTACTAGTAGCACAGTGACACTTACTGTGACGGCTTGCTTGCTAGAGGGGCAGCAGCTTCCCTACGCAAGCCCGGTTGTGTGCAGCGGGGCTGACAATGTCGGCGACAACAAAAGGACTGGACTG

Strain LF5HA56

GTCGGTATCCCGGTTGTGAGTGGGTTGTTGTGGAGGATGGGGCCTCGGGGGCTGGTGTTTATCGGCTTCGGGGTGGTGGGCGGGAGTTGTTTGTCAAGGTGGCAGCTCTGGGGGCCGGGGTGGGCTTGTTGGGTGAGGCTGAGCGGCTGGTGTGGTTGGCGGAGGTGGGGATTCCCGTACCTCGTGTTGTGGAGGGTGGTGGGGACGAGAGGGTCGCCTGGTTGGTCACCGAAGCGGTTCCGGGGCGTCCGGCCAGTGCGCGGTGGCCGCGGGAGCAGCGGCTGGACGTGGCGGTGGCGCTCGCGGGGCTCGCTCGTTCGCTGCACGCGCTGGACTGGGAGCGGTGTCCGTTCGATCGCAGTCTCGCGGTGACGGTGCCGCAGGCGGCCCGTGCTGTCGCTGAAGGGAGCGTCGACTTGGAGGATCTGGACGAGGAGCGGAAGGGGTGGTCGGGGGAGCGGCTTCTCGCCGAGCTGGAGCGGACTCGGCCTGCGGACGAGGATCTGGCGGTTTGCCACGGTGACCTGTGCCCGGACAACGTGCTGCTCGACCCTCGTACCTGCGAGGTGACCGGGCTGATCGACGTGGGGCGGGTCGGCCGTGCGGACCGGCACTCCGATCTCGCGCTGGTGCTGCGCGAGCTGGCCCACGAGGAGGACCCGTGGTTCGGGCCGGAGTGTTCCGCGGCGTTCCTGCGGGAGTACGGGCGCGGGTGGGATGGGGCGGTATCGGAGGAAAAGCTGGCGTTTTACCGGCTGTTGGACGAGTTCTTCTGAGGGACCTGATGGTGTTGGTGGCTGGGTAGGGTTGCGTCGCGTGGGTGACAGCACAGTGTGGACGTTGGGATCCGGCAAGACTGGCCCCGCTTGGCAACGCAACAGTGAGCCCCTCCCTAGTGTGTTTGGGGATGTGACTATGTATTCGTGTGTTGGCCAACGGGTCAACCCGAACAGATTGATACCCGCCTTGGCATTTCCTGTCAGAATGTAACGTCAGTTGATGGTACCAGCCGGTGCGCCAGCAGCTGCGGGCGCCAGTAAGCTCAGTCGCGCGCCCAGCCGCGGCGACCCCTGGCAACAGTCGGGCGGTGGGCAGCAAGGGCGCGGCGCGATGCCGCCGCTGCCGCCCGGCGGTGGGCCGCGCATGAGCGGGCACTGGGACGACGACGGCGGCAACCCGGAGCGGCCGTACTCGCGGGGGATGCTGGGCGGCGGCGGCGGGCCCATGCAGCCGGGCTTTGGGCAGAACCAGATGTGGCCGCAGCTCAACGTGCAGCAGCAGCAACAGCAGCAGCGGAGGGGCAATTACTAGTAGCACAGTGACACTTACTGTGACGGCTTGCTTGCTAGAGGGGCAGCAGCTTCCCTACGCAAGCCCGGTTGTGTGCAGCGGGGCTGACAATGTCGGCGACAACAAAAGGACTGGACTG

Strain LF5HA92

GTCGGTATCCCGGTTGTGAGTGGGTTGTTGTGGAGGATGGGGCCTCGGGGGCTGGTGTTTATCGGCTTCGGGGTGGTGGGCGGGAGTTGTTTGTCAAGGTGGCAGCTCTGGGGGCCGGGGTGGGCTTGTTGGGTGAGGCTGAGCGGCTGGTGTGGTTGGCGGAGGTGGGGATTCCCGTACCTCGTGTTGTGGAGGGTGGTGGGGACGAGAGGGTCGCCTGGTTGGTCACCGAAGCGGTTCCGGGGCGTCCGGCCAGTGCGCGGTGGCCGCGGGAGCAGCGGCTGGACGTGGCGGTGGCGCTCGCGGGGCTCGCTCGTTCGCTGCACGCGCTGGACTGGGAGCGGTGTCCGTTCGATCGCAGTCTCGCGGTGACGGTGCCGCAGGCGGCCCGTGCTGTCGCTGAAGGGAGCGTCGACTTGGAGGATCTGGACGAGGAGCGGAAGGGGTGGTCGGGGGAGCGGCTTCTCGCCGAGCTGGAGCGGACTCGGCCTGCGGACGAGGATCTGGCGGTTTGCCACGGTGACCTGTGCCCGGACAACGTGCTGCTCGACCCTCGTACCTGCGAGGTGACCGGGCTGATCGACGTGGGGCGGGTCGGCCGTGCGGACCGGCACTCCGATCTCGCGCTGGTGCTGCGCGAGCTGGCCCACGAGGAGGACCCGTGGTTCGGGCCGGAGTGTTCCGCGGCGTTCCTGCGGGAGTACGGGCGCGGGTGGGATGGGGCGGTATCGGAGGAAAAGCTGGCGTTTTACCGGCTGTTGGACGAGTTCTTCTGAGGGACCTGATGGTGTTGGTGGCTGGGTAGGGTTGCGTCGCGTGGGTGACAGCACAGTGTGGACGTTGGGATCCGGCAAGACTGGCCCCGCTTGGCAACGCAACAGTGAGCCCCTCCCTAGTGTGTTTGGGGATGTGACTATGTATTCGTGTGTTGGCCAACGGGTCAACCCGAACAGATTGATACCCGCCTTGGCATTTCCTGTCAGAATGTAACGTCAGTTGATGGTACCAGCCAGCAGCAACAGCAGCAGCGGAGGGGCAATTACGGCCTGTCGCGATACCCCTACGACGTGCCCGACTACGCCTACCCCTACGACGTGCCCGACTACGCCGATCGATCCGGACCGTACCCCTACGACGTGCCCGACTACGCCGCTTAGCACAGTGACACTTACTGTGACGGCTTGCTTGCTAGAGGGGCAGCAGCTTCCCTACGCAAGCCCGGTTGTGTGCAGCGGGGCTGACAATGTTGGCGACAACAAAAGGACTGGACTG

Strain LF5HA94

GTCGGTATCCCGGTTGTGAGTGGGTTGTTGTGGAGGATGGGGCCTCGGGGGCTGGTGTTTATCGGCTTCGGGGTGGTGGGCGGGAGTTGTTTGTCAAGGTGGCAGCTCTGGGGGCCGGGGTGGGCTTGTTGGGTGAGGCTGAGCGGCTGGTGTGGTTGGCGGAGGTGGGGATTCCCGTACCTCGTGTTGTGGAGGGTGGTGGGGACGAGAGGGTCGCCTGGTTGGTCACCGAAGCGGTTCCGGGGCGTCCGGCCAGTGCGCGGTGGCCGCGGGAGCAGCGGCTGGACGTGGCGGTGGCGCTCGCGGGGCTCGCTCGTTCGCTGCACGCGCTGGACTGGGAGCGGTGTCCGTTCGATCGCAGTCTCGCGGTGACGGTGCCGCAGGCGGCCCGTGCTGTCGCTGAAGGGAGCGTCGACTTGGAGGATCTGGACGAGGAGCGGAAGGGGTGGTCGGGGGAGCGGCTTCTCGCCGAGCTGGAGCGGACTCGGCCTGCGGACGAGGATCTGGCGGTTTGCCACGGTGACCTGTGCCCGGACAACGTGCTGCTCGACCCTCGTACCTGCGAGGTGACCGGGCTGATCGACGTGGGGCGGGTCGGCCGTGCGGACCGGCACTCCGATCTCGCGCTGGTGCTGCGCGAGCTGGCCCACGAGGAGGACCCGTGGTTCGGGCCGGAGTGTTCCGCGGCGTTCCTGCGGGAGTACGGGCGCGGGTGGGATGGGGCGGTATCGGAGGAAAAGCTGGCGTTTTACCGGCTGTTGGACGAGTTCTTCTGAGGGACCTGATGGTGTTGGTGGCTGGGTAGGGTTGCGTCGCGTGGGTGACAGCACAGTGTGGACGTTGGGATCCGGCAAGACTGGCCCCGCTTGGCAACGCAACAGTGAGCCCCTCCCTAGTGTGTTTGGGGATGTGACTATGTATTCGTGTGTTGGCCAACGGGTCAACCCGAACAGATTGATACCCGCCTTGGCATTTCCTGTCAGAATGTAACGTCAGTTGATGGTACCAGCGGCGCGATGCCGCCGCTGCCGCCCGGCGGTGGGCCGCGCATGAGCGGGCACTGGGACGACGACGGCGGCAACCCGGAGCGGCCGTACTCGCGGGGGATGCTGGGCGGCGGCGGCGGGCCCATGCAGCCGGGCTTTGGGCAGAACCAGATGTGGCCGCAGCTCAACGTGCAGCAGCAGCAACAGCAGCAGCGGAGGGGCAATTACGGCCTGTCGCGATACCCCTACGACGTGCCCGACTACGCCTACCCCTACGACGTGCCCGACTACGCCGATCGATCCGGACCGTACCCCTACGACGTGCCCGACTACGCCGCTTAGCACAGTGACACTTACTGTGACGGCTTGCTTGCTAGAGGGGCAGCAGCTTCCCTACGCAAGCCCGGTTGTGTGCAGCGGGGCTGACAATGTCGGCGACAACAAAAGGACTGGACTG

**Part III: Sequence alignment generated using MUSCLE (SnapGene version 6.1.1)**

Yellow highlight: Sequence identical to that expected for a perfect integration event.

Arrowhead: The site where the 3’ end of the donor DNA would insert in a perfect integration event.

*: 3HA sequence.

Expected sequence 1 GTCGGTATCCCGGTTGTGAGTGGGTTGTTGTGGAGGATGGGGCCTCGGGGGCTGGTGTTT
Strain LF5HA41 1 GTCGGTATCCCGGTTGTGAGTGGGTTGTTGTGGAGGATGGGGCCTCGGGGGCTGGTGTTT
Strain LF5HA49 1 GTCGGTATCCCGGTTGTGAGTGGGTTGTTGTGGAGGATGGGGCCTCGGGGGCTGGTGTTT
Strain LF5HA56 1 GTCGGTATCCCGGTTGTGAGTGGGTTGTTGTGGAGGATGGGGCCTCGGGGGCTGGTGTTT
Strain LF5HA92 1 GTCGGTATCCCGGTTGTGAGTGGGTTGTTGTGGAGGATGGGGCCTCGGGGGCTGGTGTTT
Strain LF5HA94 1 GTCGGTATCCCGGTTGTGAGTGGGTTGTTGTGGAGGATGGGGCCTCGGGGGCTGGTGTTT
Strain LF5HA35 1 GTCGGTATCCCGGTTGTGAGTGGGTTGTTGTGGAGGATGGGGCCTCGGGGGCTGGTGTTT
Strain LF5HA16 1 GTCGGTATCCCGGTTGTGAGTGGGTTGTTGTGGAGGATGGGGCCTCGGGGGCTGGTGTTT

Expected sequence 61 ATCGGCTTCGGGGTGGTGGGCGGGAGTTGTTTGTCAAGGTGGCAGCTCTGGGGGCCGGGG
Strain LF5HA41 61 ATCGGCTTCGGGGTGGTGGGCGGGAGTTGTTTGTCAAGGTGGCAGCTCTGGGGGCCGGGG
Strain LF5HA49 61 ATCGGCTTCGGGGTGGTGGGCGGGAGTTGTTTGTCAAGGTGGCAGCTCTGGGGGCCGGGG
Strain LF5HA56 61 ATCGGCTTCGGGGTGGTGGGCGGGAGTTGTTTGTCAAGGTGGCAGCTCTGGGGGCCGGGG
Strain LF5HA92 61 ATCGGCTTCGGGGTGGTGGGCGGGAGTTGTTTGTCAAGGTGGCAGCTCTGGGGGCCGGGG
Strain LF5HA94 61 ATCGGCTTCGGGGTGGTGGGCGGGAGTTGTTTGTCAAGGTGGCAGCTCTGGGGGCCGGGG
Strain LF5HA35 61 ATCGGCTTCGGGGTGGTGGGCGGGAGTTGTTTGTCAAGGTGGCAGCTCTGGGGGCCGGGG
Strain LF5HA16 61 ATCGGCTTCGGGGTGGTGGGCGGGAGTTGTTTGTCAAGGTGGCAGCTCTGGGGGCCGGGG

Expected sequence 121 TGGGCTTGTTGGGTGAGGCTGAGCGGCTGGTGTGGTTGGCGGAGGTGGGGATTCCCGTAC
Strain LF5HA41 121 TGGGCTTGTTGGGTGAGGCTGAGCGGCTGGTGTGGTTGGCGGAGGTGGGGATTCCCGTAC
Strain LF5HA49 121 TGGGCTTGTTGGGTGAGGCTGAGCGGCTGGTGTGGTTGGCGGAGGTGGGGATTCCCGTAC
Strain LF5HA56 121 TGGGCTTGTTGGGTGAGGCTGAGCGGCTGGTGTGGTTGGCGGAGGTGGGGATTCCCGTAC
Strain LF5HA92 121 TGGGCTTGTTGGGTGAGGCTGAGCGGCTGGTGTGGTTGGCGGAGGTGGGGATTCCCGTAC
Strain LF5HA94 121 TGGGCTTGTTGGGTGAGGCTGAGCGGCTGGTGTGGTTGGCGGAGGTGGGGATTCCCGTAC
Strain LF5HA35 121 TGGGCTTGTTGGGTGAGGCTGAGCGGCTGGTGTGGTTGGCGGAGGTGGGGATTCCCGTAC
Strain LF5HA16 121 TGGGCTTGTTGGGTGAGGCTGAGCGGCTGGTGTGGTTGGCGGAGGTGGGGATTCCCGTAC

Expected sequence 181 CTCGTGTTGTGGAGGGTGGTGGGGACGAGAGGGTCGCCTGGTTGGTCACCGAAGCGGTTC
Strain LF5HA41 181 CTCGTGTTGTGGAGGGTGGTGGGGACGAGAGGGTCGCCTGGTTGGTCACCGAAGCGGTTC
Strain LF5HA49 181 CTCGTGTTGTGGAGGGTGGTGGGGACGAGAGGGTCGCCTGGTTGGTCACCGAAGCGGTTC
Strain LF5HA56 181 CTCGTGTTGTGGAGGGTGGTGGGGACGAGAGGGTCGCCTGGTTGGTCACCGAAGCGGTTC
Strain LF5HA92 181 CTCGTGTTGTGGAGGGTGGTGGGGACGAGAGGGTCGCCTGGTTGGTCACCGAAGCGGTTC
Strain LF5HA94 181 CTCGTGTTGTGGAGGGTGGTGGGGACGAGAGGGTCGCCTGGTTGGTCACCGAAGCGGTTC
Strain LF5HA35 181 CTCGTGTTGTGGAGGGTGGTGGGGACGAGAGGGTCGCCTGGTTGGTCACCGAAGCGGTTC
Strain LF5HA16 181 CTCGTGTTGTGGACGGTGGT----------------------------------------

Expected sequence 241 CGGGGCGTCCGGCCAGTGCGCGGTGGCCGCGGGAGCAGCGGCTGGACGTGGCGGTGGCGC
Strain LF5HA41 241 CGGGGCGTCCGGCCAGTGCGCGGTGGCCGCGGGAGCAGCGGCTGGACGTGGCGGTGGCGC
Strain LF5HA49 241 CGGGGCGTCCGGCCAGTGCGCGGTGGCCGCGGGAGCAGCGGCTGGACGTGGCGGTGGCGC
Strain LF5HA56 241 CGGGGCGTCCGGCCAGTGCGCGGTGGCCGCGGGAGCAGCGGCTGGACGTGGCGGTGGCGC
Strain LF5HA92 241 CGGGGCGTCCGGCCAGTGCGCGGTGGCCGCGGGAGCAGCGGCTGGACGTGGCGGTGGCGC
Strain LF5HA94 241 CGGGGCGTCCGGCCAGTGCGCGGTGGCCGCGGGAGCAGCGGCTGGACGTGGCGGTGGCGC
Strain LF5HA35 241 CGGGGCGTCCGGCCAGTGCGCGGTGGCCGCGGGAGCAGCGGCTGGACGTGGCGGTGGCGC
Strain LF5HA16 200 ------------------------------------------------------------

Expected sequence 301 TCGCGGGGCTCGCTCGTTCGCTGCACGCGCTGGACTGGGAGCGGTGTCCGTTCGATCGCA
Strain LF5HA41 301 TCGCGGGGCTCGCTCGTTCGCTGCACGCGCTGGACTGGGAGCGGTGTCCGTTCGATCGCA
Strain LF5HA49 301 TCGCGGGGCTCGCTCGTTCGCTGCACGCGCTGGACTGGGAGCGGTGTCCGTTCGATCGCA
Strain LF5HA56 301 TCGCGGGGCTCGCTCGTTCGCTGCACGCGCTGGACTGGGAGCGGTGTCCGTTCGATCGCA
Strain LF5HA92 301 TCGCGGGGCTCGCTCGTTCGCTGCACGCGCTGGACTGGGAGCGGTGTCCGTTCGATCGCA
Strain LF5HA94 301 TCGCGGGGCTCGCTCGTTCGCTGCACGCGCTGGACTGGGAGCGGTGTCCGTTCGATCGCA
Strain LF5HA35 301 TCGCGGGGCTCGCTCGTTCGCTGCACGCGCTGGACTGGGAGCGGTGTCCGTTCGATCGCA
Strain LF5HA16 200 ------------------------------------------------------------

Expected sequence 361 GTCTCGCGGTGACGGTGCCGCAGGCGGCCCGTGCTGTCGCTGAAGGGAGCGTCGACTTGG
Strain LF5HA41 361 GTCTCGCGGTGACGGTGCCGCAGGCGGCCCGTGCTGTCGCTGAAGGGAGCGTCGACTTGG
Strain LF5HA49 361 GTCTCGCGGTGACGGTGCCGCAGGCGGCCCGTGCTGTCGCTGAAGGGAGCGTCGACTTGG
Strain LF5HA56 361 GTCTCGCGGTGACGGTGCCGCAGGCGGCCCGTGCTGTCGCTGAAGGGAGCGTCGACTTGG
Strain LF5HA92 361 GTCTCGCGGTGACGGTGCCGCAGGCGGCCCGTGCTGTCGCTGAAGGGAGCGTCGACTTGG
Strain LF5HA94 361 GTCTCGCGGTGACGGTGCCGCAGGCGGCCCGTGCTGTCGCTGAAGGGAGCGTCGACTTGG
Strain LF5HA35 361 GTCTCGCGGTGACGGTGCCGCAGGCGGCCCGTGCTGTCGCTGAAGGGAGCGTCGACTTGG
Strain LF5HA16 200 ------------------------------------------------------------

Expected sequence 421 AGGATCTGGACGAGGAGCGGAAGGGGTGGTCGGGGGAGCGGCTTCTCGCCGAGCTGGAGC
Strain LF5HA41 421 AGGATCTGGACGAGGAGCGGAAGGGGTGGTCGGGGGAGCGGCTTCTCGCCGAGCTGGAGC
Strain LF5HA49 421 AGGATCTGGACGAGGAGCGGAAGGGGTGGTCGGGGGAGCGGCTTCTCGCCGAGCTGGAGC
Strain LF5HA56 421 AGGATCTGGACGAGGAGCGGAAGGGGTGGTCGGGGGAGCGGCTTCTCGCCGAGCTGGAGC
Strain LF5HA92 421 AGGATCTGGACGAGGAGCGGAAGGGGTGGTCGGGGGAGCGGCTTCTCGCCGAGCTGGAGC
Strain LF5HA94 421 AGGATCTGGACGAGGAGCGGAAGGGGTGGTCGGGGGAGCGGCTTCTCGCCGAGCTGGAGC
Strain LF5HA35 421 AGGATCTGGACGAGGAGCGGAAGGGGTGGTCGGGGGAGCGGCTTCTCGCCGAGCTGGAGC
Strain LF5HA16 200 ------------------------------------------------------------

Expected sequence 481 GGACTCGGCCTGCGGACGAGGATCTGGCGGTTTGCCACGGTGACCTGTGCCCGGACAACG
Strain LF5HA41 481 GGACTCGGCCTGCGGACGAGGATCTGGCGGTTTGCCACGGTGACCTGTGCCCGGACAACG
Strain LF5HA49 481 GGACTCGGCCTGCGGACGAGGATCTGGCGGTTTGCCACGGTGACCTGTGCCCGGACAACG
Strain LF5HA56 481 GGACTCGGCCTGCGGACGAGGATCTGGCGGTTTGCCACGGTGACCTGTGCCCGGACAACG
Strain LF5HA92 481 GGACTCGGCCTGCGGACGAGGATCTGGCGGTTTGCCACGGTGACCTGTGCCCGGACAACG
Strain LF5HA94 481 GGACTCGGCCTGCGGACGAGGATCTGGCGGTTTGCCACGGTGACCTGTGCCCGGACAACG
Strain LF5HA35 481 GGACTCGGCCTGCGGACGAGGA--------------------------------------
Strain LF5HA16 200 ------------------------------------------------------------

Expected sequence 541 TGCTGCTCGACCCTCGTACCTGCGAGGTGACCGGGCTGATCGACGTGGGGCGGGTCGGCC
Strain LF5HA41 541 TGCTGCTCGACCCTCGTACCTGCGAGGTGACCGGGCTGATCGACGTGGGGCGGGTCGGCC
Strain LF5HA49 541 TGCTGCTCGACCCTCGTACCTGCGAGGTGACCGGGCTGATCGACGTGGGGCGGGTCGGCC
Strain LF5HA56 541 TGCTGCTCGACCCTCGTACCTGCGAGGTGACCGGGCTGATCGACGTGGGGCGGGTCGGCC
Strain LF5HA92 541 TGCTGCTCGACCCTCGTACCTGCGAGGTGACCGGGCTGATCGACGTGGGGCGGGTCGGCC
Strain LF5HA94 541 TGCTGCTCGACCCTCGTACCTGCGAGGTGACCGGGCTGATCGACGTGGGGCGGGTCGGCC
Strain LF5HA35 502 ---------------------------------------------TGGGGCGGCAAGGCC
Strain LF5HA16 200 ------------------------------------------------------------

Expected sequence 600 -GTGCGGACCGGCACTCCGATCTCGCGCTGGTGCTGCGCGAGCTGGCCCACGAGGAGGAC
Strain LF5HA41 600 -GTGCGGACCGGCACTCCGATCTCGCGCTGGTGCTGCGCGAGCTGGCCCACGAGGAGGAC
Strain LF5HA49 600 -GTGCGGACCGGCACTCCGATCTCGCGCTGGTGCTGCGCGAGCTGGCCCACGAGGAGGAC
Strain LF5HA56 600 -GTGCGGACCGGCACTCCGATCTCGCGCTGGTGCTGCGCGAGCTGGCCCACGAGGAGGAC
Strain LF5HA92 600 -GTGCGGACCGGCACTCCGATCTCGCGCTGGTGCTGCGCGAGCTGGCCCACGAGGAGGAC
Strain LF5HA94 600 -GTGCGGACCGGCACTCCGATCTCGCGCTGGTGCTGCGCGAGCTGGCCCACGAGGAGGAC
Strain LF5HA35 518 GGCGCGGGCGGGCA----------GCAGCGGCTCAACGCCAGCCCGCCGCAGATG----C
Strain LF5HA16 200 ------------------------------------------------------------

Expected sequence 660 CCGTGGTTCGGGCCGGAGTGTTCCGCGGCGTTCCTGCGGGAGTACGGGCGCGGGTGGGAT
Strain LF5HA41 660 CCGTGGTTCGGGCCGGAGTGTTCCGCGGCGTTCCTGCGGGAGTACGGGCGCGGGTGGGAT
Strain LF5HA49 660 CCGTGGTTCGGGCCGGAGTGTTCCGCGGCGTTCCTGCGGGAGTACGGGCGCGGGTGGGAT
Strain LF5HA56 660 CCGTGGTTCGGGCCGGAGTGTTCCGCGGCGTTCCTGCGGGAGTACGGGCGCGGGTGGGAT
Strain LF5HA92 660 CCGTGGTTCGGGCCGGAGTGTTCCGCGGCGTTCCTGCGGGAGTACGGGCGCGGGTGGGAT
Strain LF5HA94 660 CCGTGGTTCGGGCCGGAGTGTTCCGCGGCGTTCCTGCGGGAGTACGGGCGCGGGTGGGAT
Strain LF5HA35 564 ACATGG------------------ACGGCGGT-------------GGGTACGCGTCAGGT
Strain LF5HA16 200 ------------------------------------------------------------

Expected sequence 720 GGGGCGGTATCGGAGGAAAAGCTGGCGTTTTACCGGCTGTTGGACGAGTTCTTCTGAGGG
Strain LF5HA41 720 GGGGCGGTATCGGAGGAAAAGCTGGCGTTTTACCGGCTGTTGGACGAGTTCTTCTGAGGG
Strain LF5HA49 720 GGGGCGGTATCGGAGGAAAAGCTGGCGTTTTACCGGCTGTTGGACGAGTTCTTCTGAGGG
Strain LF5HA56 720 GGGGCGGTATCGGAGGAAAAGCTGGCGTTTTACCGGCTGTTGGACGAGTTCTTCTGAGGG
Strain LF5HA92 720 GGGGCGGTATCGGAGGAAAAGCTGGCGTTTTACCGGCTGTTGGACGAGTTCTTCTGAGGG
Strain LF5HA94 720 GGGGCGGTATCGGAGGAAAAGCTGGCGTTTTACCGGCTGTTGGACGAGTTCTTCTGAGGG
Strain LF5HA35 593 G-------------------------------------GCCGCACGAGT-----------
Strain LF5HA16 200 ------------------------------------------------------------

Expected sequence 780 ACCTGATGGTGTTGGTGGCTGGGTAGGGTTGCGTCGCGTGGGTGACAGCACAGTGTGGAC
Strain LF5HA41 780 ACCTGATGGTGTTGGTGGCTGGGTAGGGTTGCGTCGCGTGGGTGACAGCACAGTGTGGAC
Strain LF5HA49 780 ACCTGATGGTGTTGGTGGCTGGGTAGGGTTGCGTCGCGTGGGTGACAGCACAGTGTGGAC
Strain LF5HA56 780 ACCTGATGGTGTTGGTGGCTGGGTAGGGTTGCGTCGCGTGGGTGACAGCACAGTGTGGAC
Strain LF5HA92 780 ACCTGATGGTGTTGGTGGCTGGGTAGGGTTGCGTCGCGTGGGTGACAGCACAGTGTGGAC
Strain LF5HA94 780 ACCTGATGGTGTTGGTGGCTGGGTAGGGTTGCGTCGCGTGGGTGACAGCACAGTGTGGAC
Strain LF5HA35 604 ---------------------------ATCGCGTCGTCTGGG------------------
Strain LF5HA16 200 ------------------------------------------------------------

Expected sequence 840 GTTGGGATCCGGCAAGACTGGCCCCGCTTGGCAACGCAACAGTGAGCCCCTCCCTAGTGT
Strain LF5HA41 840 GTTGGGATCCGGCAAGACTGGCCCCGCTTGGCAACGCAACAGTGAGCCCCTCCCTAGTGT
Strain LF5HA49 840 GTTGGGATCCGGCAAGACTGGCCCCGCTTGGCAACGCAACAGTGAGCCCCTCCCTAGTGT
Strain LF5HA56 840 GTTGGGATCCGGCAAGACTGGCCCCGCTTGGCAACGCAACAGTGAGCCCCTCCCTAGTGT
Strain LF5HA92 840 GTTGGGATCCGGCAAGACTGGCCCCGCTTGGCAACGCAACAGTGAGCCCCTCCCTAGTGT
Strain LF5HA94 840 GTTGGGATCCGGCAAGACTGGCCCCGCTTGGCAACGCAACAGTGAGCCCCTCCCTAGTGT
Strain LF5HA35 619 ------------------------------------------------------------
Strain LF5HA16 200 ------------------------------------------------------------

Expected sequence 900 GTTTGGGGATGTGACTATGTATTCGTGTGTTGGCCAACGGGTCAACCCGAACAGATTGAT
Strain LF5HA41 900 GTTTGGGGATGTGACTATGTATTCGTGTGTTGGCCAACGGGTCAACCCGAACAGATTGAT
Strain LF5HA49 900 GTTTGGGGATGTGACTATGTATTCGTGTGTTGGCCAACGGGTCAACCCGAACAGATTGAT
Strain LF5HA56 900 GTTTGGGGATGTGACTATGTATTCGTGTGTTGGCCAACGGGTCAACCCGAACAGATTGAT
Strain LF5HA92 900 GTTTGGGGATGTGACTATGTATTCGTGTGTTGGCCAACGGGTCAACCCGAACAGATTGAT
Strain LF5HA94 900 GTTTGGGGATGTGACTATGTATTCGTGTGTTGGCCAACGGGTCAACCCGAACAGATTGAT
Strain LF5HA35 619 ------------------------------------------------------------
Strain LF5HA16 200 ------------------------------------------------------------

Expected sequence 960 ACCCGCCTTGGCATTTCCTGTCAGAATGTAACGTCAGTTGATG--GTACCAGC---ATGC
Strain LF5HA41 960 ACCCGCCTTGGCATTTCCTGTCAGAATGTAACGTCAGTTGATG--GTACCAGC--AATGC
Strain LF5HA49 960 ACCCGCCTTGGCATTTCCTGTCAGAATGTAACGTCAGTTGATG--GTACCAGC-------
Strain LF5HA56 960 ACCCGCCTTGGCATTTCCTGTCAGAATGTAACGTCAGTTGATG--GTACCAGC-CGGTGC
Strain LF5HA92 960 ACCCGCCTTGGCATTTCCTGTCAGAATGTAACGTCAGTTGATG--GTACCAGC-------
Strain LF5HA94 960 ACCCGCCTTGGCATTTCCTGTCAGAATGTAACGTCAGTTGATG--GTACCAGC-------
Strain LF5HA35 619 ----------------------------------CAGCCGGTGCTGTACCAGACCAATGC
Strain LF5HA16 200 ---------------------------------------------GTACCAGACCAATGC

Expected sequence 1015 G------GCTGCGGGCGCCAGTAAGCTCAGTCGCGCGCCCAGCCGCGGCGACCCCTGGCA
Strain LF5HA41 1016 G------GCTGCGGGCGCCAGTAAGCTCAGTCGCGCGCCCAGCCGCGGCGACCCCTGGCA
Strain LF5HA49 1011 G------GCTGCGGGCGCCAGTAAGCTCAGTCGCGCGCCCAGCCGCGGCGACCCCTGGCA
Strain LF5HA56 1017 GCCAGCAGCTGCGGGCGCCAGTAAGCTCAGTCGCGCGCCCAGCCGCGGCGACCCCTGGCA
Strain LF5HA92 1010 ------------------------------------------------------------
Strain LF5HA94 1010 ------------------------------------------------------------
Strain LF5HA35 646 C------GCTGCGGGCGCCAGTAAGCTCAGTCGCGCGCCCAGCCGCGGCGACCCCTGGCA
Strain LF5HA16 216 C------GCTGCGGGCGCCAGTAAGCTCAGTCGCGCGCCCAGCCGCGGCGACCCCTGGCA

Expected sequence 1069 ACAGTCGGGCGGTGGGCAGCAAGGGCGCGGCGCGATGCCGCCGCTGCCGCCCGGCGGTGG
Strain LF5HA41 1070 ACAGTCGGGCGGTGGGCAGCAAGGGCGCGGCGCGATGCCGCCGCTGCCGCCCGGCGGTGG
Strain LF5HA49 1065 ACAGTCGGGCGGTGGGCAGCAAGGGCGCGGCGCGATGCCGCCGCTGCCGCCCGGCGGTGG
Strain LF5HA56 1077 ACAGTCGGGCGGTGGGCAGCAAGGGCGCGGCGCGATGCCGCCGCTGCCGCCCGGCGGTGG
Strain LF5HA92 1010 ------------------------------------------------------------
Strain LF5HA94 1010 ----------------------------GGCGCGATGCCGCCGCTGCCGCCCGGCGGTGG
Strain LF5HA35 700 ACAGTCGGGCGGTGGGCAGCAAGGGCGCGGCGCGATGCCGCCGCTGCCGCCCGGCGGTGG
Strain LF5HA16 270 ACAGTCGGGCGGTGGGCAGCAAGGGCGCGGCGCGATGCCGCCGCTGCCGCCCGGCGGTGG

Expected sequence 1129 GCCGCGCATGAGCGGGCACTGGGACGACGACGGCGGCAACCCGGAGCGGCCGTACTCGCG
Strain LF5HA41 1130 GCCGCGCATGAGCGGGCACTGGGACGACGACGGCGGCAACCCGGAGCGGCCGTACTCGCG
Strain LF5HA49 1125 GCCGCGCATGAGCGGGCACTGGGACGACGACGGCGGCAACCCGGAGCGGCCGTACTCGCG
Strain LF5HA56 1137 GCCGCGCATGAGCGGGCACTGGGACGACGACGGCGGCAACCCGGAGCGGCCGTACTCGCG
Strain LF5HA92 1010 ------------------------------------------------------------
Strain LF5HA94 1043 GCCGCGCATGAGCGGGCACTGGGACGACGACGGCGGCAACCCGGAGCGGCCGTACTCGCG
Strain LF5HA35 760 GCCGCGCATGAGCGGGCACTGGGACGACGACGGCGGCAACCCGGAGCGGCCGTACTCGCG
Strain LF5HA16 330 GCCGCGCATGAGCGGGCACTGGGACGACGACGGCGGCAACCCGGAGCGGCCGTACTCGCG

Expected sequence 1189 GGGGATGCTGGGCGGCGGCGGCGGGCCCATGCAGCCGGGCTTTGGGCAGAACCAGATGTG
Strain LF5HA41 1190 GGGGATGCTGGGCGGCGGCGGCGGGCCCATGCAGCCGGGCTTTGGGCAGAACCAGATGTG
Strain LF5HA49 1185 GGGGATGCTGGGCGGCGGCGGCGGGCCCATGCAGCCGGGCTTTGGGCAGAACCAGATGTG
Strain LF5HA56 1197 GGGGATGCTGGGCGGCGGCGGCGGGCCCATGCAGCCGGGCTTTGGGCAGAACCAGATGTG
Strain LF5HA92 1010 ------------------------------------------------------------
Strain LF5HA94 1103 GGGGATGCTGGGCGGCGGCGGCGGGCCCATGCAGCCGGGCTTTGGGCAGAACCAGATGTG
Strain LF5HA35 820 GGGGATGCTGGGCGGCGGCGGCGGGCCCATGCAGCCGGGCTTTGGGCAGAACCAGATGTG
Strain LF5HA16 390 GGGGATGCTGGGCGGCGGCGGCGGGCCCATGCAGCCGGGCTTTGGGCAGAACCAGATGTG

Expected sequence 1249 GCCGCAGCTCAACGTGCAGCAGCAGCAACAGCAGCAGCGGAGGGGCAATTACTA------
Strain LF5HA41 1250 GCCGCAGCTCAACGTGCAGCAGCAGCAACAGCAGCAGCGGAGGGGCAATTACTA------
Strain LF5HA49 1245 GCCGCAGCTCAACGTGCAGCAGCAGCAACAGCAGCAGCGGAGGGGCAATTACTA------
Strain LF5HA56 1257 GCCGCAGCTCAACGTGCAGCAGCAGCAACAGCAGCAGCGGAGGGGCAATTACTA------
Strain LF5HA92 1010 -------------------CAGCAGCAACAGCAGCAGCGGAGGGGCAATTACGGCCTGTC
Strain LF5HA94 1163 GCCGCAGCTCAACGTGCAGCAGCAGCAACAGCAGCAGCGGAGGGGCAATTACGGCCTGTC
Strain LF5HA35 880 GCCGCAGCTCAACGTGCAGCAGCAGCAACAGCAGCAGCGGAGGGGCAATTACGGCCTGTC
Strain LF5HA16 450 GCCGCAGCTCAACGTGCAGCAGCAGCAACAGCAGCAGCGGAGGGGCAATTACGGCCTGTC
 ********

Expected sequence 1302 ------------------------------------------------------------
Strain LF5HA41 1303 ------------------------------------------------------------
Strain LF5HA49 1298 ------------------------------------------------------------
Strain LF5HA56 1310 ------------------------------------------------------------
Strain LF5HA92 1052 GCGATACCCCTACGACGTGCCCGACTACGCCTACCCCTACGACGTGCCCGACTACGCCGA
Strain LF5HA94 1223 GCGATACCCCTACGACGTGCCCGACTACGCCTACCCCTACGACGTGCCCGACTACGCCGA
Strain LF5HA35 940 GCGATACCCCTACGACGTGCCCGACTACGCCTACCCCTACGACGTGCCCGACTACGCCGA
Strain LF5HA16 510 GCGATACCCCTACGACGTGCCCGACTACGCCTACCCCTACGACGTGCCCGACTACGCCGA

************************************************************

Expected sequence 1302 ------------------------------------------GTAGCACAGTGACACTTA
Strain LF5HA41 1303 ------------------------------------------GTAGCACAGTGACACTTA
Strain LF5HA49 1298 ------------------------------------------GTAGCACAGTGACACTTA
Strain LF5HA56 1310 ------------------------------------------GTAGCACAGTGACACTTA
Strain LF5HA92 1112 TCGATCCGGACCGTACCCCTACGACGTGCCCGACTACGCCGCTTAGCACAGTGACACTTA
Strain LF5HA94 1283 TCGATCCGGACCGTACCCCTACGACGTGCCCGACTACGCCGCTTAGCACAGTGACACTTA
Strain LF5HA35 1000 TCGATCCGGACCGTACCCCTACGACGTGCCCGACTACGCCGCTTAGCACAGTGACACTTA
Strain LF5HA16 570 TCGATCCGGACCGTACCCCTACGACGTGCCCGACTACGCCGCTTAGCACAGTGACACTTA

*******************************************

Expected sequence 1321 CTGTGACGGCTTGCTTGCTAGAGGGGCAGCAGCTTCCCTACGCAAGCCCGGTTGTGTGCA
Strain LF5HA41 1322 CTGTGACGGCTTGCTTGCTAGAGGGGCAGCAGCTTCCCTACGCAAGCCCGGTTGTGTGCA
Strain LF5HA49 1317 CTGTGACGGCTTGCTTGCTAGAGGGGCAGCAGCTTCCCTACGCAAGCCCGGTTGTGTGCA
Strain LF5HA56 1329 CTGTGACGGCTTGCTTGCTAGAGGGGCAGCAGCTTCCCTACGCAAGCCCGGTTGTGTGCA
Strain LF5HA92 1172 CTGTGACGGCTTGCTTGCTAGAGGGGCAGCAGCTTCCCTACGCAAGCCCGGTTGTGTGCA
Strain LF5HA94 1343 CTGTGACGGCTTGCTTGCTAGAGGGGCAGCAGCTTCCCTACGCAAGCCCGGTTGTGTGCA
Strain LF5HA35 1060 CTGTGACGGCTTGCTTGCTAGAGGGGCAGCAGCTTCCCTACGCAAGCCCGGTTGTGTGCA
Strain LF5HA16 630 CTGTGACGGCTTGCTTGCTAGAGGGGCAGCAGCTTCCCTACGCAAGCCCGGTTGTGTGCA

Expected sequence 1381 GCGGGGCTGACAATGTCGGCGACAACAAAAGGACTGGACTG
Strain LF5HA41 1382 GCGGGGCTGACAATGTCGGCGACAACAAAAGGACTGGACTG
Strain LF5HA49 1377 GCGGGGCTGACAATGTCGGCGACAACAAAAGGACTGGACTG
Strain LF5HA56 1389 GCGGGGCTGACAATGTCGGCGACAACAAAAGGACTGGACTG
Strain LF5HA92 1232 GCGGGGCTGACAATGTTGGCGACAACAAAAGGACTGGACTG
Strain LF5HA94 1403 GCGGGGCTGACAATGTCGGCGACAACAAAAGGACTGGACTG
Strain LF5HA35 1120 GCGGGGCTGACAATGTCGGCGACAACAAAAGGACTGGACTG
Strain LF5HA16 690 GCGGGGCTGACAATGTCGGCGACAACAAAAGGACTGGACTG

**Part IV: Summary of the sequence analysis:**


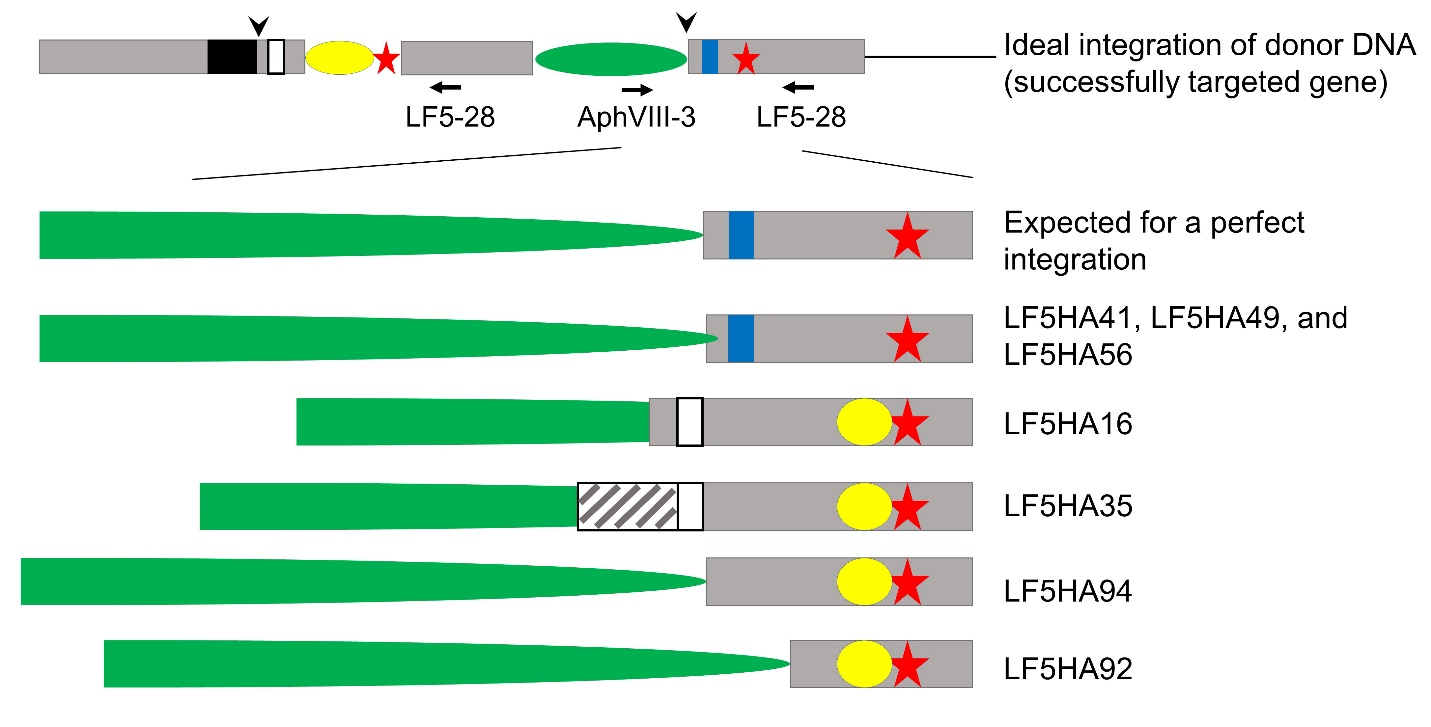


For strains LF5HA41, LF5HA49, and LF5HA56, indel mutations occurred at the integration site. For strains LF5HA16 and LF5HA35, large deletions occurred at the 3’ end of the donor DNA. For LF5HA35, there is an insertion (striped box) of 120 bp from *LF5* exon 10 at the junction site. For LF5HA92 and LF5HA94, large deletions occurred downstream of the integration site. For LF5HA92, LF5HA94, LF5HA35, and LF5HA16, an extra 3HA sequence is present in front of the stop codon, suggesting that multiple copies of the donor DNA have been inserted as a concatemer.

Black box: region corresponding to the left homology arm on donor DNA. Green oval: drug-resistance cassette in the donor DNA. White box: mutated PAM from donor DNA. Blue box: PAM from endogenous gene. Yellow oval: 3HA tag. Red star: stop codon. Grey box: *LF5* gene. Arrowheads: sites of insertion of 5’ and 3’ ends of donor DNA in an ideal integration event. Arrows: Primers used to amplify sequences shown in Parts I and II of this Appendix.
