# Supplementary material for: Direct in situ protein tagging in Chlamydomonas reinhardtii utilizing TIM, a method for CRISPR/Cas9-based targeted insertional mutagenesis
Source: PLoS One. 2022 Dec 9;17(12):e0278972. doi: 10.1371/journal.pone.0278972 (PMC9733891; doi:10.1371/journal.pone.0278972)
Supplement: S3 Appendix — (DOCX) [file pone.0278972.s003.docx]

**S3 Appendix. Sequences of the PCR products for initial screening of NAP1L1-tagged strains (related to Fig 8 of main text)**

Primer pLM160-5: ATGTACGTGTTCCGCAAGAC

Primer NAP1L1-12: CGGCAACAAACTCCTCTGGA

Yellow highlight: PAM or mutated PAM for gRNA2; Red highlight: sequence different from wild-type gene

Sequence of PCR product marked “SD” in Figure 8:

ATGTACGTGTTCCGCAAGACCGAGCTGAAGCACAGCAAGACGGAGCTGAACTTCAAGGAGTGGCAGAAGGCGTTCACCGACGTGATGGGCATGGACGAGCTGTACAAGGGCGGTGGCGACTACAAGGACCATGACGGTGACTATAAGGATCACGACATCGACTACAAGGACGATGACGACAAGGGTGGCGGCATGTCGGGCGACAACGACACACAGCTGATTCAGGCAAGCTAGCTCGCAGGCGTCAAGCGTTCAATAGCCGCCTCGAGCGCCGCCCTGCTCAGTCGCTGGGCTCTGCATCACTTTTAATAAAGTTGTGCCTAAACTGCATTTGCACTAGCGGCTGAGTGCGTGGCGTGGTATTTCAAGCTGAACGTTCAATCGTGAATGTTTCGCAGGCCAAGATGGCGACTCTGGGCCTGGACCAGACTCCAGAGGAGTTTGTTGCCG

Sequence of PCR product marked “SE” in Figure 8:

ATGTACGTGTTCCGCAAGACCGAGCTGAAGCACAGCAAGACGGAGCTGAACTTCAAGGAGTGGCAGAAGGCGTTCACCGACGTGATGGGCATGGACGAGCTGTACAAGGGCGGTGGCGACTACAAGGACCATGACGGTGACTATAAGGATCACGACATCGACTACAAGGACGATGACGACAAGGGTGGCGGCATGTCGGGCGACAACGACACACAGCTGATTCAGGCAAGCTAGCTCGCAGGCGTCAAGCGTTCAATAGCCGCCTCGAGCGCCGCCCTGCTCAGTCGCTGGGCTCTGCATCACTTTTAATAAAGTTGTGCCTAAACTGCATTTGCACTAGCGGCTGAGTGCGTGGCGTGGTATTTCAAGCTGAAGGTTCAATCGTGAATGTTTCGCAGGCCAAGATGGCGACTCTGGGCCTGGACCAGACTCCAGAGGAGTTTGTTGCCG

Sequence of PCR product marked “SX1” in Figure 8:

ATGTACGTGTTCCGCAAGACCGAGCTGAAGCACAGCAAGACGGAGCTGAACTTCAAGGAGTGGCAGAAGGCGTTCACCGACGTGATGGGCATGGACGAGCTGTACAAGGGCGGTGGCGACTACAAGGACCATGACGGTGACTATAAGGATCACGACATCGACTACAAGGACGATGACGACAAGGGTGGCGGCATGTCGGGCGACAACGACACACAGCTGATTCAGGCAAGCTAGCTCGCAGGCGTCAAGCGTTCAATAGCCGCCTCGAGCGCCGCCCTGCTCAGTCGCTGGGCTCTGCATCACTTTTAATAAAGTTGTGCGTAAACTGCATTTGCACTAGCGGCTGAGTGCGTGGCGTGGTATTTCAAGCTGAACGTTCAATCGTGAATGTTTCGCAGGCCAAGATGGCGACTCTGGGCCTGGACCAGACTCCAGAGGAGTTTGTTGCCG

Sequence of PCR product marked “SX2” in Figure 8:

ATGTACGTGTTCCGCAAGACCGAGCTGAAGCACAGCAAGACGGAGCTGAACTTCAAGGAGTGGCAGAAGGCGTTCACCGACGTGATGGGCATGGACGAGCTGTACAAGGGCGGTGGCGACTACAAGGACCATGACGGTGACTATAAGGATCACGACATCGACTACAAGGACGATGACGACAAGGGTGGCGGCATGTCGGGCGACAACGACACACAGCTGATTCAGGCAAGCTAGCTCGCAGGCGTCAAGCGTTCAATAGCCGCCTCGAGCGCCGCCCTGCTCAGTCGCTGGGCTCTGCATCACTTTTAATAAAGTTGTGCCTAAACTGCATTTGCACTAGCGGCTGAGTGCGTGGCGTGGTATTTCAAGCTGAACGTTCAATCGTGAATGTTTCGCGGCCCCGACCCAAGGTTCAATCGTGAATGTTTCGCAGGCCAAGATGGCGACTCTGGGCCTGGACCAGACTCCAGAGGAGTTTGTTGCCG

Sequence of PCR product marked “SX3” in Figure 8:

ATGTACGTGTTCCGCAAGACCGAGCTGAAGCACAGCAAGACGGAGCTGAACTTCAAGGAGTGGCAGAAGGCGTTCACCGACGTGATGGGCATGGACGAGCTGTACAAGGGCGGTGGCGACTACAAGGACCATGACGGTGACTATAAGGATCACGACATCGACTACAAGGACGATGACGACAAGGGTGGCGGCATGTCGGGCGACAACGACACACAGCTGATTCAGGCAAGCTAGCTCGCAGGCGTCAAGCGTTCAATAGCCGCCTCGAGCGCCGCCCTGCTCAGTCGCTGGGCTCTGCATCACGGAAGCTTCTTTCTTGCGCTATGACACTTCCTGAAGGTTCAATCGTGAATGTTTCGCAGGCCAAGATGGCGACTCTGGGCCTGGACCAGACTCCAGAGGAGTTTGTTGCCG

Sequence of PCR product marked “SX4” in Figure 8:

ATGTACGTGTTCCGCAAGACCGAGCTGAAGCACAGCAAGACGGAGCTGAACTTCAAGGAGTGGCAGAAGGCGTTCACCGACGTGATGGGCATGGACGAGCTGTACAAGGGCGGTGGCGACTACAAGGACCATGACGGTGACTATAAGGATCACGACATCGACTACAAGGACGATGACGACAAGGGTGGCGGCATGTCGGGCGACAACGACACACAGCTGATTCAGGCAAGCTAGCTCGCAGGCGTCAAGCGTTCAATAGCCGCCTCGAGCGCCGCCCTGCTCAGTCGCTGGGCTCTGCATCACTTTTAATAAAGTTGTGCCTAAACTGCATTTGCACTAGCGGCTGAGTGCGTGGCGTGGTATTTCAAGCTGAAGGTTCAATCGTGAATGTTTCGCTTGCCAAGATGGCGACTCTGGGCCTGGACCAGACTCCAGAGGAGTTTGTTGCCG

Alignment of all the sequences shown above:

CLUSTAL O(1.2.4) multiple sequence alignment

SX3 ATGTACGTGTTCCGCAAGACCGAGCTGAAGCACAGCAAGACGGAGCTGAACTTCAAGGAG 60

SX2 ATGTACGTGTTCCGCAAGACCGAGCTGAAGCACAGCAAGACGGAGCTGAACTTCAAGGAG 60

SX4 ATGTACGTGTTCCGCAAGACCGAGCTGAAGCACAGCAAGACGGAGCTGAACTTCAAGGAG 60

SX1 ATGTACGTGTTCCGCAAGACCGAGCTGAAGCACAGCAAGACGGAGCTGAACTTCAAGGAG 60

SD ATGTACGTGTTCCGCAAGACCGAGCTGAAGCACAGCAAGACGGAGCTGAACTTCAAGGAG 60

SE ATGTACGTGTTCCGCAAGACCGAGCTGAAGCACAGCAAGACGGAGCTGAACTTCAAGGAG 60

************************************************************

SX3 TGGCAGAAGGCGTTCACCGACGTGATGGGCATGGACGAGCTGTACAAGGGCGGTGGCGAC 120

SX2 TGGCAGAAGGCGTTCACCGACGTGATGGGCATGGACGAGCTGTACAAGGGCGGTGGCGAC 120

SX4 TGGCAGAAGGCGTTCACCGACGTGATGGGCATGGACGAGCTGTACAAGGGCGGTGGCGAC 120

SX1 TGGCAGAAGGCGTTCACCGACGTGATGGGCATGGACGAGCTGTACAAGGGCGGTGGCGAC 120

SD TGGCAGAAGGCGTTCACCGACGTGATGGGCATGGACGAGCTGTACAAGGGCGGTGGCGAC 120

SE TGGCAGAAGGCGTTCACCGACGTGATGGGCATGGACGAGCTGTACAAGGGCGGTGGCGAC 120

************************************************************

SX3 TACAAGGACCATGACGGTGACTATAAGGATCACGACATCGACTACAAGGACGATGACGAC 180

SX2 TACAAGGACCATGACGGTGACTATAAGGATCACGACATCGACTACAAGGACGATGACGAC 180

SX4 TACAAGGACCATGACGGTGACTATAAGGATCACGACATCGACTACAAGGACGATGACGAC 180

SX1 TACAAGGACCATGACGGTGACTATAAGGATCACGACATCGACTACAAGGACGATGACGAC 180

SD TACAAGGACCATGACGGTGACTATAAGGATCACGACATCGACTACAAGGACGATGACGAC 180

SE TACAAGGACCATGACGGTGACTATAAGGATCACGACATCGACTACAAGGACGATGACGAC 180

************************************************************

SX3 AAGGGTGGCGGCATGTCGGGCGACAACGACACACAGCTGATTCAGGCAAGCTAGCTCGCA 240

SX2 AAGGGTGGCGGCATGTCGGGCGACAACGACACACAGCTGATTCAGGCAAGCTAGCTCGCA 240

SX4 AAGGGTGGCGGCATGTCGGGCGACAACGACACACAGCTGATTCAGGCAAGCTAGCTCGCA 240

SX1 AAGGGTGGCGGCATGTCGGGCGACAACGACACACAGCTGATTCAGGCAAGCTAGCTCGCA 240

SD AAGGGTGGCGGCATGTCGGGCGACAACGACACACAGCTGATTCAGGCAAGCTAGCTCGCA 240

SE AAGGGTGGCGGCATGTCGGGCGACAACGACACACAGCTGATTCAGGCAAGCTAGCTCGCA 240

************************************************************

SX3 GGCGTCAAGCGTTCAATAGCCGCCTCGAGCGCCGCCCTGCTCAGTCGCTGGGCTCTGCAT 300

SX2 GGCGTCAAGCGTTCAATAGCCGCCTCGAGCGCCGCCCTGCTCAGTCGCTGGGCTCTGCAT 300

SX4 GGCGTCAAGCGTTCAATAGCCGCCTCGAGCGCCGCCCTGCTCAGTCGCTGGGCTCTGCAT 300

SX1 GGCGTCAAGCGTTCAATAGCCGCCTCGAGCGCCGCCCTGCTCAGTCGCTGGGCTCTGCAT 300

SD GGCGTCAAGCGTTCAATAGCCGCCTCGAGCGCCGCCCTGCTCAGTCGCTGGGCTCTGCAT 300

SE GGCGTCAAGCGTTCAATAGCCGCCTCGAGCGCCGCCCTGCTCAGTCGCTGGGCTCTGCAT 300

************************************************************

SX3 CACGGAAGCTTCTTTCTTGCGCTATGAC-ACTTC-------------------------- 333

SX2 CACTTTTAATAAAGTTGTGCCTAAACTGCATTTGCACTAGCGGCTGAGTGCGTGGCGTGG 360

SX4 CACTTTTAATAAAGTTGTGCCTAAACTGCATTTGCACTAGCGGCTGAGTGCGTGGCGTGG 360

SX1 CACTTTTAATAAAGTTGTGCGTAAACTGCATTTGCACTAGCGGCTGAGTGCGTGGCGTGG 360

SD CACTTTTAATAAAGTTGTGCCTAAACTGCATTTGCACTAGCGGCTGAGTGCGTGGCGTGG 360

SE CACTTTTAATAAAGTTGTGCCTAAACTGCATTTGCACTAGCGGCTGAGTGCGTGGCGTGG 360

*** * * *** *

SX3 ---------CTGAAGGTTCAATCGTGAATGTTTCGCAGGCC------------------- 365

SX2 TATTTCAAGCTGAACGTTCAATCGTGAATGTTTCGCGGCCCCGACCCAAGGTTCAATCGT 420

SX4 TATTTCAAGCTGAAGGTTCAATCGTGAATGTTTCGCTTGCC------------------- 401

SX1 TATTTCAAGCTGAACGTTCAATCGTGAATGTTTCGCAGGCC------------------- 401

SD TATTTCAAGCTGAACGTTCAATCGTGAATGTTTCGCAGGCC------------------- 401

SE TATTTCAAGCTGAAGGTTCAATCGTGAATGTTTCGCAGGCC------------------- 401

*

SX3 ----------------AAGATGGCGACTCTGGGCCTGGACCAGACTCCAGAGGAGTTTGT 409

SX2 GAATGTTTCGCAGGCCAAGATGGCGACTCTGGGCCTGGACCAGACTCCAGAGGAGTTTGT 480

SX4 ----------------AAGATGGCGACTCTGGGCCTGGACCAGACTCCAGAGGAGTTTGT 445

SX1 ----------------AAGATGGCGACTCTGGGCCTGGACCAGACTCCAGAGGAGTTTGT 445

SD ----------------AAGATGGCGACTCTGGGCCTGGACCAGACTCCAGAGGAGTTTGT 445

SE ----------------AAGATGGCGACTCTGGGCCTGGACCAGACTCCAGAGGAGTTTGT 445

********************************************

SX3 TGCCG 414

SX2 TGCCG 485

SX4 TGCCG 450

SX1 TGCCG 450

SD TGCCG 450

SE TGCCG 450

*****
