# Supplementary material for: Direct in situ protein tagging in Chlamydomonas reinhardtii utilizing TIM, a method for CRISPR/Cas9-based targeted insertional mutagenesis
Source: PLoS One. 2022 Dec 9;17(12):e0278972. doi: 10.1371/journal.pone.0278972 (PMC9733891; doi:10.1371/journal.pone.0278972)
Supplement: S4 Appendix — (DOCX) [file pone.0278972.s004.docx]

**S4 Appendix. Sequences of the PCR products amplified from the region surrounding the 5’ end of the inserted donor DNA in NAP1L1-tagged strains (related to Fig 9 of main text)**

Primer NAP1L1-19 (Corresponding to P3 in Figure 10) : GTTGGGCCCTGATAGCATGT

Primer NAP1L1-18 (Corresponding to P4 in Figure 10) : CCAGAATTCCTGGTCGTTCC

Underline: first 17 NTs of gRNA2 sequence

Double underline: gRNA1 sequence

Wavy underline: first 17 NTs of gRNA1 sequence

Grey highlight: Sequence corresponding to hygromycin resistance cassette. Please note that primer NAP1L1-18 is on hygromycin resistance cassette.

Red highlight: point mutation probably caused by PCR error

Expected sequence for perfect insertion at RNP2 cut site:

GTTGGGCCCTGATAGCATGTGATGGCTCGGCGTCTTCGGGTGCTGGGACTGTCCCCTCTGTAACATCCGCATTCATATCTTCCTGCCCAGATGCACCTTGTTATGCTGACTTGCCACGCCGGACAAATTGCGCAACGCTCGGTTGTGCTTATCGCAGCGGAGCCCCCCCTCCCTCCCCCTCGCCCCCCGCGGATCGGTGGACATCAGATGTCCGAGGCCGCACACTGATGGAGGCTGGATGCTGGTGGAGGGACATGGTTGGGAAGTGCAGCGCGCTGACACGGCGGCGTGGTAGCTAGAGGGACATGGCGTCCTTGATTTGTCAAAGTATGGCTCACGCCTCACGGAACCCACGGAGACTCAAGCTCCAATGGGGATCAAATGCCTAACACGTTCAAAGGCTCTTCAAGGACATTACGTGGCTCTATTGGCGATGACCGCACTGTGTCGGTGGAGCGCGCATTTCACGGTGTAGATTATGCCGCGCCCCTTATCAGAAGCGGGTAAAGACACTCTCCTCGTATGTTGGATGCAAACGCAGGTGTGCGCAAGGTGAACAAACCGTCATACAGAAATAGGCGGCATGGAAAACTCGGCCGGAGCGAGAAGCGACCTCGTGAGTCGATGGGTTTACATGGGTACGTGCCGCATCAGACCGGATCCATGCAATATTACTTTGCGTCCAGACCTTATACGCATGCTTTCTTCTTCTAATAGCACAAAGAATGTCGGGCGACAACGACACACAGCTGATTCAGGCAAGCTAGCTCGCAGGCGTCAAGCGTTCAATAGCCGCCTCGAGCGCCGCCCTGCTCAGTCGCTGGGCTCTGCATCACTTTTAATAAAGTTGTGCCTAAACTGCATTTGCACTAGCGGCTGAGTGCGTGGCGTGGTATTTCAAGCCCTTGTTATGCTGACTTGCCAGACCATGATTACGAATTCGATATCAAGCTTCTTTCTTGCGCTATGACACTTCCAGCAAAAGGTAGGGCGGGCTGCGAGACGGCTTCCCGGCGCTGCATGCAACACCGATGATGCTTCGACCCCCCGAAGCTCCTTCGGGGCTGCATGGGCGCTCCGATGCCGCTCCAGGGCGAGCGCTGTTTAAATAGCCAGGCCCCCGATTGCAAAGACATTATAGCGAGCTACCAAAGCCATATTCAAACACCTAGATCACTACCACTTCTACACAGGCCACTCGAGCTTGTGATCGCACTCCGCTAAGGGGGCGCCTCTTCCTCTTCGTTTCAGTCACAACCCGCAAACATGACACAAGAATCCCTGTTACTTCTCGACCGTATTGATTCGGATGATTCCTACGCGAGCCTGCGGAACGACCAGGAATTCTGG

Expected sequence for perfect insertion at RNP1 cut site:

GTTGGGCCCTGATAGCATGTGATGGCTCGGCGTCTTCGGGTGCTGGGACTGTCCCCTCTGTAACATCCGCATTCATATCTTCCTGCCCAGATGCACCTTGTTATGCTGACTTGCCAGACCATGATTACGAATTCGATATCAAGCTTCTTTCTTGCGCTATGACACTTCCAGCAAAAGGTAGGGCGGGCTGCGAGACGGCTTCCCGGCGCTGCATGCAACACCGATGATGCTTCGACCCCCCGAAGCTCCTTCGGGGCTGCATGGGCGCTCCGATGCCGCTCCAGGGCGAGCGCTGTTTAAATAGCCAGGCCCCCGATTGCAAAGACATTATAGCGAGCTACCAAAGCCATATTCAAACACCTAGATCACTACCACTTCTACACAGGCCACTCGAGCTTGTGATCGCACTCCGCTAAGGGGGCGCCTCTTCCTCTTCGTTTCAGTCACAACCCGCAAACATGACACAAGAATCCCTGTTACTTCTCGACCGTATTGATTCGGATGATTCCTACGCGAGCCTGCGGAACGACCAGGAATTCTGG

Sequence of PCR product marked “S1” in Figure 9:

GTTGGGCCCTGATAGCATGTGATGGCTCGGCGTCTTCGGGTGCTGGGACTGTCCCCTCTGTAACATCCGCATTCATATCTTCCTGCCCAGATGCACCTTGTTATGCTGACTTGCCACGCCGGACAAATTGCGCAACGCTCGGTTGTGCTTATCGCAGCGGAGCCCCCCCTCCCTCCCCCTCGCCCCCCGCGGATCGGTGGACATCAGATGTCCGAGGCCGCACACTGATGGAGGCTGGATGCTGGTGGAGGGACATGGTTGGGAAGTGCAGCGCGCTGACACGGCGGCGTGGTAGCTAGAGGGACATGGCGTCCTTGATTTGTCAAAGTATGGCTCACGCCTCACGGAACCCACGGAGACTCAAGCTCCAATGGGGATCAAATGCCTAACACGTTCAAAGGCTCTTCAAGGACATTACGTGGCTCTATTGGCGATGACCGCACTGTGTCGGTGGAGCGCGCATTTCACGGTGTAGATTATGCCGCGCCCCTTATCAGAAGCGGGTAAAGACACTCTCCTCGTATGTTGGATGCAAACGCAGGTGTGCGCAAGGTGAACAAACCGTCATACAGAAATAGGCGGCATGGAAAACTCGGCCGGAGCGAGAAGCGACCTCGTGAGTCGATGGGTTTACATGGGTACGTGCCGCATCAGACCGGATCCATGCAATATTACTTTGCGTCCAGACCTTATACGCATGCTTTCTTCTTCTAATAGCACAAAGAATGTCGGGCGACAACGACACACAGCTGATTCAGGCAAGCTAGCTCGCAGGCGTCAAGCGTTCAATAGCCGCCTCGAGCGCCGCCCTGCTCAGTCGCTGGGCTCTGCATCACTTTTAATAAAGTTGTGCCTAAACTGCATTTGCACTAGCGGCTGAGTGCGTGGCGTGGTATTTCAAGCGCCTTTATGCTGACTTGCCAGACCATGATTACGAATTCGATATCAAGCTTCTTTCTTGCGCTATGACACTTCCAGCAAAAGGTAGGGCGGGCTGCGAGACGGCTTCCCGGCGCTGCATGCAACACCGATGATGCTTCGACCCCCCGAAGCTCCTTCGGGGCTGCATGGGCGCTCCGATGCCGCTCCAGGGCGAGCGCTGTTTAAATAGCCAGGCCCCCGATTGCAAAGACATTATAGCGAGCTACCAAAGCCATATTCAAACACCTAGATCACTACCACTTCTACACAGGCCACTCGAGCTTGTGATCGCACTCCGCTAAGGGGGCGCCTCTTCCTCTTCGTTTCAGTCACAACCCGCAAACATGACACAAGAATCCCTGTTACTTCTCGACCGTATTGATTCGGATGATTCCTACGCGAGCCTGCGGAACGACCAGGAATTCTGG

Sequence of PCR product marked “S2” in Figure 9:

GTTGGGCCCTGATAGCATGTGATGGCTCGGCGTCTTCGGGTGCTGGGACTGTCCCCTCTGTAACATCCGCATTCATATCTTCCTGCCCAGATGCACCTTGTTATGCTGACTTGCCACGCCGGACAAATTGCGCAACGCTCGGTTGTGCTTATCGCAGCGGAGCCCCCCCTCCCTCCCCCTCGCCCCCCGCGGATCGGTGGACATCAGATGTCCGAGGCCGCACACTGATGGAGGCTGGATGCTGGTGGAGGGACATGGTTGGGAAGTGCAGCGCGCTGACACGGCGGCGTGGTAGCTAGAGGGACATGGCGTCCTTGATTTGTCAAAGTATGGCTCACGCCTCACGGAACCCACGGAGACTCAAGCTCCAATGGGGATCAAATGCCTAACACGTTCAAAGGCTCTTCAAGGACATTACGTGGCTCTATTGGCGATGACCGCACTGTGTCGGTGGAGCGCGCATTTCACGGTGTAGATTATGCCGCGCCCCTTATCAGAAGCGGGTAAAGACACTCTCCTCGTATGTTGGATGCAAACGCAGGTGTGCGCAAGGTGAACAAACCGTCATACAGAAATAGGCGGCATGGAAAACTCGGCCGGAGCGAGAAGCGACCTCGTGAGTCGATGGGTTTACATGGGTACGTGCCGCATCAGACCGGATCCATGCAATATTACTTTGCGTCCAGACCTTATACGCATGCTTTCTTCTTCTAATAGCACAAAGAATGTCGGGCGACAACGACACACAGCTGATTCAGGCAAGCTAGCTCGCAGGCGTCAAGCGTTCAATAGCCGCCTCGAGCGCCGCCCTGCTCAGTCGCTGGGCTCTGCATCACTTTTAATAAAGTTGTGCCTAAACTGCATTTGCACTAGCGGCTGAGTGCGTGGCGTGGTATTTCAAGCAAAACATGAGCCCGTTCCAGGCCGCGTTATGCTGACTTGCCAGACCATGATTACGAATTCGATATCAAGCTTCTTTCTTGCGCTATGACACTTCCAGCAAAAGGTAGGGCGGGCTGCGAGACGGCTTCCCGGCGCTGCATGCAACACCGATGATGCTTCGACCCCCCGAAGCTCCTTCGGGGCTGCATGGGCGCTCCGATGCCGCTCCAGGGCGAGCGCTGTTTAAATAGCCAGGCCCCCGATTGCAAAGACATTATAGCGAGCTACCAAAGCCATATTCAAACACCTAGATCACTACCACTTCTACACAGGCCACTCGAGCTTGTGATCGCACTCCGCTAAGGGGGCGCCTCTTCCTCTTCGTTTCAGTCACAACCCGCAAACATGACACAAGAATCCCTGTTACTTCTCGACCGTATTGATTCGGATGATTCCTACGCGAGCCTGCGGAACGACCAGGAATTCTGG

Sequence of PCR product marked “S3” in Figure 9:

GTTGGGCCCTGATAGCATGTGATGGCTCGGCGTCTTCGGGTGCTGGGACTGTCCCCTCTGTAACATCCGCATTCATATCTTCCTGCCCAGATGCACCTTGTTATGCTGACTTGCCACGCCGGACAAATTGCGCAACGCTCGGTTGTGCTTATCGCAGCGGAGCCCCCCCTCCCTCCCCCTCGCCCCCCGCGGATCGGTGGACATCAGATGTCCGAGGCCGCACACTGATGGAGGCTGGATGCTGGTGGAGGGACATGGTTGGGAAGTGCAGCGCGCTGACACGGCGGCGTGGTAGCTAGAGGGACATGGCGTCCTTGATTTGTCAAAGTATGGCTCACGCCTCACGGAACCCACGGAGACTCAAGCTCCAATGGGGATCAAATGCCTAACACGTTCAAAGGCTCTTCAAGGACATTACGTGGCTCTATTGGCGATGACCGCACTGTGTCGGTGGAGCGCGCATTTCACGGTGTAGATTATGCCGCGCCCCTTATCAGAAGCGGGTAAAGACACTCTCCTCGTATGTTGGATGCAAACGCAGGTGTGCGCAAGGTGAACAAACCGTCATACAGAAATAGGCGGCATGGAAAACTCGGCCGGAGCGAGAAGCGACCTCGTGAGTCGATGGGTTTACATGGGTACGTGCCGCATCAGACCGGATCCATGCAATATTACTTTGCGTCCAGACCTTATACGCATGCTTTCTTCTTCTAATAGCACAAAGAATGTCGGGCGACAACGACACACAGCTGATTCAGGCAAGCTAGCTCGCAGGCGTCAAGCGTTCAATAGCCGCCTCGAGCGCCGCCCTGCTCAGTCGCTGGGCTCTGCATCACTTTTAATAAAGTTGTGCCTAAACTGCATTTGCACTAGCGGCTGAGTGCGTGGCGTGGTATTTCAAGCCCGGGCTGCTGACTTGCCAGACCATGATTACGAATTCGATATCAAGCTTCTTTCTTGCGCTATGACACTTCCAGCAAAAGGTAGGGCGGGCTGCGAGACGGCTTCCCGGCGCTGCATGCAACACCGATGATGCTTCGACCCCCCGAAGCTCCTTCGGGGCTGCATGGGCGCTCCGATGCCGCTCCAGGGCGAGCGCTGTTTAAATAGCCAGGCCCCCGATTGCAAAGACATTATAGCGAGCTACCAAAGCCATATTCAAACACCTAGATCACTACCACTTCTACACAGGCCACTCGAGCTTGTGATCGCACTCCGCTAAGGGGGCGCCTCTTCCTCTTCGTTTCAGTCACAACCCGCAAACATGACACAAGAATCCCTGTTACTTCTCGACCGTATTGATTCGGATGATTCCTACGCGAGCCTGCGGAACGACCAGGAATTCTGG

Sequence of PCR product marked “S4” in Figure 9:

GTTGGGCCCTGATAGCATGTGATGGCTCGGCGTCTTCGGGTGCTGGGACTGTCCCCTCTGTAACATCCGCATTCATATCTTCCTGCCCAGATGCACCTTGTTATGCTGACTTGCCACGCCGGACAAATTGCGCAACGCTCGGTTGTGCTTATCGCAGCGGAGCCCCCCCTCCCTCCCCCTCGCCCCCCGCGGATCGGTGGACATCAGATGTCCGAGGCCGCACACTGATGGAGGCTGGATGCTGGTGGAGGGACATGGTTGGGAAGTGCAGCGCGCTGACACGGCGGCGTGGTAGCTAGAGGGACATGGCGTCCTTGATTTGTCAAAGTATGGCTCACGCCTCACGGAACCCACGGAGACTCAAGCTCCAATGGGGATCAAATGCCTAACACGTTCAAAGGCTCTTCAAGGACATTACGTGGCTCTATTGGCGATGACCGCACTGTGTCGGTGGAGCGCGCATTTCACGGTGTAGATTATGCCGCGCCCCTTATCAGAAGCGGGTAAAGACACTCTCCTCGTATGTTGGATGCAAACGCAGGTGTGCGCAAGGTGAACAAACCGTCATACAGAAATAGGCGGCATGGAAAACTCGGCCGGAGCGAGAAGCGACCTCGTGAGTCGATGGGTTTACATGGGTACGTGCCGCATCAGACCGGATCCATGCAATATTACTTTGCGTCCAGACCTTATACGCATGCTTTCTTCTTCTAATAGCACAAAGAATGTCGGGCGACAACGACACACAGCTGATTCAGGCAAGCTAGCTCGCAGGCGTCAAGCGTTCAATAGCCGCCTCGAGCGCCGCCCTGCTCAGTCGCTGGGCTCTGCATCACTTTTAATAAAGTTGTGCCTAAACTGCATTTGCACTAGCGGCTGAGTGCGTGGCGTGGTATTTCAAGCCCCTGCCGCGGCGTGGCGCCCGCAAACATGACACAAGAATCCCTGTTACTTCTCGACCGTATTGATTCGGATGATTCCTACGCGAGCCTGCGGAACGACCAGGAATTCTGG

Sequence of PCR product marked “S5” in Figure 9:

GTTGGGCCCTGATAGCATGTGATGGCTCGGCGTCTTCGGGTGCTGGGACTGTCCCCTCTGTAACATCCGCATTCATATCTTCCTGCCCAGATGCACCTTGTTATGCTGACTTGCCACGCCGGACAAATTGCGCAACGCTCGGTTGTGCTTATCGCAGCGGAGCCCCCCCTCCCTCCCCCTCGCCCCCCGCGGATCGGTGGACATCAGATGTCCGAGGCCGCACACTGATGGAGGCTGGATGCTGGTGGAGGGACATGGTTGGGAAGTGCAGCGCGCTGACACGGCGGCGTGGTAGCTAGAGGGACATGGCGTCCTTGATTTGTCAAAGTATGGCTCACGCCTCACGGAACCCACGGAGACTCAAGCTCCAATGGGGATCAAATGCCTAACACGTTCAAAGGCTCTTCAAGGACATTACGTGGCTCTATTGGCGATGACCGCACTGTGTCGGTGGAGCGCGCATTTCACGGTGTAGATTATGCCGCGCCCCTTATCAGAAGCGGGTAAAGACACTCTCCTCGTATGTTGGATGCAAACGCAGGTGTGCGCAAGGTGAACAAACCGTCATACAGAAATAGGCGGCATGGAAAACTCGGCCGGAGCGAGAAGCGACCTCGTGAGTCGATGGGTTTACATGGGTACGTGCCGCATCAGACCGGATCCATGCAATATTACTTTGCGTCCAGACCTTATACGCATGCTTTCTTCTTCTAATAGCACAAAGAATGTCGGGCGACAACGACACACAGCTGATTCAGGCAAGCTAGCTCGCAGGCGTCAAGCGTTCAATAGCCGCCTCGAGCGCCGCCCTGCTCAGTCGCTGGGCTCTGCATCACTTTTAATAAAGTTGTGCCTAAACTGCATTTGCACTAGCGGCTGAGTGCGTGGCGTGGTATTTCAAGCGGTTCAAGCGGTTTGGGGGCGCCTCTTCCTCTTCGTTTCAGTCACAACCCGCAAACATGACACAAGAATCCCTGTTACTTCTCGACCGTATTGATTCGGATGATTCCTACGCGAGCCTGCGGAACGACCAGGAATTCTGG

Sequence of PCR product marked “S6” in Figure 9:

GTTGGGCCCTGATAGCATGTGATGGCTCGGCGTCTTCGGGTGCTGGGACTGTCCCCTCTGTAACATCCGCATTCATATCTTCCTGCCCAGATGCACCTTGTTATGCTGACTTGCCACGCCGGACAAATTGCGCAACGCTCGGTTGTGCTTATCGCAGCGGAGCCCCCCCTCCCTCCCCCTCGCCCCCCGCGGATCGGTGGACATCAGATGTCCGAGGCCGCACACTGATGGAGGCTGGATGCTGGTGGAGGGACATGGTTGGGAAGTGCAGCGCGCTGACACGGCGGCGTGGTAGCTAGAGGGACATGGCGTCCTTGATTTGTCAAAGTATGGCTCACGCCTCACGGAACCCACGGAGACTCAAGCTCCAATGGGGATCAAATGCCTAACACGTTCAAAGGCTCTTCAAGGACATTACGTGGCTCTATTGGCGATGACCGCACTGTGTCGGTGGAGCGCGCATTTCACGGTGTAGGTTATGCCGCGCCCCTTATCAGAAGCGGGTAAAGACACTCTCCTCGTATGTTGGATGCAAACGCAGGTGTGCGCAAGGTGAACAAACCGTCATACAGAAATAGGCGGCATGGAAAACTCGGCCGGAGCGAGAAGCGACCTCGTGAGTCGATGGGTTTACATGGGTACGTGCCGCATCAGACCGGATCCATGCAATATTACTTTGCGTCCAGACCTTATACGCATGCTTTCTTCTTCTAATAGCACAAAGAATGTCGGGCGACAACGACACACAGCTGATTCAGGCAAGCTAGCTCGCAGGCGTCAAGCGTTCAATAGCCGCCTCGAGCGCCGCCCTGCTCAGTCGCTGGGCTCTGCATCACTTTTAATAAAGTTGTGCCTAAACTGCATTTGCACTAGCGGCTGAGTGCGTGGCGTGGTATTTCAAGCCCGACATGCTGACTTGCCAGACCATGATTACGAATTCGATATCAAGCTTCTTTCTTGCGCTATGACACTTCCAGCAAAAGGTAGGGCGGGCTGCGAGACGGCTTCCCGGCGCTGCATGCAACACCGATGATGCTTCGACCCCCCGAAGCTCCTTCGGGGCTGCATGGGCGCTCCGATGCCGCTCCAGGGCGAGCGCTGTTTAAATAGCCAGGCCCCCGATTGCAAAGACATTATAGCGAGCTACCAAAGCCATATTCAAACACCTAGATCACTACCACTTCTACACAGGCCACTCGAGCTTGTGATCGCACTCCGCTAAGGGGGCGCCTCTTCCTCTTCGTTTCAGTCACAACCCGCAAACATGACACAAGAATCCCTGTTACTTCTCGACCGTATTGATTCGGATGATTCCTACGCGAGCCTGCGGAACGACCAGGAATTCTGG

Sequence of PCR product marked “S7” in Figure 9:

GTTGGGCCCTGATAGCATGTGATGGCTCGGCGTCTTCGGGTGCTGGGACTGTCCCCTCTGTAACATCCGCATTCATATCTTCCTGCCCAGATGCACCTTGTTATGCTGACTTGCCACGCCGGACAAATTGCGCAACGCTCGGTTGTGCTTATCGCAGCGGAGCCCCCCCTCCCTCCCCCTCGCCCCCCGCGGATCGGTGGACATCAGATGTCCGAGGCCGCACACTGATGGAGGCTGGATGCTGGTGGAGGGACATGGTTGGGAAGTGCAGCGCGCTGACACGGCGGCGTGGTAGCTAGAGGGACATGGCGTCCTTGATTTGTCAAAGTATGGCTCACGCCTCACGGAACCCACGGAGACTCAAGCTCCAATGGGGATCAAATGCCTAACACGTTCAAAGGCTCTTCAAGGACATTACGTGGCTCTATTGGCGATGACCGCACTGTGTCGGTGGAGCGCGCATTTCACGGTGTAGATTATGCCGCGCCCCTTATCAGAAGCGGGTAAAGACACTCTCCTCGTATGTTGGATGCAAACGCAGGTGTGCGCAAGGTGAACAAACCGTCATACAGAAATAGGCGGCATGGAAAACTCGGCCGGAGCGAGAAGCGACCTCGTGAGTCGATGGGTTTACATGGGTACGTGCCGCATCAGACCGGATCCATGCAATATTACTTTGCGTCCAGACCTTATACGCATGCTTTCTTCTTCTAATAGCACAAAGAATGTCGGGCGACAACGACACACAGCTGATTCAGGCAAGCTAGCTCGCAGGCGTCAAGCGTTCAATAGCCGCCTCGAGCGCCGCCCTGCTCAGTCGCTGGGCTCTGCATCACTTTTAATAAAGTTGTGCCTAAACTGCATTTGCACTAGCGGCTGAGTGCGTGGCGTGGTATTTCAAGCGCCTTGTTATGCTGACTTGCCAGACCATGATTACGAATTCGATATCAAGCTTCTTTCTTGCGCTATGACACTTCCAGCAAAAGGTAGGGCGGGCTGCGAGACGGCTTCCCGGCGCTGCATGCAACACCGATGATGCTTCGACCCCCCGAAGCTCCTTCGGGGCTGCATGGGCGCTCCGATGCCGCTCCAGGGCGAGCGCTGTTTAAATAGCCAGGCCCCCGATTGCAAAGACATTATAGCGAGCTACCAAAGCCATATTCAAACACCTAGATCACTACCACTTCTACACAGGCCACTCGAGCTTGTGATCGCACTCCGCTAAGGGGGCGCCTCTTCCTCTTCGTTTCAGTCACAACCCGCAAACATGACACAAGAATCCCTGTTACTTCTCGACCGTATTGATTCGGATGATTCCTACGCGAGCCTGCGGAACGACCAGGAATTCTGG

Sequence of PCR product marked “S8” in Figure 9:

GTTGGGCCCTGATAGCATGTGATGGCTCGGCGTCTTCGGGTGCTGGGACTGTCCCCTCTGTAACATCCGCATTCATATCTTCCTGCCCAGATGCACCTTGTTATGCTGACTTGCCACGCCGGACAAATTGCGCAACGCTCGGTTGTGCTTATCGCAGCGGAGCCCCCCCTCCCTCCCCCTCGCCCCCCGCGGATCGGTGGACATCAGATGTCCGAGGCCGCACACTGATGGAGGCTGGATGCTGGTGGAGGGACATGGTTGGGAAGTGCAGCGCGCTGACACGGCGGCGTGGTAGCTAGAGGGACATGGCGTCCTTGATTTGTCAAAGTATGGCTCACGCCTCACGGAACCCACGGAGACTCAAGCTCCAATGGGGATCAAATGCCTAACACGTTCAAAGGCTCTTCAAGGACATTACGTGGCTCTATTGGCGATGACCGCACTGTGTCGGTGGAGCGCGCATTTCACGGTGTAGATTATGCCGCGCCCCTTATCAGAAGCGGGTAAAGACACTCTCCTCGTATGTTGGATGCAAACGCAGGTGTGCGCAAGGTGAACAAACCGTCATACAGAAATAGGCGGCATGGAAAACTCGGCCGGAGCGAGAAGCGACCTCGTGAGTCGATGGGTTTACATGGGTACGTGCCGCATCAGACCGGATCCATGCAATATTACTTTGCGTCCAGACCTTATACGCATGCTTTCTTCTTCTAATAGCACAAAGAATGTCGGGCGACAACGACACACAGCTGATTCAGGCAAGCTAGCTCGCAGGCGTCAAGCGTTCAATAGCCGCCTCGAGCGCCGCCCTGCTCAGTCGCTGGGCTCTGCATCACTTTTAATAAAGTTGTGCCTAAACTGCATTTGCACTAGCGGCTGAGTGCGTGGCGTGGTATTTCAACCCTCCTTGTTATGCTGACTTGCCAGACCATGATTACGAATTCGATATCAAGCTTCTTTCTTGCGCTATGACACTTCCAGCAAAAGGTAGGGCGGGCTGCGAGACGGCTTCCCGGCGCTGCATGCAACACCGATGATGCTTCGACCCCCCGAAGCTCCTTCGGGGCTGCATGGGCGCTCCGATGCCGCTCCAGGGCGAGCGCTGTTTAAATAGCCAGGCCCCCGATTGCAAAGACATTATAGCGAGCTACCAAAGCCATATTCAAACACCTAGATCACTACCACTTCTACACAGGCCACTCGAGCTTGTGATCGCACTCCGCTAAGGGGGCGCCTCTTCCTCTTCGTTTCAGTCACAACCCGCAAACATGACACAAGAATCCCTGTTACTTCTCGACCGTATTGATTCGGATGATTCCTACGCGAGCCTGCGGAACGACCAGGAATTCTGG

Sequence of PCR product marked “S9” in Figure 9:

GTTGGGCCCTGATAGCATGTGATGGCTCGGCGTCTTCGGGTGCTGGGACTGTCCCCTCTGTAACATCCGCATTCATATCTTCCTGCCCAGATGCACCTTGTTATGCTGACTTGCCACGCCGGACAAATTGCGCAACGCTCGGTTGTGCTTATCGCAGCGGAGCCCCCCCTCCCTCCCCCTCGCCCCCCGCGGATCGGTGGACATCAGATGTCCGAGGCCGCACACTGATGGAGGCTGGATGCTGGTGGAGGGACATGGTTGGGAAGTGCAGCGCGCTGACACGGCGGCGTGGTAGCTAGAGGGACATGGCGTCCTTGATTTGTCAAAGTATGGCTCACGCCTCACGGAACCCACGGAGACTCAAGCTCCAATGGGGATCAAATGCCTAACACGTTCAAAGGCTCTTCAAGGACATTACGTGGCTCTATTGGCGATGACCGCACTGTGTCGGTGGAGCGCGCATTTCACGGTGTAGATTATGCCGCGCCCCTTATCAGAAGCGGGTAAAGACACTCTCCTCGTATGTTGGATGCAAACGCAGGTGTGCGCAAGGTGAACAAACCGTCATACAGAAATAGGCGGCATGGAAAACTCGGCCGGAGCGAGAAGCGACCTCGTGAGTCGATGGGTTTACATGGGTACGTGCCGCATCAGACCGGATCCATGCAATATTACTTTGCGTCCAGACCTTATACGCATGCTTTCTTCTTCTAATAGCACAAAGAATGTCGGGCGACAACGACACACAGCTGATTCAGGCAAGCTAGCTCGCAGGCGTCAAGCGTTCAATAGCCGCCTCGAGCGCCGCCCTGCTCAGTCGCTGGGCTCTGCATCACTTTTAATAAAGTTGTGCCTAAACTGCATTTGCACTAGCGGCTGAGTGCGTGGCGTGGTATTTCAAGGGCCCTTGTTATGCTGACTTGCCAGACCATGATTACGAATTCGATATCAAGCTTCTTTCTTGCGCTATGACACTTCCAGCAAAAGGTAGGGCGGGCTGCGAGACGGCTTCCCGGCGCTGCATGCAACACCGATGATGCTTCGACCCCCCGAAGCTCCTTCGGGGCTGCATGGGCGCTCCGATGCCGCTCCAGGGCGAGCGCTGTTTAAATAGCCAGGCCCCCGATTGCAAAGACATTATAGCGAGCTACCAAAGCCATATTCAAACACCTAGATCACTACCACTTCTACACAGGCCACTCGAGCTTGTGATCGCACTCCGCTAAGGGGGCGCCTCTTCCTCTTCGTTTCAGTCACAACCCGCAAACATGACACAAGAATCCCTGTTACTTCTCGACCGTATTGATTCGGATGATTCCTACGCGAGCCTGCGGAACGACCAGGAATTCTGG

Sequence of PCR product marked “S10” in Figure 9:

GTTGGGCCCTGATAGCATGTGATGGCTCGGCGTCTTCGGGTGCTGGGACTGTCCCCTCTGTAACATCCGCATTCATATCTTCCTGCCCAGATGCACCTTGTTATGCTGACTTGCCACGCCGGACAAATTGCGCAACGCTCGGTTGTGCTTATCGCAGCGGAGCCCCCCCTCCCTCCCCCTCGCCCCCCGCGGATCGGTGGACATCAGATGTCCGAGGCCGCACACTGATGGAGGCTGGATGCTGGTGGAGGGACATGGTTGGGAAGTGCAGCGCGCTGACACGGCGGCGTGGTAGCTAGAGGGACATGGCGTCCTTGATTTGTCAAAGTATGGCTCACGCCTCACGGAACCCACGGAGACTCAAGCTCCAATGGGGATCAAATGCCTAACACGTTCAAAGGCTCTTCAAGGACATTACGTGGCTCTATTGGCGATGACCGCACTGTGTCGGTGGAGCGCGCATTTCACGGTGTAGATTATGCCGCGCCCCTTATCAGAAGCGGGTAAAGACACTCTCCTCGTATGTTGGATGCAAACGCAGGTGTGCGCAAGGTGAACAAACCGTCATACAGAAATAGGCGGCATGGAAAACTCGGCCGGAGCGAGAAGCGACCTCGTGAGTCGATGGGTTTACATGGGTACGTGCCGCATCAGACCGGATCCATGCAATATTACTTTGCGTCCAGACCTTATACGCATGCTTTCTTCTTCTAATAGCACAAAGAATGTCGGGCGACAACGACACACAGCTGATTCAGGCAAGCTAGCTCGCAGGCGTCAAGCGTTCAATAGCCGCCTCGAGCGCCGCCCTGCTCAGTCGCTGGGCTCTGCATCACTTTTAATAAAGTTGTGCCTAAACTGCATTTGCACTAGCGGCTGAGTGCGTGGCGTGGTATTTCAAGCGGGCGAAACCGGCATTTGTTATGCTGACTTGCCAGACCATGATTACGAATTCGATATCAAGCTTCTTTCTTGCGCTATGACACTTCCAGCAAAAGGTAGGGCGGGCTGCGAGACGGCTTCCCGGCGCTGCATGCAACACCGATGATGCTTCGACCCCCCGAAGCTCCTTCGGGGCTGCATGGGCGCTCCGATGCCGCTCCAGGGCGAGCGCTGTTTAAATAGCCAGGCCCCCGATTGCAAAGACATTATAGCGAGCTACCAAAGCCATATTCAAACACCTAGATCACTACCACTTCTACACAGGCCACTCGAGCTTGTGATCGCACTCCGCTAAGGGGGCGCCTCTTCCTCTTCGTTTCAGTCACAACCCGCAAACATGACACAAGAATCCCTGTTACTTCTCGACCGTATTGATTCGGATGATTCCTACGCGAGCCTGCGGAACGACCAGGAATTCTGG

Alignment of the sequences of all the PCR products and of the expected sequence for perfect insertion at RNP2 cut site shown above:

CLUSTAL O(1.2.4) multiple sequence alignment

S9 GTTGGGCCCTGATAGCATGTGATGGCTCGGCGTCTTCGGGTGCTGGGACTGTCCCCTCTG 60

S8 GTTGGGCCCTGATAGCATGTGATGGCTCGGCGTCTTCGGGTGCTGGGACTGTCCCCTCTG 60

S7 GTTGGGCCCTGATAGCATGTGATGGCTCGGCGTCTTCGGGTGCTGGGACTGTCCCCTCTG 60

S10 GTTGGGCCCTGATAGCATGTGATGGCTCGGCGTCTTCGGGTGCTGGGACTGTCCCCTCTG 60

S6 GTTGGGCCCTGATAGCATGTGATGGCTCGGCGTCTTCGGGTGCTGGGACTGTCCCCTCTG 60

S1 GTTGGGCCCTGATAGCATGTGATGGCTCGGCGTCTTCGGGTGCTGGGACTGTCCCCTCTG 60

Expected GTTGGGCCCTGATAGCATGTGATGGCTCGGCGTCTTCGGGTGCTGGGACTGTCCCCTCTG 60

S2 GTTGGGCCCTGATAGCATGTGATGGCTCGGCGTCTTCGGGTGCTGGGACTGTCCCCTCTG 60

S3 GTTGGGCCCTGATAGCATGTGATGGCTCGGCGTCTTCGGGTGCTGGGACTGTCCCCTCTG 60

S4 GTTGGGCCCTGATAGCATGTGATGGCTCGGCGTCTTCGGGTGCTGGGACTGTCCCCTCTG 60

S5 GTTGGGCCCTGATAGCATGTGATGGCTCGGCGTCTTCGGGTGCTGGGACTGTCCCCTCTG 60

************************************************************

S9 TAACATCCGCATTCATATCTTCCTGCCCAGATGCACCTTGTTATGCTGACTTGCCACGCC 120

S8 TAACATCCGCATTCATATCTTCCTGCCCAGATGCACCTTGTTATGCTGACTTGCCACGCC 120

S7 TAACATCCGCATTCATATCTTCCTGCCCAGATGCACCTTGTTATGCTGACTTGCCACGCC 120

S10 TAACATCCGCATTCATATCTTCCTGCCCAGATGCACCTTGTTATGCTGACTTGCCACGCC 120

S6 TAACATCCGCATTCATATCTTCCTGCCCAGATGCACCTTGTTATGCTGACTTGCCACGCC 120

S1 TAACATCCGCATTCATATCTTCCTGCCCAGATGCACCTTGTTATGCTGACTTGCCACGCC 120

Expected TAACATCCGCATTCATATCTTCCTGCCCAGATGCACCTTGTTATGCTGACTTGCCACGCC 120

S2 TAACATCCGCATTCATATCTTCCTGCCCAGATGCACCTTGTTATGCTGACTTGCCACGCC 120

S3 TAACATCCGCATTCATATCTTCCTGCCCAGATGCACCTTGTTATGCTGACTTGCCACGCC 120

S4 TAACATCCGCATTCATATCTTCCTGCCCAGATGCACCTTGTTATGCTGACTTGCCACGCC 120

S5 TAACATCCGCATTCATATCTTCCTGCCCAGATGCACCTTGTTATGCTGACTTGCCACGCC 120

************************************************************

S9 GGACAAATTGCGCAACGCTCGGTTGTGCTTATCGCAGCGGAGCCCCCCCTCCCTCCCCCT 180

S8 GGACAAATTGCGCAACGCTCGGTTGTGCTTATCGCAGCGGAGCCCCCCCTCCCTCCCCCT 180

S7 GGACAAATTGCGCAACGCTCGGTTGTGCTTATCGCAGCGGAGCCCCCCCTCCCTCCCCCT 180

S10 GGACAAATTGCGCAACGCTCGGTTGTGCTTATCGCAGCGGAGCCCCCCCTCCCTCCCCCT 180

S6 GGACAAATTGCGCAACGCTCGGTTGTGCTTATCGCAGCGGAGCCCCCCCTCCCTCCCCCT 180

S1 GGACAAATTGCGCAACGCTCGGTTGTGCTTATCGCAGCGGAGCCCCCCCTCCCTCCCCCT 180

Expected GGACAAATTGCGCAACGCTCGGTTGTGCTTATCGCAGCGGAGCCCCCCCTCCCTCCCCCT 180

S2 GGACAAATTGCGCAACGCTCGGTTGTGCTTATCGCAGCGGAGCCCCCCCTCCCTCCCCCT 180

S3 GGACAAATTGCGCAACGCTCGGTTGTGCTTATCGCAGCGGAGCCCCCCCTCCCTCCCCCT 180

S4 GGACAAATTGCGCAACGCTCGGTTGTGCTTATCGCAGCGGAGCCCCCCCTCCCTCCCCCT 180

S5 GGACAAATTGCGCAACGCTCGGTTGTGCTTATCGCAGCGGAGCCCCCCCTCCCTCCCCCT 180

************************************************************

S9 CGCCCCCCGCGGATCGGTGGACATCAGATGTCCGAGGCCGCACACTGATGGAGGCTGGAT 240

S8 CGCCCCCCGCGGATCGGTGGACATCAGATGTCCGAGGCCGCACACTGATGGAGGCTGGAT 240

S7 CGCCCCCCGCGGATCGGTGGACATCAGATGTCCGAGGCCGCACACTGATGGAGGCTGGAT 240

S10 CGCCCCCCGCGGATCGGTGGACATCAGATGTCCGAGGCCGCACACTGATGGAGGCTGGAT 240

S6 CGCCCCCCGCGGATCGGTGGACATCAGATGTCCGAGGCCGCACACTGATGGAGGCTGGAT 240

S1 CGCCCCCCGCGGATCGGTGGACATCAGATGTCCGAGGCCGCACACTGATGGAGGCTGGAT 240

Expected CGCCCCCCGCGGATCGGTGGACATCAGATGTCCGAGGCCGCACACTGATGGAGGCTGGAT 240

S2 CGCCCCCCGCGGATCGGTGGACATCAGATGTCCGAGGCCGCACACTGATGGAGGCTGGAT 240

S3 CGCCCCCCGCGGATCGGTGGACATCAGATGTCCGAGGCCGCACACTGATGGAGGCTGGAT 240

S4 CGCCCCCCGCGGATCGGTGGACATCAGATGTCCGAGGCCGCACACTGATGGAGGCTGGAT 240

S5 CGCCCCCCGCGGATCGGTGGACATCAGATGTCCGAGGCCGCACACTGATGGAGGCTGGAT 240

************************************************************

S9 GCTGGTGGAGGGACATGGTTGGGAAGTGCAGCGCGCTGACACGGCGGCGTGGTAGCTAGA 300

S8 GCTGGTGGAGGGACATGGTTGGGAAGTGCAGCGCGCTGACACGGCGGCGTGGTAGCTAGA 300

S7 GCTGGTGGAGGGACATGGTTGGGAAGTGCAGCGCGCTGACACGGCGGCGTGGTAGCTAGA 300

S10 GCTGGTGGAGGGACATGGTTGGGAAGTGCAGCGCGCTGACACGGCGGCGTGGTAGCTAGA 300

S6 GCTGGTGGAGGGACATGGTTGGGAAGTGCAGCGCGCTGACACGGCGGCGTGGTAGCTAGA 300

S1 GCTGGTGGAGGGACATGGTTGGGAAGTGCAGCGCGCTGACACGGCGGCGTGGTAGCTAGA 300

Expected GCTGGTGGAGGGACATGGTTGGGAAGTGCAGCGCGCTGACACGGCGGCGTGGTAGCTAGA 300

S2 GCTGGTGGAGGGACATGGTTGGGAAGTGCAGCGCGCTGACACGGCGGCGTGGTAGCTAGA 300

S3 GCTGGTGGAGGGACATGGTTGGGAAGTGCAGCGCGCTGACACGGCGGCGTGGTAGCTAGA 300

S4 GCTGGTGGAGGGACATGGTTGGGAAGTGCAGCGCGCTGACACGGCGGCGTGGTAGCTAGA 300

S5 GCTGGTGGAGGGACATGGTTGGGAAGTGCAGCGCGCTGACACGGCGGCGTGGTAGCTAGA 300

************************************************************

S9 GGGACATGGCGTCCTTGATTTGTCAAAGTATGGCTCACGCCTCACGGAACCCACGGAGAC 360

S8 GGGACATGGCGTCCTTGATTTGTCAAAGTATGGCTCACGCCTCACGGAACCCACGGAGAC 360

S7 GGGACATGGCGTCCTTGATTTGTCAAAGTATGGCTCACGCCTCACGGAACCCACGGAGAC 360

S10 GGGACATGGCGTCCTTGATTTGTCAAAGTATGGCTCACGCCTCACGGAACCCACGGAGAC 360

S6 GGGACATGGCGTCCTTGATTTGTCAAAGTATGGCTCACGCCTCACGGAACCCACGGAGAC 360

S1 GGGACATGGCGTCCTTGATTTGTCAAAGTATGGCTCACGCCTCACGGAACCCACGGAGAC 360

Expected GGGACATGGCGTCCTTGATTTGTCAAAGTATGGCTCACGCCTCACGGAACCCACGGAGAC 360

S2 GGGACATGGCGTCCTTGATTTGTCAAAGTATGGCTCACGCCTCACGGAACCCACGGAGAC 360

S3 GGGACATGGCGTCCTTGATTTGTCAAAGTATGGCTCACGCCTCACGGAACCCACGGAGAC 360

S4 GGGACATGGCGTCCTTGATTTGTCAAAGTATGGCTCACGCCTCACGGAACCCACGGAGAC 360

S5 GGGACATGGCGTCCTTGATTTGTCAAAGTATGGCTCACGCCTCACGGAACCCACGGAGAC 360

************************************************************

S9 TCAAGCTCCAATGGGGATCAAATGCCTAACACGTTCAAAGGCTCTTCAAGGACATTACGT 420

S8 TCAAGCTCCAATGGGGATCAAATGCCTAACACGTTCAAAGGCTCTTCAAGGACATTACGT 420

S7 TCAAGCTCCAATGGGGATCAAATGCCTAACACGTTCAAAGGCTCTTCAAGGACATTACGT 420

S10 TCAAGCTCCAATGGGGATCAAATGCCTAACACGTTCAAAGGCTCTTCAAGGACATTACGT 420

S6 TCAAGCTCCAATGGGGATCAAATGCCTAACACGTTCAAAGGCTCTTCAAGGACATTACGT 420

S1 TCAAGCTCCAATGGGGATCAAATGCCTAACACGTTCAAAGGCTCTTCAAGGACATTACGT 420

Expected TCAAGCTCCAATGGGGATCAAATGCCTAACACGTTCAAAGGCTCTTCAAGGACATTACGT 420

S2 TCAAGCTCCAATGGGGATCAAATGCCTAACACGTTCAAAGGCTCTTCAAGGACATTACGT 420

S3 TCAAGCTCCAATGGGGATCAAATGCCTAACACGTTCAAAGGCTCTTCAAGGACATTACGT 420

S4 TCAAGCTCCAATGGGGATCAAATGCCTAACACGTTCAAAGGCTCTTCAAGGACATTACGT 420

S5 TCAAGCTCCAATGGGGATCAAATGCCTAACACGTTCAAAGGCTCTTCAAGGACATTACGT 420

************************************************************

S9 GGCTCTATTGGCGATGACCGCACTGTGTCGGTGGAGCGCGCATTTCACGGTGTAGATTAT 480

S8 GGCTCTATTGGCGATGACCGCACTGTGTCGGTGGAGCGCGCATTTCACGGTGTAGATTAT 480

S7 GGCTCTATTGGCGATGACCGCACTGTGTCGGTGGAGCGCGCATTTCACGGTGTAGATTAT 480

S10 GGCTCTATTGGCGATGACCGCACTGTGTCGGTGGAGCGCGCATTTCACGGTGTAGATTAT 480

S6 GGCTCTATTGGCGATGACCGCACTGTGTCGGTGGAGCGCGCATTTCACGGTGTAGGTTAT 480

S1 GGCTCTATTGGCGATGACCGCACTGTGTCGGTGGAGCGCGCATTTCACGGTGTAGATTAT 480

Expected GGCTCTATTGGCGATGACCGCACTGTGTCGGTGGAGCGCGCATTTCACGGTGTAGATTAT 480

S2 GGCTCTATTGGCGATGACCGCACTGTGTCGGTGGAGCGCGCATTTCACGGTGTAGATTAT 480

S3 GGCTCTATTGGCGATGACCGCACTGTGTCGGTGGAGCGCGCATTTCACGGTGTAGATTAT 480

S4 GGCTCTATTGGCGATGACCGCACTGTGTCGGTGGAGCGCGCATTTCACGGTGTAGATTAT 480

S5 GGCTCTATTGGCGATGACCGCACTGTGTCGGTGGAGCGCGCATTTCACGGTGTAGATTAT 480

*******************************************************.****

S9 GCCGCGCCCCTTATCAGAAGCGGGTAAAGACACTCTCCTCGTATGTTGGATGCAAACGCA 540

S8 GCCGCGCCCCTTATCAGAAGCGGGTAAAGACACTCTCCTCGTATGTTGGATGCAAACGCA 540

S7 GCCGCGCCCCTTATCAGAAGCGGGTAAAGACACTCTCCTCGTATGTTGGATGCAAACGCA 540

S10 GCCGCGCCCCTTATCAGAAGCGGGTAAAGACACTCTCCTCGTATGTTGGATGCAAACGCA 540

S6 GCCGCGCCCCTTATCAGAAGCGGGTAAAGACACTCTCCTCGTATGTTGGATGCAAACGCA 540

S1 GCCGCGCCCCTTATCAGAAGCGGGTAAAGACACTCTCCTCGTATGTTGGATGCAAACGCA 540

Expected GCCGCGCCCCTTATCAGAAGCGGGTAAAGACACTCTCCTCGTATGTTGGATGCAAACGCA 540

S2 GCCGCGCCCCTTATCAGAAGCGGGTAAAGACACTCTCCTCGTATGTTGGATGCAAACGCA 540

S3 GCCGCGCCCCTTATCAGAAGCGGGTAAAGACACTCTCCTCGTATGTTGGATGCAAACGCA 540

S4 GCCGCGCCCCTTATCAGAAGCGGGTAAAGACACTCTCCTCGTATGTTGGATGCAAACGCA 540

S5 GCCGCGCCCCTTATCAGAAGCGGGTAAAGACACTCTCCTCGTATGTTGGATGCAAACGCA 540

************************************************************

S9 GGTGTGCGCAAGGTGAACAAACCGTCATACAGAAATAGGCGGCATGGAAAACTCGGCCGG 600

S8 GGTGTGCGCAAGGTGAACAAACCGTCATACAGAAATAGGCGGCATGGAAAACTCGGCCGG 600

S7 GGTGTGCGCAAGGTGAACAAACCGTCATACAGAAATAGGCGGCATGGAAAACTCGGCCGG 600

S10 GGTGTGCGCAAGGTGAACAAACCGTCATACAGAAATAGGCGGCATGGAAAACTCGGCCGG 600

S6 GGTGTGCGCAAGGTGAACAAACCGTCATACAGAAATAGGCGGCATGGAAAACTCGGCCGG 600

S1 GGTGTGCGCAAGGTGAACAAACCGTCATACAGAAATAGGCGGCATGGAAAACTCGGCCGG 600

Expected GGTGTGCGCAAGGTGAACAAACCGTCATACAGAAATAGGCGGCATGGAAAACTCGGCCGG 600

S2 GGTGTGCGCAAGGTGAACAAACCGTCATACAGAAATAGGCGGCATGGAAAACTCGGCCGG 600

S3 GGTGTGCGCAAGGTGAACAAACCGTCATACAGAAATAGGCGGCATGGAAAACTCGGCCGG 600

S4 GGTGTGCGCAAGGTGAACAAACCGTCATACAGAAATAGGCGGCATGGAAAACTCGGCCGG 600

S5 GGTGTGCGCAAGGTGAACAAACCGTCATACAGAAATAGGCGGCATGGAAAACTCGGCCGG 600

************************************************************

S9 AGCGAGAAGCGACCTCGTGAGTCGATGGGTTTACATGGGTACGTGCCGCATCAGACCGGA 660

S8 AGCGAGAAGCGACCTCGTGAGTCGATGGGTTTACATGGGTACGTGCCGCATCAGACCGGA 660

S7 AGCGAGAAGCGACCTCGTGAGTCGATGGGTTTACATGGGTACGTGCCGCATCAGACCGGA 660

S10 AGCGAGAAGCGACCTCGTGAGTCGATGGGTTTACATGGGTACGTGCCGCATCAGACCGGA 660

S6 AGCGAGAAGCGACCTCGTGAGTCGATGGGTTTACATGGGTACGTGCCGCATCAGACCGGA 660

S1 AGCGAGAAGCGACCTCGTGAGTCGATGGGTTTACATGGGTACGTGCCGCATCAGACCGGA 660

Expected AGCGAGAAGCGACCTCGTGAGTCGATGGGTTTACATGGGTACGTGCCGCATCAGACCGGA 660

S2 AGCGAGAAGCGACCTCGTGAGTCGATGGGTTTACATGGGTACGTGCCGCATCAGACCGGA 660

S3 AGCGAGAAGCGACCTCGTGAGTCGATGGGTTTACATGGGTACGTGCCGCATCAGACCGGA 660

S4 AGCGAGAAGCGACCTCGTGAGTCGATGGGTTTACATGGGTACGTGCCGCATCAGACCGGA 660

S5 AGCGAGAAGCGACCTCGTGAGTCGATGGGTTTACATGGGTACGTGCCGCATCAGACCGGA 660

************************************************************

S9 TCCATGCAATATTACTTTGCGTCCAGACCTTATACGCATGCTTTCTTCTTCTAATAGCAC 720

S8 TCCATGCAATATTACTTTGCGTCCAGACCTTATACGCATGCTTTCTTCTTCTAATAGCAC 720

S7 TCCATGCAATATTACTTTGCGTCCAGACCTTATACGCATGCTTTCTTCTTCTAATAGCAC 720

S10 TCCATGCAATATTACTTTGCGTCCAGACCTTATACGCATGCTTTCTTCTTCTAATAGCAC 720

S6 TCCATGCAATATTACTTTGCGTCCAGACCTTATACGCATGCTTTCTTCTTCTAATAGCAC 720

S1 TCCATGCAATATTACTTTGCGTCCAGACCTTATACGCATGCTTTCTTCTTCTAATAGCAC 720

Expected TCCATGCAATATTACTTTGCGTCCAGACCTTATACGCATGCTTTCTTCTTCTAATAGCAC 720

S2 TCCATGCAATATTACTTTGCGTCCAGACCTTATACGCATGCTTTCTTCTTCTAATAGCAC 720

S3 TCCATGCAATATTACTTTGCGTCCAGACCTTATACGCATGCTTTCTTCTTCTAATAGCAC 720

S4 TCCATGCAATATTACTTTGCGTCCAGACCTTATACGCATGCTTTCTTCTTCTAATAGCAC 720

S5 TCCATGCAATATTACTTTGCGTCCAGACCTTATACGCATGCTTTCTTCTTCTAATAGCAC 720

************************************************************

S9 AAAGAATGTCGGGCGACAACGACACACAGCTGATTCAGGCAAGCTAGCTCGCAGGCGTCA 780

S8 AAAGAATGTCGGGCGACAACGACACACAGCTGATTCAGGCAAGCTAGCTCGCAGGCGTCA 780

S7 AAAGAATGTCGGGCGACAACGACACACAGCTGATTCAGGCAAGCTAGCTCGCAGGCGTCA 780

S10 AAAGAATGTCGGGCGACAACGACACACAGCTGATTCAGGCAAGCTAGCTCGCAGGCGTCA 780

S6 AAAGAATGTCGGGCGACAACGACACACAGCTGATTCAGGCAAGCTAGCTCGCAGGCGTCA 780

S1 AAAGAATGTCGGGCGACAACGACACACAGCTGATTCAGGCAAGCTAGCTCGCAGGCGTCA 780

Expected AAAGAATGTCGGGCGACAACGACACACAGCTGATTCAGGCAAGCTAGCTCGCAGGCGTCA 780

S2 AAAGAATGTCGGGCGACAACGACACACAGCTGATTCAGGCAAGCTAGCTCGCAGGCGTCA 780

S3 AAAGAATGTCGGGCGACAACGACACACAGCTGATTCAGGCAAGCTAGCTCGCAGGCGTCA 780

S4 AAAGAATGTCGGGCGACAACGACACACAGCTGATTCAGGCAAGCTAGCTCGCAGGCGTCA 780

S5 AAAGAATGTCGGGCGACAACGACACACAGCTGATTCAGGCAAGCTAGCTCGCAGGCGTCA 780

************************************************************

S9 AGCGTTCAATAGCCGCCTCGAGCGCCGCCCTGCTCAGTCGCTGGGCTCTGCATCACTTTT 840

S8 AGCGTTCAATAGCCGCCTCGAGCGCCGCCCTGCTCAGTCGCTGGGCTCTGCATCACTTTT 840

S7 AGCGTTCAATAGCCGCCTCGAGCGCCGCCCTGCTCAGTCGCTGGGCTCTGCATCACTTTT 840

S10 AGCGTTCAATAGCCGCCTCGAGCGCCGCCCTGCTCAGTCGCTGGGCTCTGCATCACTTTT 840

S6 AGCGTTCAATAGCCGCCTCGAGCGCCGCCCTGCTCAGTCGCTGGGCTCTGCATCACTTTT 840

S1 AGCGTTCAATAGCCGCCTCGAGCGCCGCCCTGCTCAGTCGCTGGGCTCTGCATCACTTTT 840

Expected AGCGTTCAATAGCCGCCTCGAGCGCCGCCCTGCTCAGTCGCTGGGCTCTGCATCACTTTT 840

S2 AGCGTTCAATAGCCGCCTCGAGCGCCGCCCTGCTCAGTCGCTGGGCTCTGCATCACTTTT 840

S3 AGCGTTCAATAGCCGCCTCGAGCGCCGCCCTGCTCAGTCGCTGGGCTCTGCATCACTTTT 840

S4 AGCGTTCAATAGCCGCCTCGAGCGCCGCCCTGCTCAGTCGCTGGGCTCTGCATCACTTTT 840

S5 AGCGTTCAATAGCCGCCTCGAGCGCCGCCCTGCTCAGTCGCTGGGCTCTGCATCACTTTT 840

************************************************************

S9 AATAAAGTTGTGCCTAAACTGCATTTGCACTAGCGGCTGAGTGCGTGGCGTGGTATTTCA 900

S8 AATAAAGTTGTGCCTAAACTGCATTTGCACTAGCGGCTGAGTGCGTGGCGTGGTATTTCA 900

S7 AATAAAGTTGTGCCTAAACTGCATTTGCACTAGCGGCTGAGTGCGTGGCGTGGTATTTCA 900

S10 AATAAAGTTGTGCCTAAACTGCATTTGCACTAGCGGCTGAGTGCGTGGCGTGGTATTTCA 900

S6 AATAAAGTTGTGCCTAAACTGCATTTGCACTAGCGGCTGAGTGCGTGGCGTGGTATTTCA 900

S1 AATAAAGTTGTGCCTAAACTGCATTTGCACTAGCGGCTGAGTGCGTGGCGTGGTATTTCA 900

Expected AATAAAGTTGTGCCTAAACTGCATTTGCACTAGCGGCTGAGTGCGTGGCGTGGTATTTCA 900

S2 AATAAAGTTGTGCCTAAACTGCATTTGCACTAGCGGCTGAGTGCGTGGCGTGGTATTTCA 900

S3 AATAAAGTTGTGCCTAAACTGCATTTGCACTAGCGGCTGAGTGCGTGGCGTGGTATTTCA 900

S4 AATAAAGTTGTGCCTAAACTGCATTTGCACTAGCGGCTGAGTGCGTGGCGTGGTATTTCA 900

S5 AATAAAGTTGTGCCTAAACTGCATTTGCACTAGCGGCTGAGTGCGTGGCGTGGTATTTCA 900

************************************************************

S9 AGG-------------------GCCCTTGTTATGCTGACTTGCCAGACCATGATTACGAA 941

S8 ACCC-------------------TCCTTGTTATGCTGACTTGCCAGACCATGATTACGAA 941

S7 AGCG--------------------CCTTGTTATGCTGACTTGCCAGACCATGATTACGAA 940

S10 AGCGGGCGA--------AACCGGCATTTGTTATGCTGACTTGCCAGACCATGATTACGAA 952

S6 AGC-----------------------CCGACATGCTGACTTGCCAGACCATGATTACGAA 937

S1 AGCG----------------------CCTTTATGCTGACTTGCCAGACCATGATTACGAA 938

Expected AGC---------------------CCTTGTTATGCTGACTTGCCAGACCATGATTACGAA 939

S2 AGCAAAACATGAGCCCGTTCCAGGCCGCGTTATGCTGACTTGCCAGACCATGATTACGAA 960

S3 AGCC-----------------------CGGGCTGCTGACTTGCCAGACCATGATTACGAA 937

S4 AGCC-----------------------CCTGCC--------------------------- 910

S5 AGCG-----------------------GTTCAA--------------------------- 910

* .

S9 TTCGATATCAAGCTTCTTTCTTGCGCTATGACACTTCCAGCAAAAGGTAGGGCGGGCTGC 1001

S8 TTCGATATCAAGCTTCTTTCTTGCGCTATGACACTTCCAGCAAAAGGTAGGGCGGGCTGC 1001

S7 TTCGATATCAAGCTTCTTTCTTGCGCTATGACACTTCCAGCAAAAGGTAGGGCGGGCTGC 1000

S10 TTCGATATCAAGCTTCTTTCTTGCGCTATGACACTTCCAGCAAAAGGTAGGGCGGGCTGC 1012

S6 TTCGATATCAAGCTTCTTTCTTGCGCTATGACACTTCCAGCAAAAGGTAGGGCGGGCTGC 997

S1 TTCGATATCAAGCTTCTTTCTTGCGCTATGACACTTCCAGCAAAAGGTAGGGCGGGCTGC 998

Expected TTCGATATCAAGCTTCTTTCTTGCGCTATGACACTTCCAGCAAAAGGTAGGGCGGGCTGC 999

S2 TTCGATATCAAGCTTCTTTCTTGCGCTATGACACTTCCAGCAAAAGGTAGGGCGGGCTGC 1020

S3 TTCGATATCAAGCTTCTTTCTTGCGCTATGACACTTCCAGCAAAAGGTAGGGCGGGCTGC 997

S4 ------------------------------------------------------------ 910

S5 ------------------------------------------------------------ 910

S9 GAGACGGCTTCCCGGCGCTGCATGCAACACCGATGATGCTTCGACCCCCCGAAGCTCCTT 1061

S8 GAGACGGCTTCCCGGCGCTGCATGCAACACCGATGATGCTTCGACCCCCCGAAGCTCCTT 1061

S7 GAGACGGCTTCCCGGCGCTGCATGCAACACCGATGATGCTTCGACCCCCCGAAGCTCCTT 1060

S10 GAGACGGCTTCCCGGCGCTGCATGCAACACCGATGATGCTTCGACCCCCCGAAGCTCCTT 1072

S6 GAGACGGCTTCCCGGCGCTGCATGCAACACCGATGATGCTTCGACCCCCCGAAGCTCCTT 1057

S1 GAGACGGCTTCCCGGCGCTGCATGCAACACCGATGATGCTTCGACCCCCCGAAGCTCCTT 1058

Expected GAGACGGCTTCCCGGCGCTGCATGCAACACCGATGATGCTTCGACCCCCCGAAGCTCCTT 1059

S2 GAGACGGCTTCCCGGCGCTGCATGCAACACCGATGATGCTTCGACCCCCCGAAGCTCCTT 1080

S3 GAGACGGCTTCCCGGCGCTGCATGCAACACCGATGATGCTTCGACCCCCCGAAGCTCCTT 1057

S4 ------------------------------------------------------------ 910

S5 ------------------------------------------------------------ 910

S9 CGGGGCTGCATGGGCGCTCCGATGCCGCTCCAGGGCGAGCGCTGTTTAAATAGCCAGGCC 1121

S8 CGGGGCTGCATGGGCGCTCCGATGCCGCTCCAGGGCGAGCGCTGTTTAAATAGCCAGGCC 1121

S7 CGGGGCTGCATGGGCGCTCCGATGCCGCTCCAGGGCGAGCGCTGTTTAAATAGCCAGGCC 1120

S10 CGGGGCTGCATGGGCGCTCCGATGCCGCTCCAGGGCGAGCGCTGTTTAAATAGCCAGGCC 1132

S6 CGGGGCTGCATGGGCGCTCCGATGCCGCTCCAGGGCGAGCGCTGTTTAAATAGCCAGGCC 1117

S1 CGGGGCTGCATGGGCGCTCCGATGCCGCTCCAGGGCGAGCGCTGTTTAAATAGCCAGGCC 1118

Expected CGGGGCTGCATGGGCGCTCCGATGCCGCTCCAGGGCGAGCGCTGTTTAAATAGCCAGGCC 1119

S2 CGGGGCTGCATGGGCGCTCCGATGCCGCTCCAGGGCGAGCGCTGTTTAAATAGCCAGGCC 1140

S3 CGGGGCTGCATGGGCGCTCCGATGCCGCTCCAGGGCGAGCGCTGTTTAAATAGCCAGGCC 1117

S4 ------------------------------------------------------------ 910

S5 ------------------------------------------------------------ 910

S9 CCCGATTGCAAAGACATTATAGCGAGCTACCAAAGCCATATTCAAACACCTAGATCACTA 1181

S8 CCCGATTGCAAAGACATTATAGCGAGCTACCAAAGCCATATTCAAACACCTAGATCACTA 1181

S7 CCCGATTGCAAAGACATTATAGCGAGCTACCAAAGCCATATTCAAACACCTAGATCACTA 1180

S10 CCCGATTGCAAAGACATTATAGCGAGCTACCAAAGCCATATTCAAACACCTAGATCACTA 1192

S6 CCCGATTGCAAAGACATTATAGCGAGCTACCAAAGCCATATTCAAACACCTAGATCACTA 1177

S1 CCCGATTGCAAAGACATTATAGCGAGCTACCAAAGCCATATTCAAACACCTAGATCACTA 1178

Expected CCCGATTGCAAAGACATTATAGCGAGCTACCAAAGCCATATTCAAACACCTAGATCACTA 1179

S2 CCCGATTGCAAAGACATTATAGCGAGCTACCAAAGCCATATTCAAACACCTAGATCACTA 1200

S3 CCCGATTGCAAAGACATTATAGCGAGCTACCAAAGCCATATTCAAACACCTAGATCACTA 1177

S4 ------------------------------------------------------------ 910

S5 ------------------------------------------------------------ 910

S9 CCACTTCTACACAGGCCACTCGAGCTTGTGATCGCACTCCGCTAAGGGGGCGCCTCTTCC 1241

S8 CCACTTCTACACAGGCCACTCGAGCTTGTGATCGCACTCCGCTAAGGGGGCGCCTCTTCC 1241

S7 CCACTTCTACACAGGCCACTCGAGCTTGTGATCGCACTCCGCTAAGGGGGCGCCTCTTCC 1240

S10 CCACTTCTACACAGGCCACTCGAGCTTGTGATCGCACTCCGCTAAGGGGGCGCCTCTTCC 1252

S6 CCACTTCTACACAGGCCACTCGAGCTTGTGATCGCACTCCGCTAAGGGGGCGCCTCTTCC 1237

S1 CCACTTCTACACAGGCCACTCGAGCTTGTGATCGCACTCCGCTAAGGGGGCGCCTCTTCC 1238

Expected CCACTTCTACACAGGCCACTCGAGCTTGTGATCGCACTCCGCTAAGGGGGCGCCTCTTCC 1239

S2 CCACTTCTACACAGGCCACTCGAGCTTGTGATCGCACTCCGCTAAGGGGGCGCCTCTTCC 1260

S3 CCACTTCTACACAGGCCACTCGAGCTTGTGATCGCACTCCGCTAAGGGGGCGCCTCTTCC 1237

S4 --------------------------------------GCGG------------------ 914

S5 --------------------------------------GCGGTTTGGGGGCGCCTCTTCC 932

**

S9 TCTTCGTTTCAGTCACAACCCGCAAACATGACACAAGAATCCCTGTTACTTCTCGACCGT 1301

S8 TCTTCGTTTCAGTCACAACCCGCAAACATGACACAAGAATCCCTGTTACTTCTCGACCGT 1301

S7 TCTTCGTTTCAGTCACAACCCGCAAACATGACACAAGAATCCCTGTTACTTCTCGACCGT 1300

S10 TCTTCGTTTCAGTCACAACCCGCAAACATGACACAAGAATCCCTGTTACTTCTCGACCGT 1312

S6 TCTTCGTTTCAGTCACAACCCGCAAACATGACACAAGAATCCCTGTTACTTCTCGACCGT 1297

S1 TCTTCGTTTCAGTCACAACCCGCAAACATGACACAAGAATCCCTGTTACTTCTCGACCGT 1298

Expected TCTTCGTTTCAGTCACAACCCGCAAACATGACACAAGAATCCCTGTTACTTCTCGACCGT 1299

S2 TCTTCGTTTCAGTCACAACCCGCAAACATGACACAAGAATCCCTGTTACTTCTCGACCGT 1320

S3 TCTTCGTTTCAGTCACAACCCGCAAACATGACACAAGAATCCCTGTTACTTCTCGACCGT 1297

S4 ----CGTG-------GCGCCCGCAAACATGACACAAGAATCCCTGTTACTTCTCGACCGT 963

S5 TCTTCGTTTCAGTCACAACCCGCAAACATGACACAAGAATCCCTGTTACTTCTCGACCGT 992

*** ..******************************************

S9 ATTGATTCGGATGATTCCTACGCGAGCCTGCGGAACGACCAGGAATTCTGG 1352

S8 ATTGATTCGGATGATTCCTACGCGAGCCTGCGGAACGACCAGGAATTCTGG 1352

S7 ATTGATTCGGATGATTCCTACGCGAGCCTGCGGAACGACCAGGAATTCTGG 1351

S10 ATTGATTCGGATGATTCCTACGCGAGCCTGCGGAACGACCAGGAATTCTGG 1363

S6 ATTGATTCGGATGATTCCTACGCGAGCCTGCGGAACGACCAGGAATTCTGG 1348

S1 ATTGATTCGGATGATTCCTACGCGAGCCTGCGGAACGACCAGGAATTCTGG 1349

Expected ATTGATTCGGATGATTCCTACGCGAGCCTGCGGAACGACCAGGAATTCTGG 1350

S2 ATTGATTCGGATGATTCCTACGCGAGCCTGCGGAACGACCAGGAATTCTGG 1371

S3 ATTGATTCGGATGATTCCTACGCGAGCCTGCGGAACGACCAGGAATTCTGG 1348

S4 ATTGATTCGGATGATTCCTACGCGAGCCTGCGGAACGACCAGGAATTCTGG 1014

S5 ATTGATTCGGATGATTCCTACGCGAGCCTGCGGAACGACCAGGAATTCTGG 1043

***************************************************
