# Supplementary material for: Direct in situ protein tagging in Chlamydomonas reinhardtii utilizing TIM, a method for CRISPR/Cas9-based targeted insertional mutagenesis
Source: PLoS One. 2022 Dec 9;17(12):e0278972. doi: 10.1371/journal.pone.0278972 (PMC9733891; doi:10.1371/journal.pone.0278972)
Supplement: S5 Appendix — (DOCX) [file pone.0278972.s005.docx]

**S5 Appendix: Plasmid sequences**

pLF5CsfGFP:

CTGACGCGCCCTGTAGCGGCGCATTAAGCGCGGCGGGTGTGGTGGTTACGCGCAGCGTGACCGCTACACTTGCCAGCGCCCTAGCGCCCGCTCCTTTCGCTTTCTTCCCTTCCTTTCTCGCCACGTTCGCCGGCTTTCCCCGTCAAGCTCTAAATCGGGGGCTCCCTTTAGGGTTCCGATTTAGTGCTTTACGGCACCTCGACCCCAAAAAACTTGATTAGGGTGATGGTTCACGTAGTGGGCCATCGCCCTGATAGACGGTTTTTCGCCCTTTGACGTTGGAGTCCACGTTCTTTAATAGTGGACTCTTGTTCCAAACTGGAACAACACTCAACCCTATCTCGGTCTATTCTTTTGATTTAAATTCATAACTTCGTATAATGTATGCTATACGAAGTTATGAATTAGGGATTTTGCCGATTTCGGCCTATTGGTTAAAAAATGAGCTGATTTAACAAAAATTTAACGCGAATTTTAACAAAATATTAACGCTTACAATTTCCATTCGCCATTCAGGCTGCGCAACTGTTGGGAAGGGCGATCGGTGCGGGCCTCTTCGCTATTACGCCAGCTGGCGAAAGGGGGATGTGCTGCAAGGCGATTAAGTTGGGTAACGCCAGGGTTTTCCCAGTCACGACGTTGTAAAACGACGGCCAGTGAattGTAATACGACTCACTATAGGGCGAATTGGAGCTGACTGCTCGTGAATTCAAGGCGGTTGGCGGTAAGGTCACGTACTGGCCTCGCCGCCAAAAGTTCTGCGCAAGGCAGGAGGTTTTGCCACCTTGATTGAATCCATCGTTCACCCGTCCACGGTCACGGCCATGCATGCCGACATTTGCGGCCATGGCTGTTTGGGGTGCCAAGTGGTGCTTTGAGCGCTTGCTTTCTGGCTCATGACTTATGATGCGTTTCCTATCAGTTCCATGGCGGCACGAAGCTCTTGTTGAAATACGTGCCACCTGGCAGCGCGCGGCGGTCGTGTCTCCACGCGCCGCGCGCGGGCGGAAGGGTGGAAGTTTTATGCTCGACATGATTCGTTGTGCCCGAGTGAGTACAATACTATGCAGTGCAGCACACGTCGCTATATGGGTTTGTCAGGGCTTTGCTGCAGCGAGGCCTGCTGCAGCTGGGAACCGAATGAGCCGGGCGTCACCGATGTGGAAACGCAGTGTTCCTCGAGTCGCGAGCTTCGCGTGCCTTTGACTTGCAAAATTCACATGCCAACAACGTAGTATCGACTGATACAGCACTACATAAAGAGTCCTGGGCGAAAACGTTCACATCCATTTCGTCACCGCGGACACATATCGACTATCTTAAACCGATAATCTGAATTTCTACTTCTCAATTAGGCGCTTAGCCCATGACTGGGCTGACCTAGCGCGACCGCGGTCGCTTCGAAGGGCAGCCAAAGACTAGCACCCGAAGCCGCGTAGGGAGCCAGCAAGCCTTCGGGAATCGTAGACATCTTAATACAGCTTCTGTTATTGTGGGCAGCGGCGCGGGGAGGGCGCTGGAGCTCCGCTCGTGCTAGCTGGGCTCCCAGCAGCGCATGAGATGGTGCGTATTGGGGTCGATATTGCCAAGAACTCTTGCAACATCTGCATTTGTGGTCTTGGCGTGTGGAGCCGCACACTGACTTGGAATGCCTTTCGATGCTGTCATTTCACCTGCGGCTTCGGTACCTGGCCCTCGCCTACAGAACAAGTAAGTAATTGGCACACCCGCGCAACTGTCGGAATGCTGGGATGGGTTGTGGCAGCTCGCGGGCCGTGGTGAGGGTGCGTGCCGTCAACTCACACCCTGGTGTACCCGCTCGCAGATACGAGATTATCTCGATTGTCGGAGAGGGCGCGTACGGCGTGGTCCTCAAATGCCGTAATAAGGAAACGGGAGAGATTGTAGCGGTGAAGAAGTTTAAAGAAAGCGACGGTAAGGGTGGGGGTTTGCGCCGGCCGGCAGTGGCGCTGTGGCTGTGGCAGTGTGGTTCGGCACGCTGACCCAACCCGCCCCTCACTAACGGATGCCGGACCTTCATGGGGATGCTTACAGAGGATGAGATTGTTCGCAAGACGACTCTCCGCGAGGTTAAGATGCTCCGGGCACTTCGGCAGGAGAACATTGTGAACCTAAAGGAGGCGTTTAGGCGGAAACAAAAGCTGGTATGGCTGGGATCGGCGGGTCCTCGTAACCACTGCGCATGGCACTTGATTTGTATCTGGCCAGGGCCAATATCTTGCAGCCTGCTGTGGGATATGCGCCAGATTCCCTAAGCCTGCCGCCGTACTGATGCATGCCTCCCTGCCACGGCGTCCACTCCTGCAGTACCTCGTGTTCGAGTACGTGGAACGGAATTTGCTGGAAATCTTGGAAGAGCATCCAGGGGGACTGGAGGGCGAGCAGGTGCGTCGTGCCTCGGCTGACGTGTCGGTGGCGCGCCGCGGGGCCGCGGGCTGGGGTGCCCACAACCCCGGCCATGAGCCCGCGGCGGCGGGTGGCTGAGGAAAGGCAGACACGGACACGGATAGTACACAGCAGCAAGCGGCATGGTTCTGGCGGAACGGCTTGGCATGGCGGGTGGGCGTGCGTGCCCACCGGACGCATGCACTGCGAGGGGACGCTTCCCGTGGCACTCGGATGGCACAGCAGCCTGGTGGCCAATGTGATCTGACTCGAAGCCGGTTGCTGCGCCTTTCTGCATGCGCAGGTGCGCAACTACATATACCAGCTCATCAAGGCGGTAGGGTGGTGCCACCAACACAACATCGTGCACCGCGACATCAAACCCGAGAACCTGCTCATCAGCCCGAGTGAGTGACCCTGCACGGTGACCGTGCCTGGCTGACTTATGCAGCATCACCACCACCTTCCCACTGCCCCTGTAGCTATCCACCTGTGCCCTTGGGGGTTCGCCACCGGCCCAGTTAGCCATTGGACACGTACGGCACCACCTGACCGTATCCTCCTCCCATTCCACCTCAACTCCCCACTCAGGCGCGGCTGGCGGCGTGGGCAAGCTTAAGCTGTGCGACTTTGGCTTTGCGCGGCAGCTGCCTCCCGCTGACGTATCCATCACTGACTACGTGTCCACTCGCTGGTACCGCGCGCCGGAGCTGCTGCTGGGCTCCACGCACTACGGCAAGGAGGTGGACCTGTGGGCTATTGGGTGAGTCCGGGAAGAGGGCAGGGAAACAGAGGGCACGGGGCAGAGGGGATGCGGGGGAGAAGGCAAGAGGGAGGAAACAGGAGGGAGGAGAGGCATGGAGAAAGGGAAGGGAGGAAAGGGTTGGGCATGGGCGAGGAGGGGATGGGCTTGGTTGCGTATGCCCGGCGTGGGTTGGGCCTGCTCAGCCCGTTCTTGGAGCTCACGTGGTCTGCCTGGTCACCCCGCAGGTGCATCATGGCTGAGCTGCTGGATGGCCAGCCGCTGTTCCCGGGCGAGAGTGACATCGACCAGCTCTACATACTGCAGCGGTTGCTGGGTGGGTGCCGACTGGAGCGTGCAAAGTGGGGTGGGGGTGTGGGGCCATGAGGGTATGGTCGGCGAGTTGTGAACGAGTGGTAGCGGAGGTGCCGTGACGCACGTTGGGGCAGAAGGACAATTGTGGTCCGCGGTTTCCAAACCTTGTCCCCCCCCCGCTCTATCGCTTCCGTACCCAGCCTTGTGTAACCCCATTCGCGCCCCTGTCCGGCCCCTCTCCGCAGGCCCGCTCACTCGCGAGCAGCACGACCTGTTCCTGCGCAACCCGCGCTTCAACGGCCTTAAGTTTCCCGACATGCGCAACCCGGAGACGCTGGACCGCAAGTACGCCGGCAAGATGCCGCACGACGCACTGGCGTTCATGAAGTGAGTGCGGGGCACATGGGGGGCAATTCAGCTGGTCGGGGCCCTGGCCATCATTGCACTGGAGTTGCACAGCACCGGTGTCCCAGGCTCTCTCTCCCGAGCCCCACGCATGTCCTCCATGACCGTGCCGCAACACCTCTCCCCCAAACACCACTAAAACCATCACCGTCACCATGTCCTCCTCCACCTTCGCCACCACCTCCAGGGCGCTGCTGGCTGTGGACCCCAGCGCGCGGCTGACCTGCAGCCAGGCGCTGTCGCACCCCTACCTGGCGGCGCTGGACGAGCGCTCGGGTGGCGGCGTGGGGCGGGCGGCCAGCAGCAGCGCGGCGCCGGCGGACTCGGGCGTGCGGCAGGGGCGCAAGGTCACGGCGGACCCCATGGACGAGGACATGCCCTCCCCGCCCGCCAGGTGGGGGCGCTGGGGAACCACGGCGGGATTGGGGGCGGAGGGTGGAGGCGGAGGGTGGAGCGCGGGCTTGGTATAGCGGGCTCGTGTGGGGCTGAGTGGTGGGAAGGGGTGCCGTGGCTGGCGACTGAAGTCGAGAGGGCTGGAGCCACCATCCGGGTACTGGGTAGCATCGCTGTGCACCTGGCATACGGCATGCACCCGGGCGCGCCATGCAAGATGTTGCTAACTCGAAGCTGACACAACGCACGAACTGCGCGCAGGCACGAGCCCATGGACCACGACATGTCGGACAACGAGTCCACCGCCTCCACAGTGGCGGTGGCGCGCCGCAAGGCAGCCGCCGCAGCCGCGGCCGCCTCCGGCGGCAAGGGCGGCAACGCCTCCTTCCGCGGCAGCGGCCGCCGCGACATCAACGAGATGCACGCAGCCGCCACCGCAGCCATGGGCGGCGGCGGCGGGCACGGCGCGGACCCGTACGGCAGCCGCCTGGACAGCGCGGGCTCCCGTGTGGGCACGCCGCAGCAGGGCAAGGGCGCGCTGCAGGCGGGCGGCTACGGCGGCCACCCGCAGCCGCGGCAGAGCCACCTGGGGCAGTCCATGGGCATGGGCGTGGCGGGGTACGGGCAGCAGAGCATGGAGCGCTTCAGCGCCTCCTCGCGTAACACGCCACAGGGCAGCACGGGCGGCAAGGCCGGCGCGGGCGGGCAGCAGCGGCTCAACGCCAGCCCGCCGCAGATGCACATGGACGGCGGTGGGTACGCGTCAGGTGGCCGCACGAGTATCGCGTCGTCTGGGCAGCCGGTGCTGTACCAGACCAATGCGGCTGCGGGCGCCAGTAAGCTCAGTCGCGCGCCCAGCCGCGGCGACCCCTGGCAACAGTCGGGCGGTGGGCAGCAAGGGCGCGGCGCGATGCCGCCGCTGCCGCCCGGCGGTGGGCCGCGCATGAGCGGGCACTGGGACGACGACGGCGGCAACCCGGAGCGGCCGTACTCGCGGGGGATGCTGGGCGGCGGCGGCGGGCCCATGCAGCCGGGCTTTGGGCAGAACCAGATGTGGCCGCAGCTCAACGTGCAGCAGCAGCAACAGCAGCAGCGGAGGGGCAATTACATGGTGAGCAAGGGCGAGGAGCTGTTCACCGGGGTGGTGCCCATCCTGGTCGAGCTGGACGGCGACGTAAACGGCCACAAGTTCAGCGTGCGCGGCGAGGGCGAGGGCGATGCCACCAACGGCAAGCTGACCCTGAAGTTCATCTGCACCACCGGCAAGCTGCCCGTGCCCTGGCCCACCCTCGTGACCACCCTGACCTACGGCGTGCAGTGCTTCAGCCGCTACCCCGACCACATGAAGCGCCACGACTTCTTCAAGTCCGCCATGCCCGAAGGCTACGTCCAGGAGCGCACCATCAGCTTCAAGGACGACGGCACCTACAAGACCCGCGCCGAGGTGAAGTTCGAGGGCGACACCCTGGTGAACCGCATCGAGCTGAAGGGCATCGACTTCAAGGAGGACGGCAACATCCTGGGGCACAAGCTGGAGTACAACTTCAACAGCCACAACGTCTATATCACCGCCGACAAGCAGAAGAACGGCATCAAGGCCAACTTCAAGATCCGCCACAACGTGGAGGACGGCAGCGTGCAGCTCGCCGACCACTACCAGCAGAACACCCCCATCGGCGACGGCCCCGTGCTGCTGCCCGACAACCACTACCTGAGCACCCAGTCCGTGCTGAGCAAAGACCCCAACGAGAAGCGCGATCACATGGTCCTGCTGGAGTTCGTGACCGCCGCCGGGATCACTCACGGCATGGACGAGCTGTACAAGTAGTAGCACAGTGACACTTACTGTGACGGCTTGCTTGCTAGAGGGGCAGCAGCTTCCCTACGCAAGCCCGGTTGTGTGCAGCGGGGCTGACAATGTCGGCGACAACAAAAGGACTGGACTGTACGGTACTTTCTGGTGGTGGATGCAGCCTCACTGCTAAAGACGCGGTCGTTTGCGCACTTCCGGCGTCCTGGTGGACTGCGGCTGACCAGCATTTGGCTCTGTGCAGCCGCTGACTGCGGCTTCGCTATTGTCACTGAAGCTCGATGACTACAGCTGGCGCCTGGGATTCTTAGCAGGATGCATGCGTCACGACATCCCTGGGGGGGACCGGGGAGGCGAGGGTTTGAGAGCGGGGCTTCCTATCTTGAACAGACATGTGAGGAGTGTGCATCATGAAGTAGGATGTCATTGTGATGACAAGAATGGCAGGACCGACAGGCGCCAACAGACACATGCGCCGTCGCCTGTTCGGGGGGGGGGGCGTCCTGCGGCACTGGTTTGCGGGCCAGCAGCTGCAGCTGACGGGCATGCATAAACAGTTGCACTTTTGAAACAGGAACGGTACGTATGGCGGCAAAGCAGGCTTTGTTTGGGACAACAAGACAAGACGCCCTGGGCAAGGGCTTTGTTAGCGGAATGCACCTGCGCGGCACCGCACTTGAGGACGTATGCACATGCGACTTGTTGCGTCCACGAGCTTGGCAGTGTTGACGCGCTGAAAGTAGCGGCGGTGGCATCTGAGAGCGGCCGTGACAAGGGGAGGAGGCATAGGCGTGTTGCTGTCGCACGCCGTCTAACAAGGGGACGCGCCGGCATTGCCATGGGCGGGACGCACGCGTGAGACCTTGCCGCTGACGCTGCGTGGTCATTGACCACATTTGGACGTGATACGGGATTTGGTGGGAGTTTGGTCTCTTCTTCTTACTCTTAAACGCTACTATGGGGGCGGGAAGGGGTGCGCATGCATAGCAATAAGCACGTTTGGCGTGAGAGGCAAAGATTGCGTCACACCCCTGCGCTTCTCAAAGCGAGTGGGCTTTCTTTCATTCTGGTTCTCGTCTTCCTACTTCTCTAGCCGGGGCTCGCAGTACAGCCCTGGCGCTACCCAATTGGCTAACATCGTTTGTAGACTGACGCTGTTGTGTTAGTTGGGGGCACGGTACGGGGGGCATGGTCGTGATGTGTCCACCACGCAATGTGCCACATGGCACCTTGCGTGGCACAGCGAAACGCTAACAAAGGTTCTGGAGTTCGTGATCCGTACACGTGCATGTAATGGACCAATGCTATCGCTTGAATGATTGTGATCACCGATTGAGCGTTGACTAGGAGTTCGAGCCTCCTTACTAGCTCTTTCTTGCGCTATGACACTTCCAGCAAAAGGTAGGGCGGGCTGCGAGACGGCTTCCCGGCGCTGCATGCAACACCGATGATGCTTCGACCCCCCGAAGCTCCTTCGGGGCTGCATGGGCGCTCCGATGCCGCTCCAGGGCGAGCGCTGTTTAAATAGCCAGGCCCCCGATTGCAAAGACATTATAGCGAGCTACCAAAGCCATATTCAAACACCTAGATCACTACCACTTCTACACAGGCCACTCGAGCTTGTGATCGCACTCCGCTAAGGGGGCGCCTCTTCCTCTTCGTTTCAGTCACAACCCGCAAACATGGTCGAGATTCGAAGCATGGACGATGCGTTGCGTGCACTGCGGGGTCGGTATCCCGGTTGTGAGTGGGTTGTTGTGGAGGATGGGGCCTCGGGGGCTGGTGTTTATCGGCTTCGGGGTGGTGGGCGGGAGTTGTTTGTCAAGGTGGCAGCTCTGGGGGCCGGGGTGGGCTTGTTGGGTGAGGCTGAgCGGCTGGTGTGGTTGGCGGAGGTGGGGATTCCCGTACCTCGTGTTGTGGAGGGTGGTGGGGACGAGAGGGTCGCCTGGTTGGTCACCGAAGCGGTTCCGGGGCGTCCGGCCAGTGCGCGGTGGCCGCGGGAGCAGCGGCTGGACGTGGCGGTGGCGCTCGCGGGGCTCGCTCGTTCGCTGCACGCGCTGGACTGGGAGCGGTGTCCGTTCGATCGCAGTCTCGCGGTGACGGTGCCGCAGGCGGCCCGTGCTGTCGCTGAAGGGAGCGTCGACTTGGAGGATCTGGACGAGGAGCGGAAGGGGTGGTCGGGGGAGCGGCTTCTCGCCGAGCTGGAGCGGACTCGGCCTGCGGACGAGGATCTGGCGGTTTGCCACGGTGACCTGTGCCCGGACAACGTGCTGCTCGACCCTCGTACCTGCGAGGTGACCGGGCTGATCGACGTGGGGCGGGTCGGCCGTGCGGACCGGCACTCCGATCTCGCGCTGGTGCTGCGCGAGCTGGCCCACGAGGAGGACCCGTGGTTCGGGCCGGAGTGTTCCGCGGCGTTCCTGCGGGAGTACGGGCGCGGGTGGGATGGGGCGGTATCGGAGGAAAAGCTGGCGTTTTACCGGCTGTTGGACGAGTTCTTCTGAGGGACCTGATGGTGTTGGTGGCTGGGTAGGGTTGCGTCGCGTGGGTGACAGCACAGTGTGGACGTTGGGATCCGGCAAGACTGGCCCCGCTTGGCAACGCAACAGTGAGCCCCTCCCTAGTGTGTTTGGGGATGTGACTATGTATTCGTGTGTTGGCCAACGGGTCAACCCGAACAGATTGATACCCGCCTTGGCATTTCCTGTCAGAATGTAACGTCAGTTGATGGTACCAGCTTTTGTTCCCTTTAGTGAGGGTTAATTtCGaGCTTGGCGTAATCATGGTCATAGCTGTTTCCTGTGTGAAATTGTTATCCGCTCACAATTCCACACAACATACGAGCCGGAAGCATAAAGTGTAAAGCCTGGGGTGCCTAATGAGTGAGCTAACTCACATTAATTGCGTTGCGCTCACTGCCCGCTTTCCAGTCGGGAAACCTGTCGTGCCAGCTGCATTAATGAATCGGCCAACGCGCGGGGAGAGGCGGTTTGCGTATTGGGCGCTCTTCCGCTTCCTCGCTCACTGACTCGCTGCGCTCGGTCGTTCGGCTGCGGCGAGCGGTATCAGCTCACTCAAAGGCGGTAATACGGTTATCCACAGAATCAGGGGATAACGCAGGAAAGAACATGGATCGGTACGATATCTTCGTCGATCGATATCGTACCGATCCATGTGAGCAAAAGGCCAGCAAAAGGCCAGGAACCGTAAAAAGGCCGCGTTGCTGGCGTTTTTCCATAGGCTCCGCCCCCCTGACGAGCATCACAAAAATCGACGCTCAAGTCAGAGGTGGCGAAACCCGACAGGACTATAAAGATACCAGGCGTTTCCCCCTGGAAGCTCCCTCGTGCGCTCTCCTGTTCCGACCCTGCCGCTTACCGGATACCTGTCCGCCTTTCTCCCTTCGGGAAGCGTGGCGCTTTCTCATAGCTCACGCTGTAGGTATCTCAGTTCGGTGTAGGTCGTTCGCTCCAAGCTGGGCTGTGTGCACGAACCCCCCGTTCAGCCCGACCGCTGCGCCTTATCCGGTAACTATCGTCTTGAGTCCAACCCGGTAAGACACGACTTATCGCCACTGGCAGCAGCCACTGGTAACAGGATTAGCAGAGCGAGGTATGTAGGCGGTGCTACAGAGTTCTTGAAGTGGTGGCCTAACTACGGCTACACTAGAAGGACAGTATTTGGTATCTGCGCTCTGCTGAAGCCAGTTACCTTCGGAAAAAGAGTTGGTAGCTCTTGATCCGGCAAACAAACCACCGCTGGTAGCGGTGGTTTTTTTGTTTGCAAGCAGCAGATTACGCGCAGAAAAAAAGGATCTCAAGAAGATCCTTTGATCTTTTCTACGGGGTCTGACGCTCAGTGGAACGAAAACTCACGTTAAGGGATTTTGGTCATGAGATTATCAAAAAGGATCTTCACCTAGATCCTTTTAAATTAAAAATGAAGTTTTAAATCAATCTAAAGTATATATGAGTAAACTTGGTCTGACAGTTACCAATGCTTAATCAGTGAGGCACCTATCTCAGCGATCTGTCTATTTCGTTCATCCATAGTTGCCTGACTCCCCGTCGTGTAGATAACTACGATACGGGAGGGCTTACCATCTGGCCCCAGTGCTGCAATGATACCGCGAGACCCACGCTCACCGGCTCCAGATTTATCAGCAATAAACCAGCCAGCCGGAAGGGCCGAGCGCAGAAGTGGTCCTGCAACTTTATCCGCCTCCATCCAGTCTATTAATTGTTGCCGGGAAGCTAGAGTAAGTAGTTCGCCAGTTAATAGTTTGCGCAACGTTGTTGCCATTGCTACAGGCATCGTGGTGTCACGCTCGTCGTTTGGTATGGCTTCATTCAGCTCCGGTTCCCAACGATCAAGGCGAGTTACATGATCCCCCATGTTGTGCAAAAAAGCGGTTAGCTCCTTCGGTCCTCCGATCGTTGTCAGAAGTAAGTTGGCCGCAGTGTTATCACTCATGGTTATGGCAGCACTGCATAATTCTCTTACTGTCATGCCATCCGTAAGATGCTTTTCTGTGACTGGTGAGTACTCAACCAAGTCATTCTGAGAATAGTGTATGCGGCGACCGAGTTGCTCTTGCCCGGCGTCAATACGGGATAATACCGCGCCACATAGCAGAACTTTAAAAGTGCTCATCATTGGAAAACGTTCTTCGGGGCGAAAACTCTCAAGGATCTTACCGCTGTTGAGATCCAGTTCGATGTAACCCACTCGTGCACCCAACTGATCTTCAGCATCTTTTACTTTCACCAGCGTTTCTGGGTGAGCAAAAACAGGAAGGCAAAATGCCGCAAAAAAGGGAATAAGGGCGACACGGAAATGTTGAATACTCATACTCTTCCTTTTTCAATATTATTGAAGCATTTATCAGGGTTATTGTCTCATGAGCGGATACATATTTGAATGTATTTAGAAAAATAAACAAATAGGGGTTCCGCGCACATTTCCCCGAAAAGTGCCAC

pLF5HA:

CTGACGCGCCCTGTAGCGGCGCATTAAGCGCGGCGGGTGTGGTGGTTACGCGCAGCGTGACCGCTACACTTGCCAGCGCCCTAGCGCCCGCTCCTTTCGCTTTCTTCCCTTCCTTTCTCGCCACGTTCGCCGGCTTTCCCCGTCAAGCTCTAAATCGGGGGCTCCCTTTAGGGTTCCGATTTAGTGCTTTACGGCACCTCGACCCCAAAAAACTTGATTAGGGTGATGGTTCACGTAGTGGGCCATCGCCCTGATAGACGGTTTTTCGCCCTTTGACGTTGGAGTCCACGTTCTTTAATAGTGGACTCTTGTTCCAAACTGGAACAACACTCAACCCTATCTCGGTCTATTCTTTTGATTTAAATTCATAACTTCGTATAATGTATGCTATACGAAGTTATGAATTAGGGATTTTGCCGATTTCGGCCTATTGGTTAAAAAATGAGCTGATTTAACAAAAATTTAACGCGAATTTTAACAAAATATTAACGCTTACAATTTCCATTCGCCATTCAGGCTGCGCAACTGTTGGGAAGGGCGATCGGTGCGGGCCTCTTCGCTATTACGCCAGCTGGCGAAAGGGGGATGTGCTGCAAGGCGATTAAGTTGGGTAACGCCAGGGTTTTCCCAGTCACGACGTTGTAAAACGACGGCCAGTGAATTGTAATACGACTCACTATAGGGCGAATTGGAGCTGACTGCTCGTGAATTCAAGGCGGTTGGCGGTAAGGTCACGTACTGGCCTCGCCGCCAAAAGTTCTGCGCAAGGCAGGAGGTTTTGCCACCTTGATTGAATCCATCGTTCACCCGTCCACGGTCACGGCCATGCATGCCGACATTTGCGGCCATGGCTGTTTGGGGTGCCAAGTGGTGCTTTGAGCGCTTGCTTTCTGGCTCATGACTTATGATGCGTTTCCTATCAGTTCCATGGCGGCACGAAGCTCTTGTTGAAATACGTGCCACCTGGCAGCGCGCGGCGGTCGTGTCTCCACGCGCCGCGCGCGGGCGGAAGGGTGGAAGTTTTATGCTCGACATGATTCGTTGTGCCCGAGTGAGTACAATACTATGCAGTGCAGCACACGTCGCTATATGGGTTTGTCAGGGCTTTGCTGCAGCGAGGCCTGCTGCAGCTGGGAACCGAATGAGCCGGGCGTCACCGATGTGGAAACGCAGTGTTCCTCGAGTCGCGAGCTTCGCGTGCCTTTGACTTGCAAAATTCACATGCCAACAACGTAGTATCGACTGATACAGCACTACATAAAGAGTCCTGGGCGAAAACGTTCACATCCATTTCGTCACCGCGGACACATATCGACTATCTTAAACCGATAATCTGAATTTCTACTTCTCAATTAGGCGCTTAGCCCATGACTGGGCTGACCTAGCGCGACCGCGGTCGCTTCGAAGGGCAGCCAAAGACTAGCACCCGAAGCCGCGTAGGGAGCCAGCAAGCCTTCGGGAATCGTAGACATCTTAATACAGCTTCTGTTATTGTGGGCAGCGGCGCGGGGAGGGCGCTGGAGCTCCGCTCGTGCTAGCTGGGCTCCCAGCAGCGCATGAGATGGTGCGTATTGGGGTCGATATTGCCAAGAACTCTTGCAACATCTGCATTTGTGGTCTTGGCGTGTGGAGCCGCACACTGACTTGGAATGCCTTTCGATGCTGTCATTTCACCTGCGGCTTCGGTACCTGGCCCTCGCCTACAGAACAAGTAAGTAATTGGCACACCCGCGCAACTGTCGGAATGCTGGGATGGGTTGTGGCAGCTCGCGGGCCGTGGTGAGGGTGCGTGCCGTCAACTCACACCCTGGTGTACCCGCTCGCAGATACGAGATTATCTCGATTGTCGGAGAGGGCGCGTACGGCGTGGTCCTCAAATGCCGTAATAAGGAAACGGGAGAGATTGTAGCGGTGAAGAAGTTTAAAGAAAGCGACGGTAAGGGTGGGGGTTTGCGCCGGCCGGCAGTGGCGCTGTGGCTGTGGCAGTGTGGTTCGGCACGCTGACCCAACCCGCCCCTCACTAACGGATGCCGGACCTTCATGGGGATGCTTACAGAGGATGAGATTGTTCGCAAGACGACTCTCCGCGAGGTTAAGATGCTCCGGGCACTTCGGCAGGAGAACATTGTGAACCTAAAGGAGGCGTTTAGGCGGAAACAAAAGCTGGTATGGCTGGGATCGGCGGGTCCTCGTAACCACTGCGCATGGCACTTGATTTGTATCTGGCCAGGGCCAATATCTTGCAGCCTGCTGTGGGATATGCGCCAGATTCCCTAAGCCTGCCGCCGTACTGATGCATGCCTCCCTGCCACGGCGTCCACTCCTGCAGTACCTCGTGTTCGAGTACGTGGAACGGAATTTGCTGGAAATCTTGGAAGAGCATCCAGGGGGACTGGAGGGCGAGCAGGTGCGTCGTGCCTCGGCTGACGTGTCGGTGGCGCGCCGCGGGGCCGCGGGCTGGGGTGCCCACAACCCCGGCCATGAGCCCGCGGCGGCGGGTGGCTGAGGAAAGGCAGACACGGACACGGATAGTACACAGCAGCAAGCGGCATGGTTCTGGCGGAACGGCTTGGCATGGCGGGTGGGCGTGCGTGCCCACCGGACGCATGCACTGCGAGGGGACGCTTCCCGTGGCACTCGGATGGCACAGCAGCCTGGTGGCCAATGTGATCTGACTCGAAGCCGGTTGCTGCGCCTTTCTGCATGCGCAGGTGCGCAACTACATATACCAGCTCATCAAGGCGGTAGGGTGGTGCCACCAACACAACATCGTGCACCGCGACATCAAACCCGAGAACCTGCTCATCAGCCCGAGTGAGTGACCCTGCACGGTGACCGTGCCTGGCTGACTTATGCAGCATCACCACCACCTTCCCACTGCCCCTGTAGCTATCCACCTGTGCCCTTGGGGGTTCGCCACCGGCCCAGTTAGCCATTGGACACGTACGGCACCACCTGACCGTATCCTCCTCCCATTCCACCTCAACTCCCCACTCAGGCGCGGCTGGCGGCGTGGGCAAGCTTAAGCTGTGCGACTTTGGCTTTGCGCGGCAGCTGCCTCCCGCTGACGTATCCATCACTGACTACGTGTCCACTCGCTGGTACCGCGCGCCGGAGCTGCTGCTGGGCTCCACGCACTACGGCAAGGAGGTGGACCTGTGGGCTATTGGGTGAGTCCGGGAAGAGGGCAGGGAAACAGAGGGCACGGGGCAGAGGGGATGCGGGGGAGAAGGCAAGAGGGAGGAAACAGGAGGGAGGAGAGGCATGGAGAAAGGGAAGGGAGGAAAGGGTTGGGCATGGGCGAGGAGGGGATGGGCTTGGTTGCGTATGCCCGGCGTGGGTTGGGCCTGCTCAGCCCGTTCTTGGAGCTCACGTGGTCTGCCTGGTCACCCCGCAGGTGCATCATGGCTGAGCTGCTGGATGGCCAGCCGCTGTTCCCGGGCGAGAGTGACATCGACCAGCTCTACATACTGCAGCGGTTGCTGGGTGGGTGCCGACTGGAGCGTGCAAAGTGGGGTGGGGGTGTGGGGCCATGAGGGTATGGTCGGCGAGTTGTGAACGAGTGGTAGCGGAGGTGCCGTGACGCACGTTGGGGCAGAAGGACAATTGTGGTCCGCGGTTTCCAAACCTTGTCCCCCCCCCGCTCTATCGCTTCCGTACCCAGCCTTGTGTAACCCCATTCGCGCCCCTGTCCGGCCCCTCTCCGCAGGCCCGCTCACTCGCGAGCAGCACGACCTGTTCCTGCGCAACCCGCGCTTCAACGGCCTTAAGTTTCCCGACATGCGCAACCCGGAGACGCTGGACCGCAAGTACGCCGGCAAGATGCCGCACGACGCACTGGCGTTCATGAAGTGAGTGCGGGGCACATGGGGGGCAATTCAGCTGGTCGGGGCCCTGGCCATCATTGCACTGGAGTTGCACAGCACCGGTGTCCCAGGCTCTCTCTCCCGAGCCCCACGCATGTCCTCCATGACCGTGCCGCAACACCTCTCCCCCAAACACCACTAAAACCATCACCGTCACCATGTCCTCCTCCACCTTCGCCACCACCTCCAGGGCGCTGCTGGCTGTGGACCCCAGCGCGCGGCTGACCTGCAGCCAGGCGCTGTCGCACCCCTACCTGGCGGCGCTGGACGAGCGCTCGGGTGGCGGCGTGGGGCGGGCGGCCAGCAGCAGCGCGGCGCCGGCGGACTCGGGCGTGCGGCAGGGGCGCAAGGTCACGGCGGACCCCATGGACGAGGACATGCCCTCCCCGCCCGCCAGGTGGGGGCGCTGGGGAACCACGGCGGGATTGGGGGCGGAGGGTGGAGGCGGAGGGTGGAGCGCGGGCTTGGTATAGCGGGCTCGTGTGGGGCTGAGTGGTGGGAAGGGGTGCCGTGGCTGGCGACTGAAGTCGAGAGGGCTGGAGCCACCATCCGGGTACTGGGTAGCATCGCTGTGCACCTGGCATACGGCATGCACCCGGGCGCGCCATGCAAGATGTTGCTAACTCGAAGCTGACACAACGCACGAACTGCGCGCAGGCACGAGCCCATGGACCACGACATGTCGGACAACGAGTCCACCGCCTCCACAGTGGCGGTGGCGCGCCGCAAGGCAGCCGCCGCAGCCGCGGCCGCCTCCGGCGGCAAGGGCGGCAACGCCTCCTTCCGCGGCAGCGGCCGCCGCGACATCAACGAGATGCACGCAGCCGCCACCGCAGCCATGGGCGGCGGCGGCGGGCACGGCGCGGACCCGTACGGCAGCCGCCTGGACAGCGCGGGCTCCCGTGTGGGCACGCCGCAGCAGGGCAAGGGCGCGCTGCAGGCGGGCGGCTACGGCGGCCACCCGCAGCCGCGGCAGAGCCACCTGGGGCAGTCCATGGGCATGGGCGTGGCGGGGTACGGGCAGCAGAGCATGGAGCGCTTCAGCGCCTCCTCGCGTAACACGCCACAGGGCAGCACGGGCGGCAAGGCCGGCGCGGGCGGGCAGCAGCGGCTCAACGCCAGCCCGCCGCAGATGCACATGGACGGCGGTGGGTACGCGTCAGGTGGCCGCACGAGTATCGCGTCGTCTGGGCAGCCGGTGCTGTACCAGACCAATGCGGCTGCGGGCGCCAGTAAGCTCAGTCGCGCGCCCAGCCGCGGCGACCCCTGGCAACAGTCGGGCGGTGGGCAGCAAGGGCGCGGCGCGATGCCGCCGCTGCCGCCCGGCGGTGGGCCGCGCATGAGCGGGCACTGGGACGACGACGGCGGCAACCCGGAGCGGCCGTACTCGCGGGGGATGCTGGGCGGCGGCGGCGGGCCCATGCAGCCGGGCTTTGGGCAGAACCAGATGTGGCCGCAGCTCAACGTGCAGCAGCAGCAACAGCAGCAGCGGAGGGGCAATTACGGCCTGTCGCGATACCCCTACGACGTGCCCGACTACGCCTACCCCTACGACGTGCCCGACTACGCCGATCGATCCGGACCGTACCCCTACGACGTGCCCGACTACGCCGCTTAGCACAGTGACACTTACTGTGACGGCTTGCTTGCTAGAGGGGCAGCAGCTTCCCTACGCAAGCCCGGTTGTGTGCAGCGGGGCTGACAATGTCGGCGACAACAAAAGGACTGGACTGTACGGTACTTTCTGGTGGTGGATGCAGCCTCACTGCTAAAGACGCGGTCGTTTGCGCACTTCCGGCGTCCTGGTGGACTGCGGCTGACCAGCATTTGGCTCTGTGCAGCCGCTGACTGCGGCTTCGCTATTGTCACTGAAGCTCGATGACTACAGCTGGCGCCTGGGATTCTTAGCAGGATGCATGCGTCACGACATCCCTGGGGGGGACCGGGGAGGCGAGGGTTTGAGAGCGGGGCTTCCTATCTTGAACAGACATGTGAGGAGTGTGCATCATGAAGTAGGATGTCATTGTGATGACAAGAATGGCAGGACCGACAGGCGCCAACAGACACATGCGCCGTCGCCTGTTCGGGGGGGGGGGCGTCCTGCGGCACTGGTTTGCGGGCCAGCAGCTGCAGCTGACGGGCATGCATAAACAGTTGCACTTTTGAAACAGGAACGGTACGTATGGCGGCAAAGCAGGCTTTGTTTGGGACAACAAGACAAGACGCCCTGGGCAAGGGCTTTGTTAGCGGAATGCACCTGCGCGGCACCGCACTTGAGGACGTATGCACATGCGACTTGTTGCGTCCACGAGCTTGGCAGTGTTGACGCGCTGAAAGTAGCGGCGGTGGCATCTGAGAGCGGCCGTGACAAGGGGAGGAGGCATAGGCGTGTTGCTGTCGCACGCCGTCTAACAAGGGGACGCGCCGGCATTGCCATGGGCGGGACGCACGCGTGAGACCTTGCCGCTGACGCTGCGTGGTCATTGACCACATTTGGACGTGATACGGGATTTGGTGGGAGTTTGGTCTCTTCTTCTTACTCTTAAACGCTACTATGGGGGCGGGAAGGGGTGCGCATGCATAGCAATAAGCACGTTTGGCGTGAGAGGCAAAGATTGCGTCACACCCCTGCGCTTCTCAAAGCGAGTGGGCTTTCTTTCATTCTGGTTCTCGTCTTCCTACTTCTCTAGCCGGGGCTCGCAGTACAGCCCTGGCGCTACCCAATTGGCTAACATCGTTTGTAGACTGACGCTGTTGTGTTAGTTGGGGGCACGGTACGGGGGGCATGGTCGTGATGTGTCCACCACGCAATGTGCCACATGGCACCTTGCGTGGCACAGCGAAACGCTAACAAAGGTTCTGGAGTTCGTGATCCGTACACGTGCATGTAATGGACCAATGCTATCGCTTGAATGATTGTGATCACCGATTGAGCGTTGACTAGGAGTTCGAGCCTCCTTACTAGCTCTTTCTTGCGCTATGACACTTCCAGCAAAAGGTAGGGCGGGCTGCGAGACGGCTTCCCGGCGCTGCATGCAACACCGATGATGCTTCGACCCCCCGAAGCTCCTTCGGGGCTGCATGGGCGCTCCGATGCCGCTCCAGGGCGAGCGCTGTTTAAATAGCCAGGCCCCCGATTGCAAAGACATTATAGCGAGCTACCAAAGCCATATTCAAACACCTAGATCACTACCACTTCTACACAGGCCACTCGAGCTTGTGATCGCACTCCGCTAAGGGGGCGCCTCTTCCTCTTCGTTTCAGTCACAACCCGCAAACATGGTCGAGATTCGAAGCATGGACGATGCGTTGCGTGCACTGCGGGGTCGGTATCCCGGTTGTGAGTGGGTTGTTGTGGAGGATGGGGCCTCGGGGGCTGGTGTTTATCGGCTTCGGGGTGGTGGGCGGGAGTTGTTTGTCAAGGTGGCAGCTCTGGGGGCCGGGGTGGGCTTGTTGGGTGAGGCTGAGCGGCTGGTGTGGTTGGCGGAGGTGGGGATTCCCGTACCTCGTGTTGTGGAGGGTGGTGGGGACGAGAGGGTCGCCTGGTTGGTCACCGAAGCGGTTCCGGGGCGTCCGGCCAGTGCGCGGTGGCCGCGGGAGCAGCGGCTGGACGTGGCGGTGGCGCTCGCGGGGCTCGCTCGTTCGCTGCACGCGCTGGACTGGGAGCGGTGTCCGTTCGATCGCAGTCTCGCGGTGACGGTGCCGCAGGCGGCCCGTGCTGTCGCTGAAGGGAGCGTCGACTTGGAGGATCTGGACGAGGAGCGGAAGGGGTGGTCGGGGGAGCGGCTTCTCGCCGAGCTGGAGCGGACTCGGCCTGCGGACGAGGATCTGGCGGTTTGCCACGGTGACCTGTGCCCGGACAACGTGCTGCTCGACCCTCGTACCTGCGAGGTGACCGGGCTGATCGACGTGGGGCGGGTCGGCCGTGCGGACCGGCACTCCGATCTCGCGCTGGTGCTGCGCGAGCTGGCCCACGAGGAGGACCCGTGGTTCGGGCCGGAGTGTTCCGCGGCGTTCCTGCGGGAGTACGGGCGCGGGTGGGATGGGGCGGTATCGGAGGAAAAGCTGGCGTTTTACCGGCTGTTGGACGAGTTCTTCTGAGGGACCTGATGGTGTTGGTGGCTGGGTAGGGTTGCGTCGCGTGGGTGACAGCACAGTGTGGACGTTGGGATCCGGCAAGACTGGCCCCGCTTGGCAACGCAACAGTGAGCCCCTCCCTAGTGTGTTTGGGGATGTGACTATGTATTCGTGTGTTGGCCAACGGGTCAACCCGAACAGATTGATACCCGCCTTGGCATTTCCTGTCAGAATGTAACGTCAGTTGATGGTACCAGCTTTTGTTCCCTTTAGTGAGGGTTAATTTCGAGCTTGGCGTAATCATGGTCATAGCTGTTTCCTGTGTGAAATTGTTATCCGCTCACAATTCCACACAACATACGAGCCGGAAGCATAAAGTGTAAAGCCTGGGGTGCCTAATGAGTGAGCTAACTCACATTAATTGCGTTGCGCTCACTGCCCGCTTTCCAGTCGGGAAACCTGTCGTGCCAGCTGCATTAATGAATCGGCCAACGCGCGGGGAGAGGCGGTTTGCGTATTGGGCGCTCTTCCGCTTCCTCGCTCACTGACTCGCTGCGCTCGGTCGTTCGGCTGCGGCGAGCGGTATCAGCTCACTCAAAGGCGGTAATACGGTTATCCACAGAATCAGGGGATAACGCAGGAAAGAACATGGATCGGTACGATATCTTCGTCGATCGATATCGTACCGATCCATGTGAGCAAAAGGCCAGCAAAAGGCCAGGAACCGTAAAAAGGCCGCGTTGCTGGCGTTTTTCCATAGGCTCCGCCCCCCTGACGAGCATCACAAAAATCGACGCTCAAGTCAGAGGTGGCGAAACCCGACAGGACTATAAAGATACCAGGCGTTTCCCCCTGGAAGCTCCCTCGTGCGCTCTCCTGTTCCGACCCTGCCGCTTACCGGATACCTGTCCGCCTTTCTCCCTTCGGGAAGCGTGGCGCTTTCTCATAGCTCACGCTGTAGGTATCTCAGTTCGGTGTAGGTCGTTCGCTCCAAGCTGGGCTGTGTGCACGAACCCCCCGTTCAGCCCGACCGCTGCGCCTTATCCGGTAACTATCGTCTTGAGTCCAACCCGGTAAGACACGACTTATCGCCACTGGCAGCAGCCACTGGTAACAGGATTAGCAGAGCGAGGTATGTAGGCGGTGCTACAGAGTTCTTGAAGTGGTGGCCTAACTACGGCTACACTAGAAGGACAGTATTTGGTATCTGCGCTCTGCTGAAGCCAGTTACCTTCGGAAAAAGAGTTGGTAGCTCTTGATCCGGCAAACAAACCACCGCTGGTAGCGGTGGTTTTTTTGTTTGCAAGCAGCAGATTACGCGCAGAAAAAAAGGATCTCAAGAAGATCCTTTGATCTTTTCTACGGGGTCTGACGCTCAGTGGAACGAAAACTCACGTTAAGGGATTTTGGTCATGAGATTATCAAAAAGGATCTTCACCTAGATCCTTTTAAATTAAAAATGAAGTTTTAAATCAATCTAAAGTATATATGAGTAAACTTGGTCTGACAGTTACCAATGCTTAATCAGTGAGGCACCTATCTCAGCGATCTGTCTATTTCGTTCATCCATAGTTGCCTGACTCCCCGTCGTGTAGATAACTACGATACGGGAGGGCTTACCATCTGGCCCCAGTGCTGCAATGATACCGCGAGACCCACGCTCACCGGCTCCAGATTTATCAGCAATAAACCAGCCAGCCGGAAGGGCCGAGCGCAGAAGTGGTCCTGCAACTTTATCCGCCTCCATCCAGTCTATTAATTGTTGCCGGGAAGCTAGAGTAAGTAGTTCGCCAGTTAATAGTTTGCGCAACGTTGTTGCCATTGCTACAGGCATCGTGGTGTCACGCTCGTCGTTTGGTATGGCTTCATTCAGCTCCGGTTCCCAACGATCAAGGCGAGTTACATGATCCCCCATGTTGTGCAAAAAAGCGGTTAGCTCCTTCGGTCCTCCGATCGTTGTCAGAAGTAAGTTGGCCGCAGTGTTATCACTCATGGTTATGGCAGCACTGCATAATTCTCTTACTGTCATGCCATCCGTAAGATGCTTTTCTGTGACTGGTGAGTACTCAACCAAGTCATTCTGAGAATAGTGTATGCGGCGACCGAGTTGCTCTTGCCCGGCGTCAATACGGGATAATACCGCGCCACATAGCAGAACTTTAAAAGTGCTCATCATTGGAAAACGTTCTTCGGGGCGAAAACTCTCAAGGATCTTACCGCTGTTGAGATCCAGTTCGATGTAACCCACTCGTGCACCCAACTGATCTTCAGCATCTTTTACTTTCACCAGCGTTTCTGGGTGAGCAAAAACAGGAAGGCAAAATGCCGCAAAAAAGGGAATAAGGGCGACACGGAAATGTTGAATACTCATACTCTTCCTTTTTCAATATTATTGAAGCATTTATCAGGGTTATTGTCTCATGAGCGGATACATATTTGAATGTATTTAGAAAAATAAACAAATAGGGGTTCCGCGCACATTTCCCCGAAAAGTGCCAC

pNAP1L1NmNeon3Flag:

GCGCCCAATACGCAAACCGCCTCTCCCCGCGCGTTGGCCGATTCATTAATGCAGCTGGCACGACAGGTTTCCCGACTGGAAAGCGGGCAGTGAGCGCAACGCAATTAATGTGAGTTAGCTCACTCATTAGGCACCCCAGGCTTTACACTTTATGCTTCCGGCTCGTATGTTGTGTGGAATTGTGAGCGGATAACAATTTCACACAGGAAACAGCTATGACCATGATTACGAATTCGATATCAAGCTTCTTTCTTGCGCTATGACACTTCCAGCAAAAGGTAGGGCGGGCTGCGAGACGGCTTCCCGGCGCTGCATGCAACACCGATGATGCTTCGACCCCCCGAAGCTCCTTCGGGGCTGCATGGGCGCTCCGATGCCGCTCCAGGGCGAGCGCTGTTTAAATAGCCAGGCCCCCGATTGCAAAGACATTATAGCGAGCTACCAAAGCCATATTCAAACACCTAGATCACTACCACTTCTACACAGGCCACTCGAGCTTGTGATCGCACTCCGCTAAGGGGGCGCCTCTTCCTCTTCGTTTCAGTCACAACCCGCAAACATGACACAAGAATCCCTGTTACTTCTCGACCGTATTGATTCGGATGATTCCTACGCGAGCCTGCGGAACGACCAGGAATTCTGGGAGGTGAGTCGACGAGCAAGCCCGGCGGATCAGGCAGCGTGCTTGCAGATTTGACTTGCAACGCCCGCATTGTGTCGACGAAGGCTTTTGGCTCCTCTGTCGCTGTCTCAAGCAGCATCTAACCCTGCGTCGCCGTTTCCATTTGCAGCCGCTGGCCCGCCGAGCCCTGGAGGAGCTCGGGCTGCCGGTGCCGCCGGTGCTGCGGGTGCCCGGCGAGAGCACCAACCCCGTACTGGTCGGCGAGCCCGGCCCGGTGATCAAGCTGTTCGGCGAGCACTGGTGCGGTCCGGAGAGCCTCGCGTCGGAGTCGGAGGCGTACGCGGTCCTGGCGGACGCCCCGGTGCCGGTGCCCCGCCTCCTCGGCCGCGGCGAGCTGCGGCCCGGCACCGGAGCCTGGCCGTGGCCCTACCTGGTGATGAGCCGGATGACCGGCACCACCTGGCGGTCCGCGATGGACGGCACGACCGACCGGAACGCGCTGCTCGCCCTGGCCCGCGAACTCGGCCGGGTGCTCGGCCGGCTGCACAGGGTGCCGCTGACCGGGAACACCGTGCTCACCCCCCATTCCGAGGTCTTCCCGGAACTGCTGCGGGAACGCCGCGCGGCGACCGTCGAGGACCACCGCGGGTGGGGCTACCTCTCGCCCCGGCTGCTGGACCGCCTGGAGGACTGGCTGCCGGACGTGGACACGCTGCTGGCCGGCCGCGAACCCCGGTTCGTCCACGGCGACCTGCACGGGACCAACATCTTCGTGGACCTGGCCGCGACCGAGGTCACCGGGATCGTCGACTTCACCGACGTCTATGCGGGAGACTCCCGCTACAGCCTGGTGCAACTGCATCTCAACGCCTTCCGGGGCGACCGCGAGATCCTGGCCGCGCTGCTCGACGGGGCGCAGTGGAAGCGGACCGAGGACTTCGCCCGCGAACTGCTCGCCTTCACCTTCCTGCACGACTTCGAGGTGTTCGAGGAGACCCCGCTGGATCTCTCCGGCTTCACCGATCCGGAGGAACTGGCGCAGTTCCTCTGGGGGCCGCCGGACACCGCCCCCGGCGCCTGATAAGGATCCCCGCTCCGTGTAAATGGAGGCGCTCGTTGATCTGAGCCTTGCCCCCTGACGAACGGCGGTGGATGGAAGATACTGCTCTCAAGTGCTGAAGCGGTAGCTTAGCTCCCCGTTTCGTGCTGATCAGTCTTTTTCAACACGTAAAAAGCGGAGGAGTTTTGCAATTTTGTTGGTTGTAACGATCCTCCGTTGATTTTGGCCTCTTTCTCCATGGGCGGGCTGGGCGTATTTGAAGCGACGAGGCCGCACACTGATGGAGGCTGGATGCTGGTGGAGGGACATGGTTGGGAAGTGCAGCGCGCTGACACGGCGGCGTGGTAGCTAGAGGGACATGGCGTCCTTGATTTGTCAAAGTATGGCTCACGCCTCACGGAACCCACGGAGACTCAAGCTCCAATGGGGATCAAATGCCTAACACGTTCAAAGGCTCTTCAAGGACATTACGTGGCTCTATTGGCGATGACCGCACTGTGTCGGTGGAGCGCGCATTTCACGGTGTAGATTATGCCGCGCCCCTTATCAGAAGCGGGTAAAGACACTCTCCTCGTATGTTGGATGCAAACGCAGGTGTGCGCAAGGTGAACAAACCGTCATACAGAAATAGGCGGCATGGAAAACTCGGCCGGAGCGAGAAGCGACCTCGTGAGTCGATGGGTTTACATGGGTACGTGCCGCATCAGACCGGATCCATGCAATATTACTTTGCGTCCAGACCTTATACGCATGCTTTCTTCTTCTAATAGCACAAAGAATGGTGTCCAAGGGCGAGGAGGACAACATGGCGAGCCTGCCCGCGACGCACGAGCTGCACATCTTCGGCAGCATCAACGGCGTGGACTTCGACATGGTGGGCCAGGGCACCGGCAACCCCAACGACGGCTACGAGGAGCTGAACCTGAAGTCCACGAAGGGCGACCTGCAGTTCAGCCCCTGGATTCTGGTGCCGCACATCGGCTACGGCTTCCACCAGTACCTGCCCTACCCGGACGGCATGAGCCCGTTCCAGGCCGCGATGGTGGACGGCTCGGGCTACCAGGTGCACCGCACCATGCAGTTCGAGGACGGCGCCTCCCTGACCGTGAACTACCGCTACACGTACGAGGGCAGCCACATCAAGGGCGAGGCCCAGGTGAAGGGCACCGGCTTCCCCGCGGACGGCCCGGTGATGACCAACTCGCTGACGGCCGCGGACTGGTGCCGCTCCAAGAAGACCTACCCCAACGACAAGACCATCATCAGCACGTTCAAGTGGTCGTACACCACGGGCAACGGCAAGCGCTACCGCTCGACCGCCCGCACCACGTACACGTTCGCGAAGCCCATGGCCGCGAACTACCTGAAGAACCAGCCGATGTACGTGTTCCGCAAGACCGAGCTGAAGCACAGCAAGACGGAGCTGAACTTCAAGGAGTGGCAGAAGGCGTTCACCGACGTGATGGGCATGGACGAGCTGTACAAGGGCGGTGGCGACTACAAGGACCATGACGGTGACTATAAGGATCACGACATCGACTACAAGGACGATGACGACAAGGGTGGCGGCATGTCGGGCGACAACGACACACAGCTGATTCAGGCAAGCTAGCTCGCAGGCGTCAAGCGTTCAATAGCCGCCTCGAGCGCCGCCCTGCTCAGTCGCTGGGCTCTGCATCACTTTTAATAAAGTTGTGCCTAAACTGCATTTGCACTAGCGGCTGAGTGCGTGGCGTGGTATTTCAAGCTGAAGGTTCAATCGTGAATGTTTCGCAGGCCAAGATGGCGACTCTGGGCCTGGACCAGACTCCAGAGGAGTTTGTTGCCGGCCTGGCTCCGCCCGTGAGGCGTCGCGTGGAGGCCCTGCAGGAGCTGCAGGCGAAGCATGACGAGCTGGAGGCACAGTTCCGCAAGGAGCGCGCCGAGCTGGAGGCCAAGTATGAGAAGCTTTACGGTGCGACTGCTGTGCGTTCGCGGTGTCGGAGCAGGGATTCCACATTTGTGCTCCAAAGGGTTTCGCGCCAGCCCAGGATCTTTCCCTTAAGGTGTTGCGCTGCTTTCGGCACTATTGTCATGCAGCTCCTCTGTACGTGGAGCGCTCTGAGATTGTGGTGGGCTCCAAGGAGGTGCCGCCCAAGGAGGGCGAGCCCACCGGCGACGGTGAGCGACCAAGCTGCTTTGGGCGACCTCTTACTGTCACGCAAGACGGTTTGATGGGACGTTGGCAGGATGGAAGGTGTAACCCCCTGGCGGAGCGAAGGGTGTGGCCGGGGTGCGGGTCTGGAGACACCGTCTGGCGCAACACCCTCGGCCAGCCCTAGCCAGCGCCCCCCGCGCCGCGCCTCATGCTTACCTCATGCCCATCTTACCTGTTGCATCCACACTAATGCACTTGTACAGACGCCATCAAGGGCATCCCTGAGTTCTGGCTGGCTGTGTTGCTCAAGTGCGAAGTGACCATGGACATGATCAAGGACAAGGACATGGACGTGCTCAAGTACCTGCGCGACATCCAGGTGGGCGGGCGGCCGGGGTACATATCGCAACTCCTGGCTGACCTCGTGTTGCCCTTGACAGAATGGGCCAAGTTAAGCGAAGTCGCGTCAACTTGGACCCCATCCATACCTGCATTACCCGCCCCCACAGGCCGAGGGCCTTGTGGTGGACGGCGTGTCTCACGGCTTCAAGCTGCGCTTCCTGTTCGACTCCAACCCCTACTTCACCAACGAGGTGCGGCGGAGGCTGGCGGGCGTGTGGCTAGGGCGCTATAGAGGGCCCGGCTGGCCTCGGGTGCAACTGCAGCAAGGCACGGGAGAGCGGGCGTCGTGGTGTTGGATGGTCTGCGCCTGCAAGTCTGTGCTACCCCGGCACGGTGCATGATGGCTTCCTGACAATGAGTCGTGAGCGCTACGACGCCATTACATGCTCCCTGGTGCTCCACCCTGTATGCCACTGCCCCGCCTGCTGCACCCAGGTGCTTGAGAAGACCTACCACATGCTGCCCGAGGACGACGGCGTGCTGGAGCGGGCGGAGGGCACCAAGATTGAGTGGAACGCGGGTGAGCGCGGCAAGCACTGCGATACGGTGGTTCTGCATAAGAGGGCAGCCGCAGTGTTACGCATACCTGCAGCCTACCCAGCATAGCCCGCCTCCCTCACAATACCTCCCAATGCGGAACAGGCAAGGACGTGACCGTTAAGATCATGAAGAAGAAGCCCAAGAAGGGCGGCAAGGGCGACTCCAAGCCCCAGGTGCGGGGCCTACGGGGGGGGGGGCGAGCCCAAAGCCCAATGCGCGCATTCCGATAGCAGTGGGCCTGGATTGATTATTGAATCACCGATACCTGTTATGATCCCGTCTAACCCTAAGCTACCTTGCGTGCCCGCCCCCCGCCCGCTCTGCAGGTCAAGACGGAGCGCGTGGACTCGTTCTTCAACTTCTTTGACCCGCCTCAGGTGCCGGACGGCGAGGAGGAGATCGACGAGGACACCATGGAGGAGCTGCAGGCCATCATCGAGGCTGACTACGAGGTGGGGGAGGGGCGGGGCGGGGAGCTTGGGCGGGGGCAGCTGCAGGAAATGGGTGGGAGATTGGCGAGGCTGGCTGCATGAGGCTTTTGGGCGGGTGGGGGCATGAGCGGTAGGCATACACACACACACTCTCTCTCTCGGTGGACTTGGAGCTCCTTGGCTCAGCAACCCGTAAAACTGTCTGCCAGGTAGCGCGGGTTCCAAACACGCTCTCTCCCCCTCCCCCTGGCTGTGTGCTGCACAGGTGGGCGCCACCATCCGGGAGAAGCTCATCCCGGAGGCGGTGTCCTGGTACACGGGCGAGGCCATGGACGAGGACGGGCTGTACATGCCCGGCGACGACGACGAGGACGACGAGGAGTGAGTGCCGCACTTGCGGATGTGCGGGCTCTGCTGTTTAGGGGGGGAGGGTAGCTGTTTGAAAGGTGGGAGTACGCAATTTGCACGGCGCGTCGCTGGAGACGTGGAAGGGCGGCAGCTATGTTGTTGCGTTGTGCTGTGCAAGGGTTTTACGGACGCCCCCAGGTCGCCTGTCAGGTGGCATGCCGGCTGCCTACCGCTAACTCGCCCGCACCCGTGTCATGCTGTGGGTTGCTCACTGTGTGCTTGCGTTTGCACTTGCCCCGCTATCGCAGCTTCGAGGGCGAGGAGGGCGAGGAGGACGAGGATGAGGACGAGGAGGGCGCGGGCGCGGGCGAGGGGCAAGCGGCGGGTGGGCAAGCGCAGCCGCCTGAGTGCAAGCAGCAGTAAGCGCTGCACAGACTGTGTGCAGTGGGGCGGGTCGGTTTCGTTGTGGTGTGGGGGAAGTTCATCATGAGCAAGGATTAGGGTGCTGGGTACTGAGAATGCACCACGACTGAATGCGTCAAATGCCAGTGTGTGCGGCTGCGGTGCAATTGAACAGCGCAGTCGGATGGAGGAAAGGTATAGACGTGGCGTAGGTACAGGTGCAAGAAAGCAGGCAGCGGTAGCATGCCTCGGCGTGTAAGGGAAGCTGAGTTCGTGTGCATCCTTGCGCGCTGGAGTTCTGACGCGGGCAATGGTCAAGAGCTGTGATATGGACGCCGGACGACAAGCTTTGCTCTCCTCCCTGGATGCCGGACTGGCCACCTGAAGACCAGCAGGGGGTACCGGGCCCGTCATCCCATGGAAGCTTGGCACTGGCCGTCGTTTTACAACGTCGTGACTGGGAAAACCCTGGCGTTACCCAACTTAATCGCCTTGCAGCACATCCCCCTTTCGCCAGCTGGCGTAATAGCGAAGAGGCCCGCACCGATCGCCCTTCCCAACAGTTGCGCAGCCTGAATGGCGAATGGCGCCTGATGCGGTATTTTCTCCTTACGCATCTGTGCGGTATTTCACACCGCATATGGTGCACTCTCAGTACAATCTGCTCTGATGCCGCATAGTTAAGCCAGCCCCGACACCCGCCAACACCCGCTGACGCGCCCTGACGGGCTTGTCTGCTCCCGGCATCCGCTTACAGACAAGCTGTGACCGTCTCCGGGAGCTGCATGTGTCAGAGGTTTTCACCGTCATCACCGAAACGCGCGAGACGAAAGGGCCTCGTGATACGCCTATTTTTATAGGTTAATGTCATGATAATAATGGTTTCTTAGACGTCAGGTGGCACTTTTCGGGGAAATGTGCGCGGAACCCCTATTTGTTTATTTTTCTAAATACATTCAAATATGTATCCGCTCATGAGACAATAACCCTGATAAATGCTTCAATAATATTGAAAAAGGAAGAGTATGAGTATTCAACATTTCCGTGTCGCCCTTATTCCCTTTTTTGCGGCATTTTGCCTTCCTGTTTTTGCTCACCCAGAAACGCTGGTGAAAGTAAAAGATGCTGAAGATCAGTTGGGTGCACGAGTGGGTTACATCGAACTGGATCTCAACAGCGGTAAGATCCTTGAGAGTTTTCGCCCCGAAGAACGTTTTCCAATGATGAGCACTTTTAAAGTTCTGCTATGTGGCGCGGTATTATCCCGTATTGACGCCGGGCAAGAGCAACTCGGTCGCCGCATACACTATTCTCAGAATGACTTGGTTGAGTACTCACCAGTCACAGAAAAGCATCTTACGGATGGCATGACAGTAAGAGAATTATGCAGTGCTGCCATAACCATGAGTGATAACACTGCGGCCAACTTACTTCTGACAACGATCGGAGGACCGAAGGAGCTAACCGCTTTTTTGCACAACATGGGGGATCATGTAACTCGCCTTGATCGTTGGGAACCGGAGCTGAATGAAGCCATACCAAACGACGAGCGTGACACCACGATGCCTGTAGCAATGGCAACAACGTTGCGCAAACTATTAACTGGCGAACTACTTACTCTAGCTTCCCGGCAACAATTAATAGACTGGATGGAGGCGGATAAAGTTGCAGGACCACTTCTGCGCTCGGCCCTTCCGGCTGGCTGGTTTATTGCTGATAAATCTGGAGCCCGTGAGCGTGGGTCTCGCGGTATCATTGCAGCACTGGGGCCAGATGGTAAGCCCTCCCGTATCGTAGTTATCTACACGACGGGGAGTCAGGCAACTATGGATGAACGAAATAGACAGATCGCTGAGATAGGTGCCTCACTGATTAAGCATTGGTAACTGTCAGACCAAGTTTACTCATATATACTTTAGATTGATTTAAAACTTCATTTTTAATTTAAAAGGATCTAGGTGAAGATCCTTTTTGATAATCTCATGACCAAAATCCCTTAACGTGAGTTTTCGTTCCACTGAGCGTCAGACCCCGTAGAAAAGATCAAAGGATCTTCTTGAGATCCTTTTTTTCTGCGCGTAATCTGCTGCTTGCAAACAAAAAAACCACCGCTACCAGCGGTGGTTTGTTTGCCGGATCAAGAGCTACCAACTCTTTTTCCGAAGGTAACTGGCTTCAGCAGAGCGCAGATACCAAATACTGTCCTTCTAGTGTAGCCGTAGTTAGGCCACCACTTCAAGAACTCTGTAGCACCGCCTACATACCTCGCTCTGCTAATCCTGTTACCAGTGGCTGCTGCCAGTGGCGATAAGTCGTGTCTTACCGGGTTGGACTCAAGACGATAGTTACCGGATAAGGCGCAGCGGTCGGGCTGAACGGGGGGTTCGTGCACACAGCCCAGCTTGGAGCGAACGACCTACACCGAACTGAGATACCTACAGCGTGAGCTATGAGAAAGCGCCACGCTTCCCGAAGGGAGAAAGGCGGACAGGTATCCGGTAAGCGGCAGGGTCGGAACAGGAGAGCGCACGAGGGAGCTTCCAGGGGGAAACGCCTGGTATCTTTATAGTCCTGTCGGGTTTCGCCACCTCTGACTTGAGCGTCGATTTTTGTGATGCTCGTCAGGGGGGCGGAGCCTATGGAAAAACGCCAGCAACGCGGCCTTTTTACGGTTCCTGGCCTTTTGCTGGCCTTTTGCTCACATGTTCTTTCCTGCGTTATCCCCTGATTCTGTGGATAACCGTATTACCGCCTTTGAGTGAGCTGATACCGCTCGCCGCAGCCGAACGACCGAGCGCAGCGAGTCAGTGAGCGAGGAAGCGGAAGA
