# Supplementary material for: Direct in situ protein tagging in Chlamydomonas reinhardtii utilizing TIM, a method for CRISPR/Cas9-based targeted insertional mutagenesis
Source: PLoS One. 2022 Dec 9;17(12):e0278972. doi: 10.1371/journal.pone.0278972 (PMC9733891; doi:10.1371/journal.pone.0278972)
Supplement: S1 Table — (DOCX) [file pone.0278972.s006.docx]

S1 Table: List of gRNAs and primers

| Name | Sequence 5’-3’ | Description |
| --- | --- | --- |
| LF5 gRNA | GGTGCTGTACCAGACCAATG | RNP recognition site |
| LF5donor-1 | AGCCGGTGCTGTACCAGACCAATGCCGCTGCGGGCGCCAGTAAG | Amplification of donor DNA for LF5 tagging |
| LF5donor-2 | GCTGGTACCATCAACTGACGTTAC |  |
| LF5-37 | CACGAGTATCGCGTCGTCTG | PCR screening for LF5 tagging |
| LF5-28 | CAGTCCAGTCCTTTTGTTGTCG | Paired with LF5-37 for initial PCR screen |
| LF5-30 | GGCTCGAACTCCTAGTCAACG | Paired with LF5-37 to amplify 3’ UTR |
| LF5-1 | CTATAGGGCGAATTGGAGCTGACTGCTCGTGAATTCAA | pLF5CsfGFP cloning |
| LF5-6 | CTCGCCCTTGCTCACCATGTAATTGCCCCTCCGCTG | pLF5CsfGFP cloning |
| LF5-4 | TCATAGCGCAAGAAAGAGCTAGTAAGGAGGCTCGAACTCC | pLF5CsfGFP cloning |
| LF5-5 | GCATGGACGAGCTGTACAAGTAGTAGCACAGTGACACTTACTG | pLF5CsfGFP cloning |
| sfGFP-1 | ATGGTGAGCAAGGGCGA | pLF5CsfGFP cloning |
| sfGFP-2 | CTTGTACAGCTCGTCCATGC | pLF5CsfGFP cloning |
| LF5-20 | GGGTATCGCGACAGGCCGTAATTGCCCCTCCGCTG | pLF5HA cloning |
| LF5-31 | TGCCCGACTACGCCGCTTAGCACAGTGACACTTACTG | pLF5HA cloning |
| AphVIII-1 | GTGGGATGGGGCGGTATC | Sequencing |
| AphVIII-2 | TCACAACCGGGATACCGAC | Sequencing |
| AphVIII-3 | GTCGGTATCCCGGTTGTGA | Paired with LF5-28 to amplify the region surrounding the site where the 3’ end of the donor DNA integrated in the HA-tagged strains |
| AphVIII-4 | GGCCGGACGCCCCGGAAC | Sequencing |
| LF5-39 | CTTTGGGCAGAACCAGATGT | Sequencing |
| NAP1L1 gRNA1 | GTTATGCTGACTTGCCACGC | RNP1 recognition site |
| NAP1L1 gRNA2 | GGCGTGGTATTTCAAGCTGA | RNP2 recognition site |
| NAP1L1donor-1-2 | CCTTGTTATGCTGACTTGCCA GACCATGATTACGAATTCGA | Amplification of donor DNA for NAP1L1 tagging |
| NAP1L1donor-2-2 | GCGAAACATTCACGATTGAACGTTCAGCTTGAAATACCACGC |  |
| NAP1L1-3 | TGGGCGTATTTGAAGCGGGTACGAGGCCGCACACTGATGGAG | pNAP1L1NmNeon3Flag cloning |
| NAP1L1-4 | CTCCTCGCCCTTGGACACCATTCTTTGTGCTATTAGAAGAAG | pNAP1L1NmNeon3Flag cloning |
| NAP1L1-5 | GATGACGACAAGGGTGGCGGCATGTCGGGCGACAACGACA | pNAP1L1NmNeon3Flag cloning |
| NAP1L1-6 | ATGGGATGACGGGCCCGGTACCCCCTGCTGGTCTTCAGGTG | pNAP1L1NmNeon3Flag cloning |
| PLM160-1 | ATGGTGTCCAAGGGCGAGGA | pNAP1L1NmNeon3Flag cloning |
| PLM160-2 | GCCGCCACCCTTGTCGTCATCGTCCTTGT | pNAP1L1NmNeon3Flag cloning |
| PLM160-5 | ATGTACGTGTTCCGCAAGAC | PCR screening for NAP1L1 tagging |
| NAP1L1-12 | CGGCAACAAACTCCTCTGGA |  |
| NAP1L1-19 | GTTGGGCCCTGATAGCATGT | PCR screening for NAP1L1 tagging |
| NAP1L1-18 | CCAGAATTCCTGGTCGTTCC |  |
| Fus-3 | TCCAACGCATAGCCATCAAC | Mating type plus specific gene as control |
| Fus-4 | TGTTTGCTAGGGGTGCAATG |  |
| NAP1L1-25 | TGGGCCCATATCAACAAACA | Amplification of DNA substrate for *in vitro* Cas9/gRNA assay |
| NAP1L1-22 | ACACAGCCAGCCAGAACTCA |  |
