# Supplementary material for: Direct in situ protein tagging in Chlamydomonas reinhardtii utilizing TIM, a method for CRISPR/Cas9-based targeted insertional mutagenesis
Source: PLoS One. 2022 Dec 9;17(12):e0278972. doi: 10.1371/journal.pone.0278972 (PMC9733891; doi:10.1371/journal.pone.0278972)
Supplement: S1 Fig — (A) Schematic diagram illustrating how vector pLF5CsfGFP was generated. Fragments 1 and 2 were amplified from the plasmid pBS3830, which contains the LF5 gene. Fragment 1 contains the 5’ end of the LF5 gene up to the stop codon while fragment 2 contains the 3’ end of the LF5 gene starting with the stop codon. Fragment 3, which contains the coding region for sfGFP, was amplified from the plasmid pIFT140-sfGFP-aphVIII. Fragment 4, containing the paromomycin cassette, was created by linearizing plasmid pKS-aphVIII-lox with SacI. The four fragments were ligated together using NEBuilder® HiFi DNA Assembly Master Mix to produce the vector pLF5CsfGFP. The LF5 gene is indicated by a wide grey line, sfGFP is indicated by a wide orange line, and the paromomycin cassette is indicated by a wide green line. The primers are shown above the corresponding amplification regions as short arrows with thickness and color corresponding to the plasmid sequence that they match. The red star represents the stop codon of the LF5 gene. (B) Schematic representation of how vector pLF5HA was made. All of plasmid pLF5CsfGFP except for the sfGFP sequence was amplified by PCR to generate Fragment pLF5CsfGFPΔsfGFP. The 3HA sequence was cut from plasmid p3HA. These two fragments were ligated using NEBuilder® HiFi DNA Assembly Master Mix to produce the vector pLF5HA. The LF5 gene is indicated by a wide grey line, sfGFP is indicated by a wide orange line, the paromomycin cassette is indicated by a wide green line, and sequence encoding the 3HA tag is indicated by a wide blue line. The primers are shown below the corresponding amplification regions as short arrows with thickness and color corresponding to the plasmid sequence that they match. The red star represents the stop codon of the LF5 gene. (PDF) [file pone.0278972.s007.pdf]

S1A_Fig

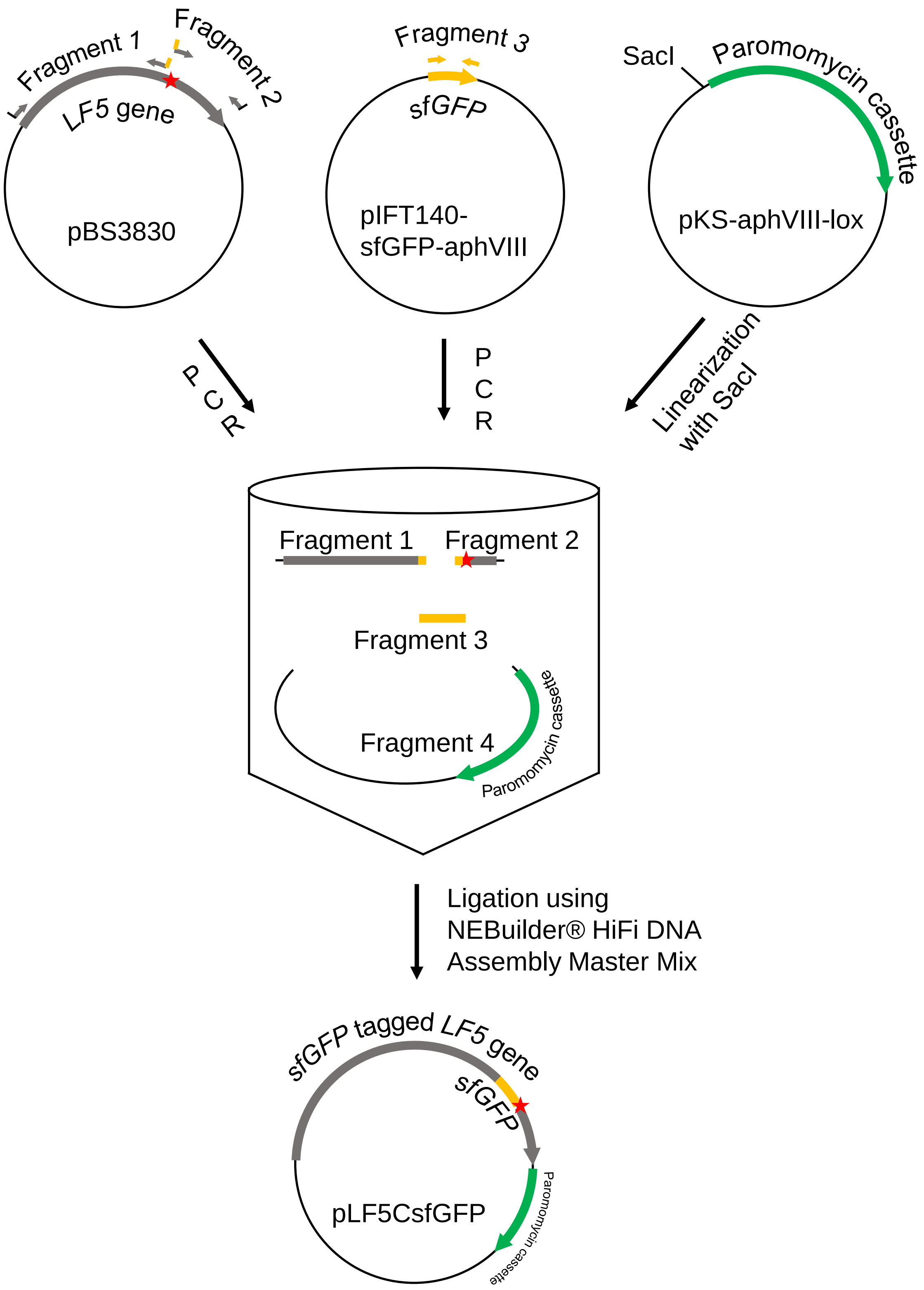

Fragment 1
Fragment 2
LF5 gene
pBS3830
Fragment 3
sfGFP
pIFT140-
sfGFP-aphVIII
SacI
Paromomycin cassette
pKS-aphVIII-lox
PCR
PCR
Linearization
with SacI
Fragment 1
Fragment 2
Fragment 3
Fragment 4
Paromomycin cassette
Ligation using
NEBuilder® HiFi DNA
Assembly Master Mix
sfGFP tagged LF5 gene
sfGFP
pLF5CsfGFP
Paromomycin cassette

S1B_Fig

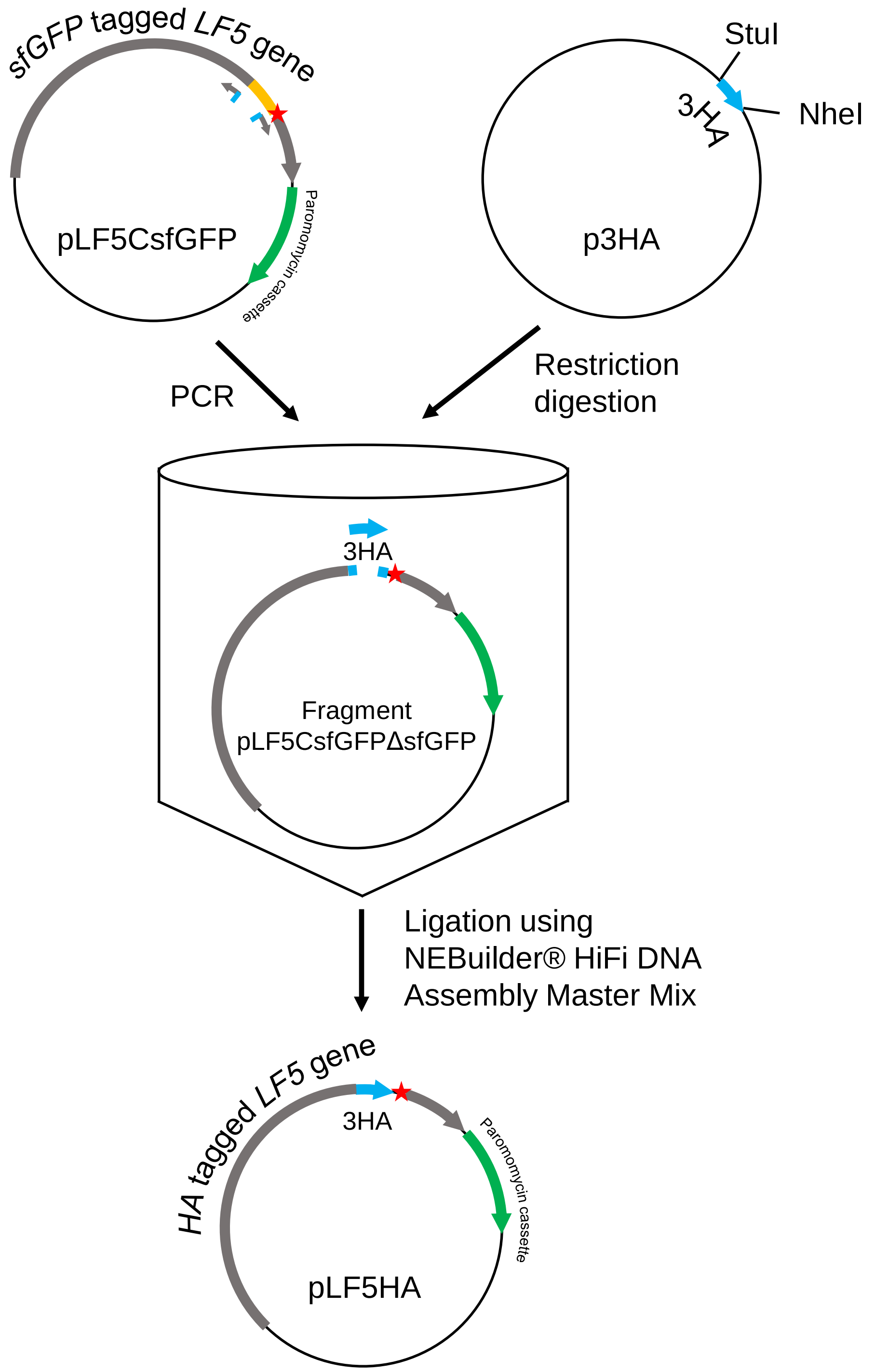
sfGFP tagged LF5 gene
pLF5CsfGFP
Paromomycin cassette
StuI
NheI
3HA
p3HA
PCR
Restriction
digestion
3HA
Fragment
pLF5CsfGFPΔsfGFP
Ligation using
NEBuilder® HiFi DNA
Assembly Master Mix
HA tagged LF5 gene
3HA
Paromomycin cassette
pLF5HA

S1 Fig. Schematic representation of vector construction. (A) Schematic diagram illustrating how vector pLF5CsfGFP was generated. Fragments 1 and 2 were amplified from the plasmid pBS3830, which contains the *LF5* gene. Fragment 1 contains the 5' end of the *LF5* gene up to the stop codon while fragment 2 contains the 3' end of the *LF5* gene starting with the stop codon. Fragment 3, which contains the coding region for sfGFP, was amplified from the plasmid pIFT140-sfGFP-aphVIII. Fragment 4, containing the paromomycin cassette, was created by linearizing plasmid pKS-aphVIII-lox with SacI. The four fragments were ligated together using NEBuilder® HiFi DNA Assembly Master Mix to produce the vector pLF5CsfGFP. The *LF5* gene is indicated by a wide grey line, sfGFP is indicated by a wide orange line, and the paromomycin cassette is indicated by a wide green line. The primers are shown above the corresponding amplification regions as short arrows with thickness and color corresponding to the plasmid sequence that they match. The red star represents the stop codon of the *LF5* gene. (B) Schematic representation of how vector pLF5HA was made. All of plasmid pLF5CsfGFP except for the sfGFP sequence was amplified by PCR to generate Fragment pLF5CsfGFPΔsfGFP. The 3HA sequence was cut from plasmid p3HA. These two fragments were ligated using NEBuilder® HiFi DNA Assembly Master Mix to produce the vector pLF5HA. The *LF5* gene is indicated by a wide grey line, sfGFP is indicated by a wide orange line, the paromomycin cassette is indicated by a wide green line, and sequence encoding the 3HA tag is indicated by a wide blue line. The primers are shown below the corresponding amplification regions as short arrows with thickness and color corresponding to the plasmid sequence that they match. The red star represents the stop codon of the *LF5* gene.
