# Supplementary material for: Direct in situ protein tagging in Chlamydomonas reinhardtii utilizing TIM, a method for CRISPR/Cas9-based targeted insertional mutagenesis
Source: PLoS One. 2022 Dec 9;17(12):e0278972. doi: 10.1371/journal.pone.0278972 (PMC9733891; doi:10.1371/journal.pone.0278972)
Supplement: S2 Fig — To confirm that the lack of PCR products was caused by disruption of the LF5 gene, genomic DNA from strains that did not yield any product in the initial PCR screening for LF5HA strains (Fig 5) were amplified using primer pairs specific to the LF5 locus and to mating-type-plus-specific gene FUS1 as positive control. While primers specific for FUS1 yielded products for all the transformants, there was no amplification using primers specific for LF5, indicating that the LF5 locus was disrupted in these transformants. W: wild-type strain g1 as control; HA: plasmid containing LF5HA as template; M: NEB 100 bp DNA ladder (catalog number N0551). (DOCX) [file pone.0278972.s008.docx]

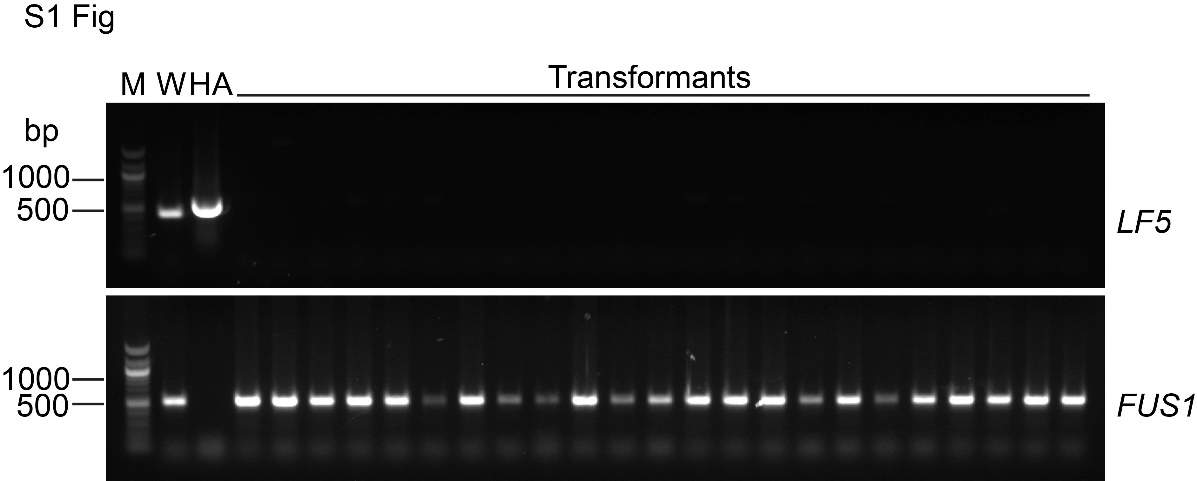


**S2 Fig. PCR analysis of transformants that yielded no band during initial PCR screen for LF5-tagged strains confirmed that the *LF5* locus was disrupted.** To confirm that the lack of PCR products was caused by disruption of the *LF5* gene, genomic DNA from strains that did not yield any product in the initial PCR screening for LF5HA strains (Fig 5) were amplified using primer pairs specific to the *LF5* locus and to mating-type-plus-specific gene *FUS1* as positive control. While primers specific for *FUS1* yielded products for all the transformants, there was no amplification using primers specific for *LF5*, indicating that the *LF5* locus was disrupted in these transformants. W: wild-type strain g1 as control; HA: plasmid containing *LF5HA* as template; M: NEB 100 bp DNA ladder (catalog number N0551).
