# Supplementary material for: Direct in situ protein tagging in Chlamydomonas reinhardtii utilizing TIM, a method for CRISPR/Cas9-based targeted insertional mutagenesis
Source: PLoS One. 2022 Dec 9;17(12):e0278972. doi: 10.1371/journal.pone.0278972 (PMC9733891; doi:10.1371/journal.pone.0278972)
Supplement: S3 Fig — NAP1L1 DNA was amplified and digested in vitro as described in the Materials and Methods, and the digestion products were separated on a 1.2% agarose gel. The NAP1L1 DNA substrate is 2.8 kb in size; RNP1 was predicted to yield fragments of 1322 bp and 1478 bp, while RNP2 was predicted to yield fragments of 2109 bp and 691 bp. In the experiment, each individual RNP completely digested the substrate, yielding products of the expected sizes. RNP1 and RNP2 together completely digested the substrate into three fragments of 1322 bp, 787 bp, and 691 bp, indicating that the two RNPs can cut the same fragment in vitro. C: NAP1L1 DNA only. M1: 1 kb DNA ladder (NEB catalog #N0552). M2: 100 bp DNA ladder (NEB catalog #N0551); from top to bottom, markers are 1517, 1200, 1000, 900, 800, 700, 600, 500/517, 400, 300, 200, and 100 bp. (DOCX) [file pone.0278972.s009.docx]

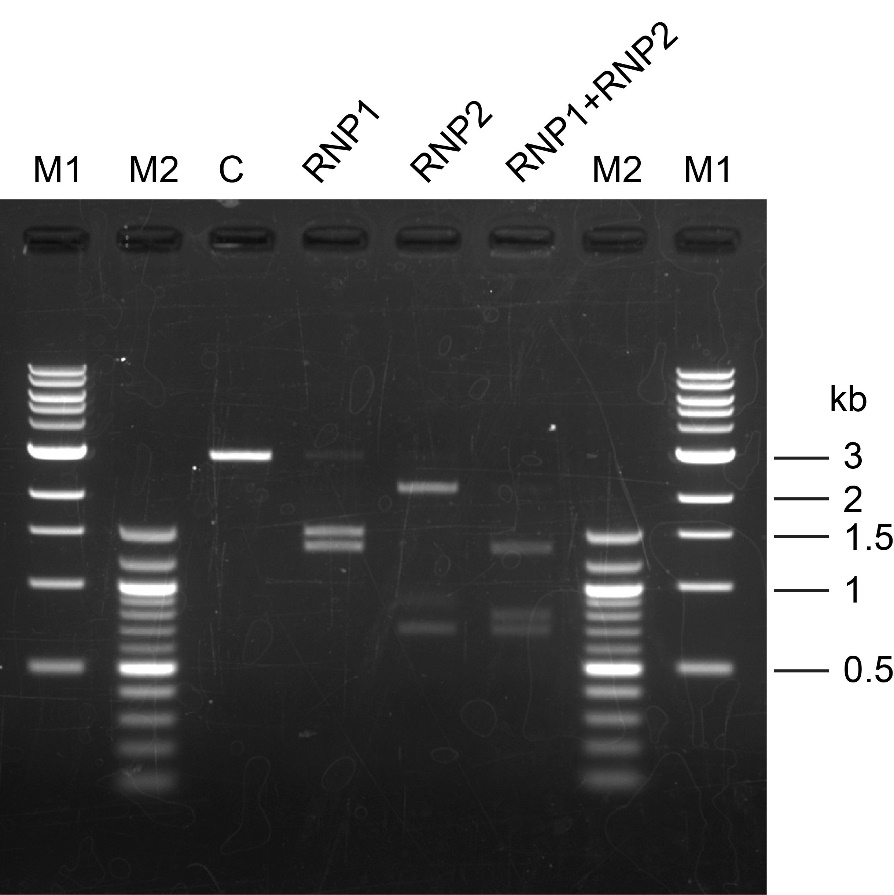


**S3 Fig. Both NAP1L1 RNP1 and RNP2 cut *NAP1L1* DNA targets *in vitro*.** *NAP1L1* DNA was amplified and digested *in vitro* as described in the Materials and Methods, and the digestion products were separated on a 1.2% agarose gel. The *NAP1L1* DNA substrate is 2.8 kb in size; RNP1 was predicted to yield fragments of 1322 bp and 1478 bp, while RNP2 was predicted to yield fragments of 2109 bp and 691 bp. In the experiment, each individual RNP completely digested the substrate, yielding products of the expected sizes. RNP1 and RNP2 together completely digested the substrate into three fragments of 1322 bp, 787 bp, and 691 bp, indicating that the two RNPs can cut the same fragment *in vitro*. C: *NAP1L1* DNA only. M1: 1 kb DNA ladder (NEB catalog #N0552). M2: 100 bp DNA ladder (NEB catalog #N0551); from top to bottom, markers are 1517, 1200, 1000, 900, 800, 700, 600, 500/517, 400, 300, 200, and 100 bp.
