# Supplementary material for: Direct in situ protein tagging in Chlamydomonas reinhardtii utilizing TIM, a method for CRISPR/Cas9-based targeted insertional mutagenesis
Source: PLoS One. 2022 Dec 9;17(12):e0278972. doi: 10.1371/journal.pone.0278972 (PMC9733891; doi:10.1371/journal.pone.0278972)

Raw images for Fig 5. The images of DNA agarose gels were taken using an Alpha Innotech FluorChem Q system. Individual panels were cropped from the raw images as defined by the white squares. The cropped panels were then inverted and adjusted for contrast using Photoshop; contrast adjustment was applied evenly across the entire image. The adjusted panels were assembled into Fig 5 using Adobe Illustrator. M: DNA marker. Panels here are arranged in the same sequence as in Fig 5.

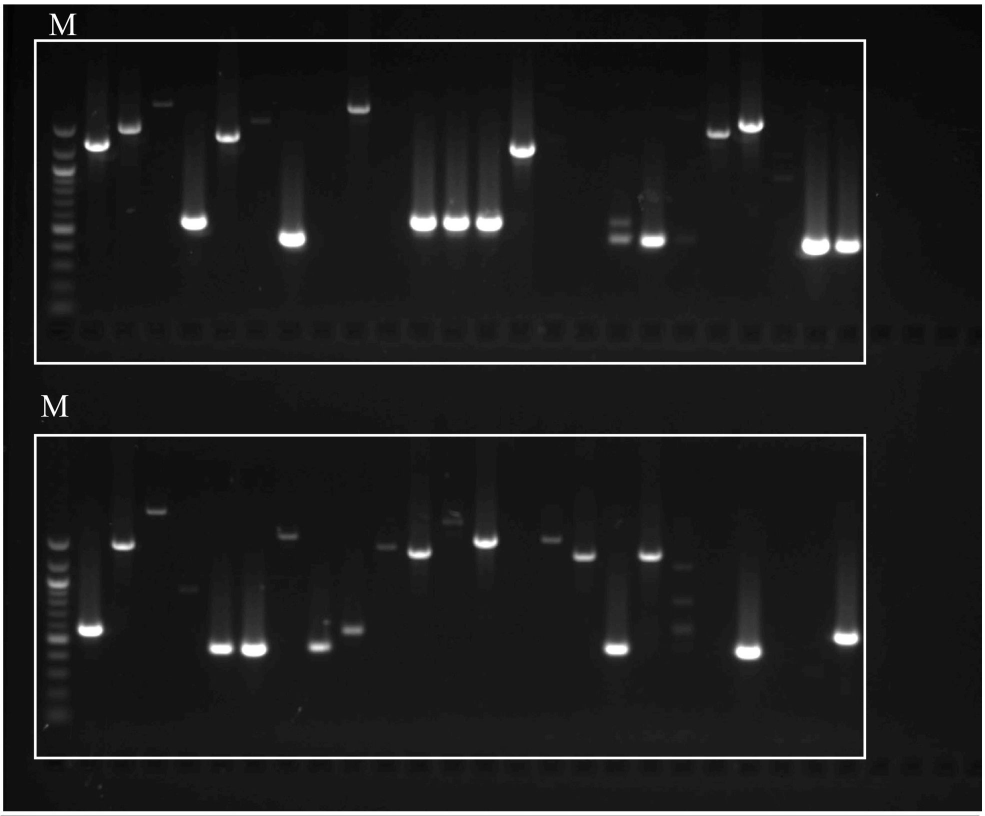

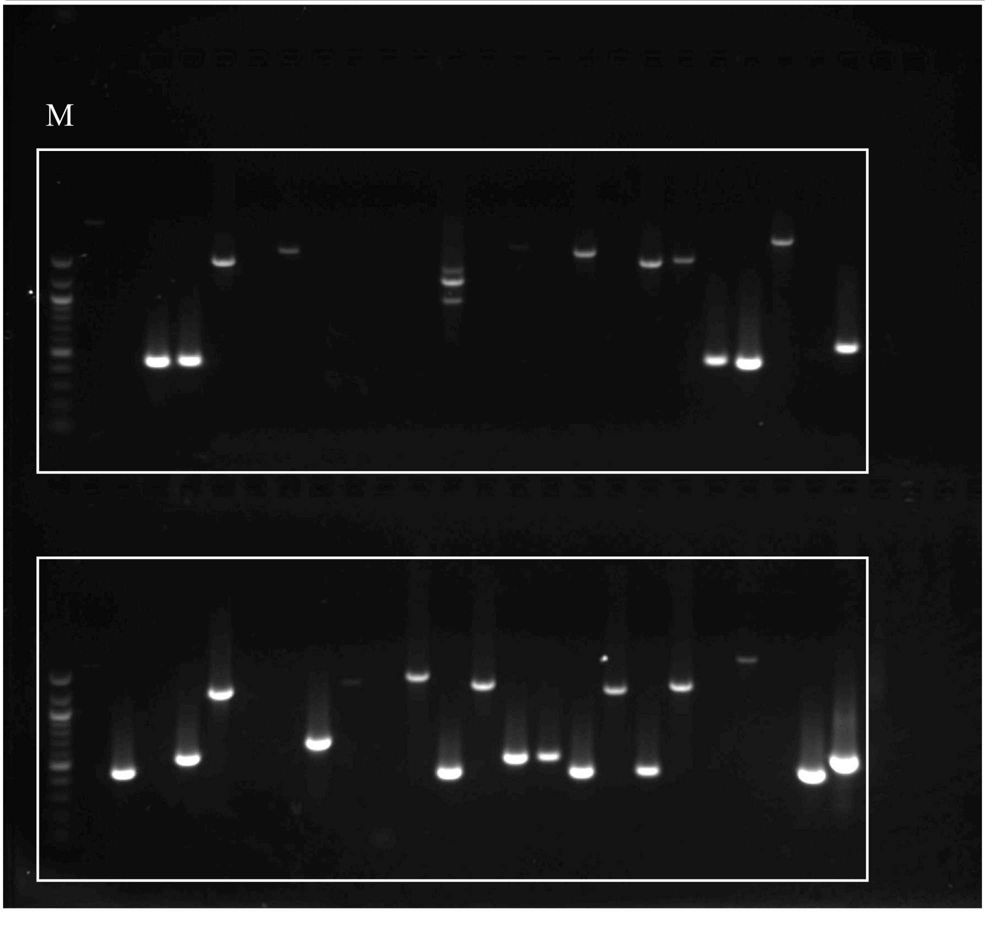

Raw images for Fig 6A . The images of western blots were taken using an Alpha Innotech FluorChem Q system. Individual panels were cropped from the raw images as defined by the white squares. The cropped panels were then inverted and adjusted for contrast using Photoshop; contrast adjustment was applied evenly across the entire image. The adjusted panels were assembled into Fig 6A using Adobe Illustrator. M: Protein marker.

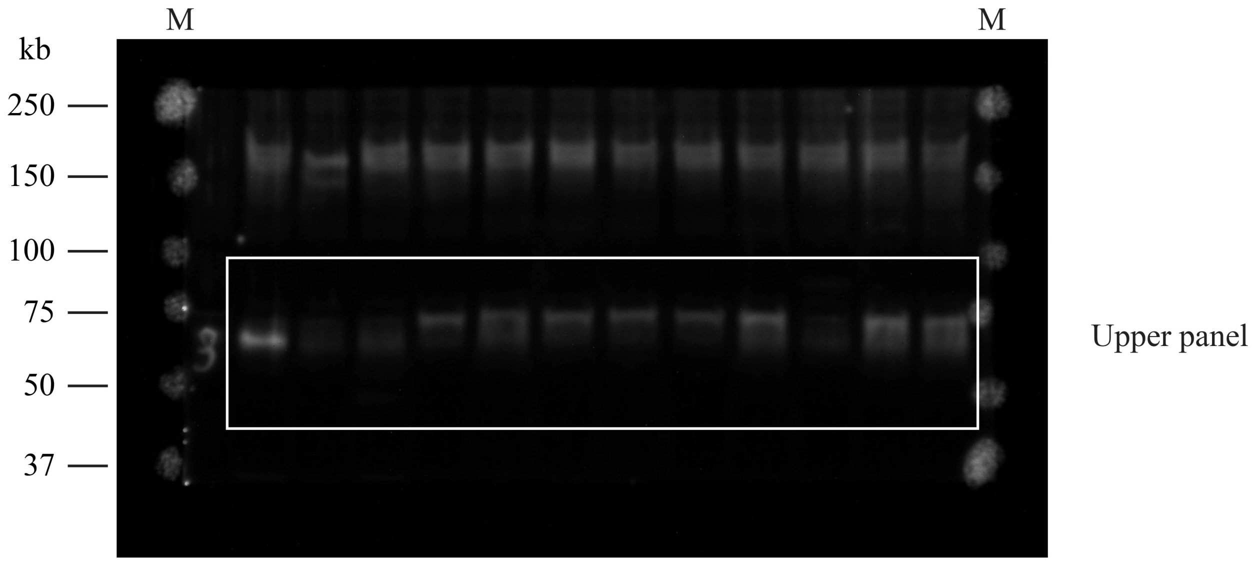

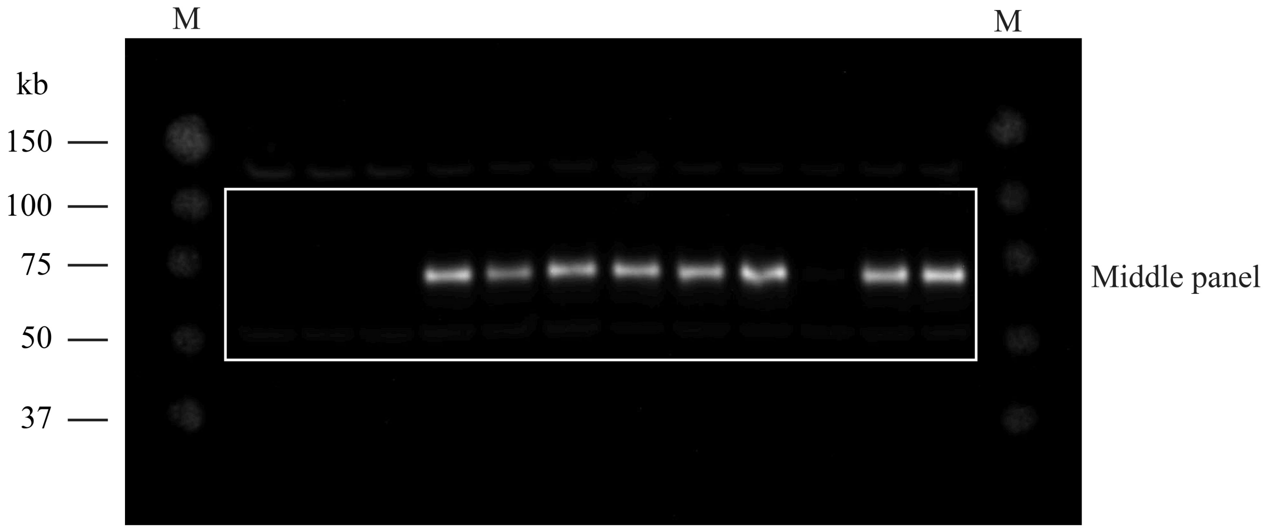

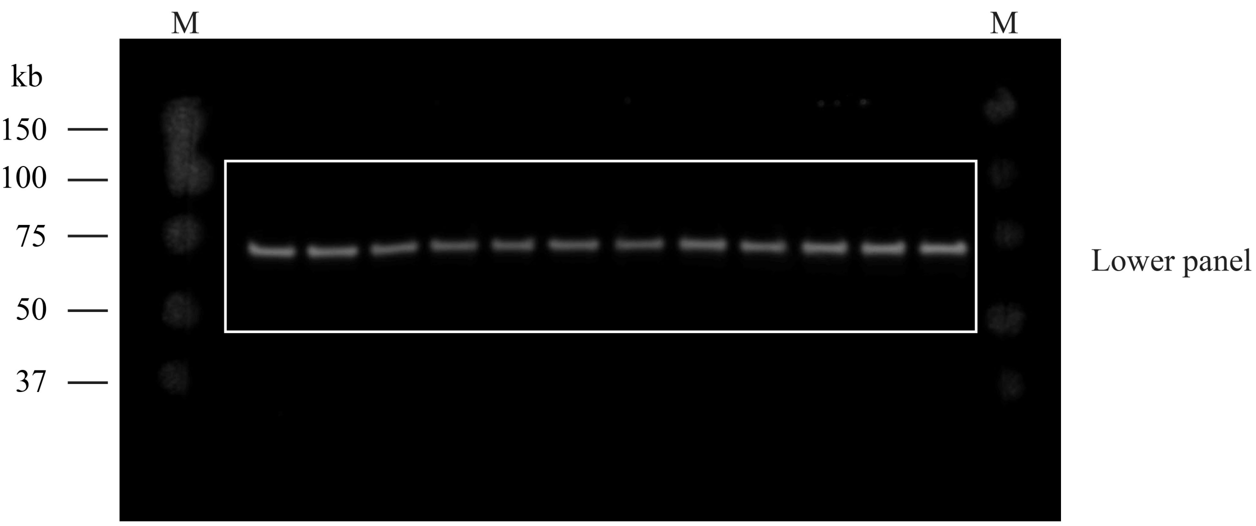

Raw images for Fig 7A . The images of DNA agarose gels were taken using an Alpha Innotech FluorChem Q system. Individual panels were cropped from the raw images as defined by the white squares. The cropped panels were then inverted and adjusted for contrast using Photoshop; contrast adjustment was applied evenly across the entire image. The adjusted panels were assembled into Fig 7A using Adobe Illustrator. M: DNA marker.

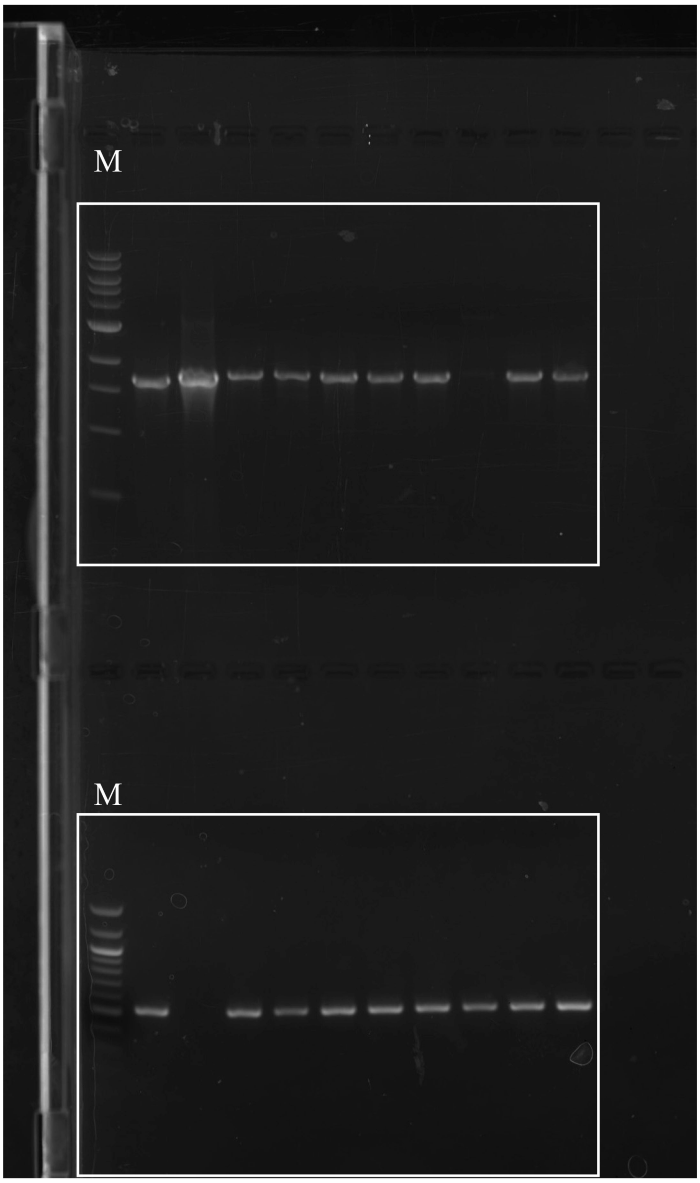

Upper panel

Lower panel

Raw images for Fig 8 . The images of DNA agarose gels were taken using an Alpha Innotech FluorChem Q system. Individual panels were cropped from the raw images as defined by the white squares. The cropped panels were then inverted and adjusted for contrast using Photoshop; contrast adjustment was applied evenly across the entire image. The adjusted panels were assembled into Fig 8 using Adobe Illustrator. M: DNA marker.

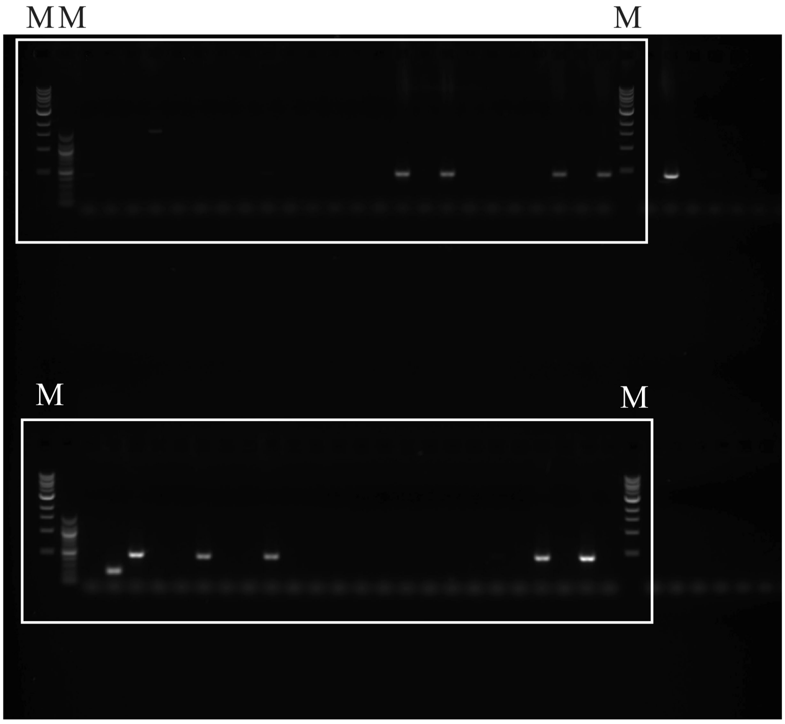

Top panel

Second panel

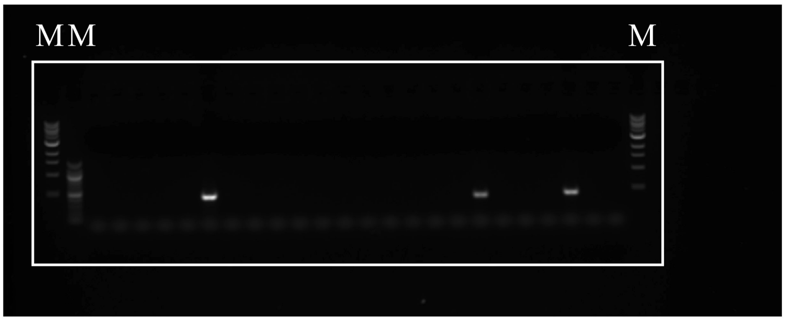

Third panel

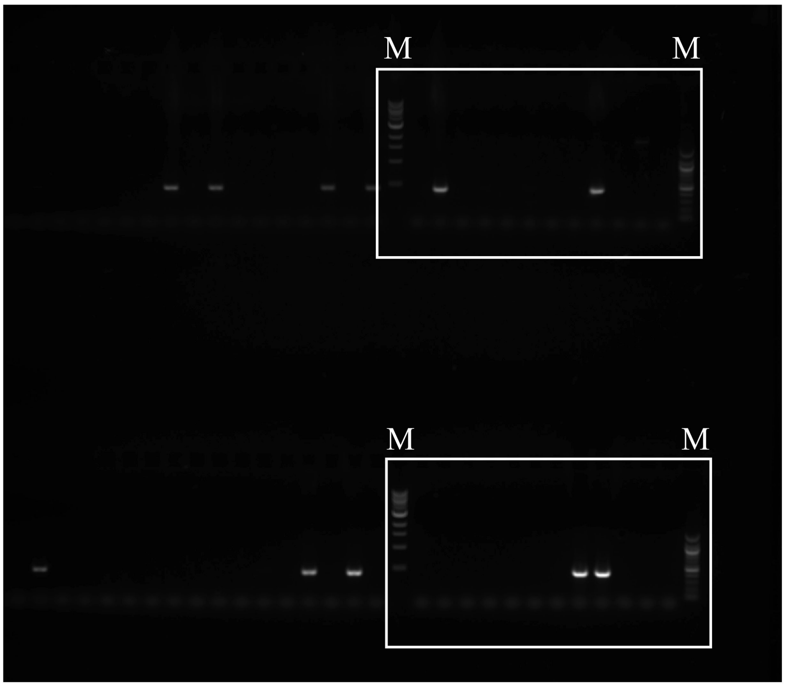

Left side of the fourth panel

Right side of the fourth panel

Raw images for Fig 9 . The images of DNA agarose gels were taken using an Alpha Innotech FluorChem Q system. Individual panels were cropped from the raw images as defined by the white squares. The cropped panels were then inverted and adjusted for contrast using Photoshop; contrast adjustment was applied evenly across the entire image. The adjusted panels were assembled into Fig 9 using Adobe Illustrator. M: DNA marker.

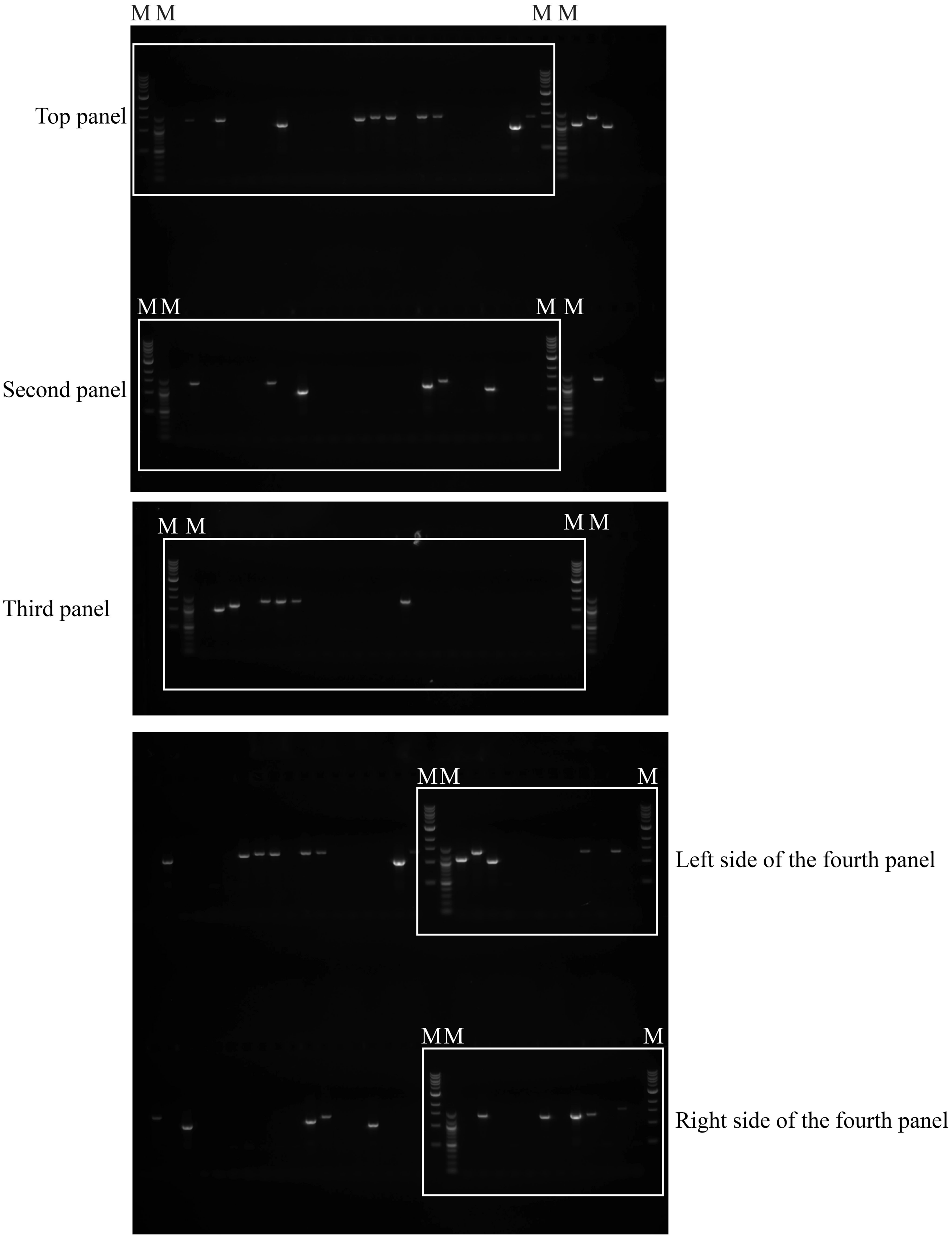

Raw images for Fig 11 . The images of western blots were taken using an Alpha Innotech FluorChem Q system. Individual panels were cropped from the raw images as defined by the white squares. The cropped panels were then inverted and adjusted for contrast using Photoshop; contrast adjustment was applied evenly across the entire image. The adjusted panels were assembled into Fig 11 using Adobe Illustrator. M: Protein marker.

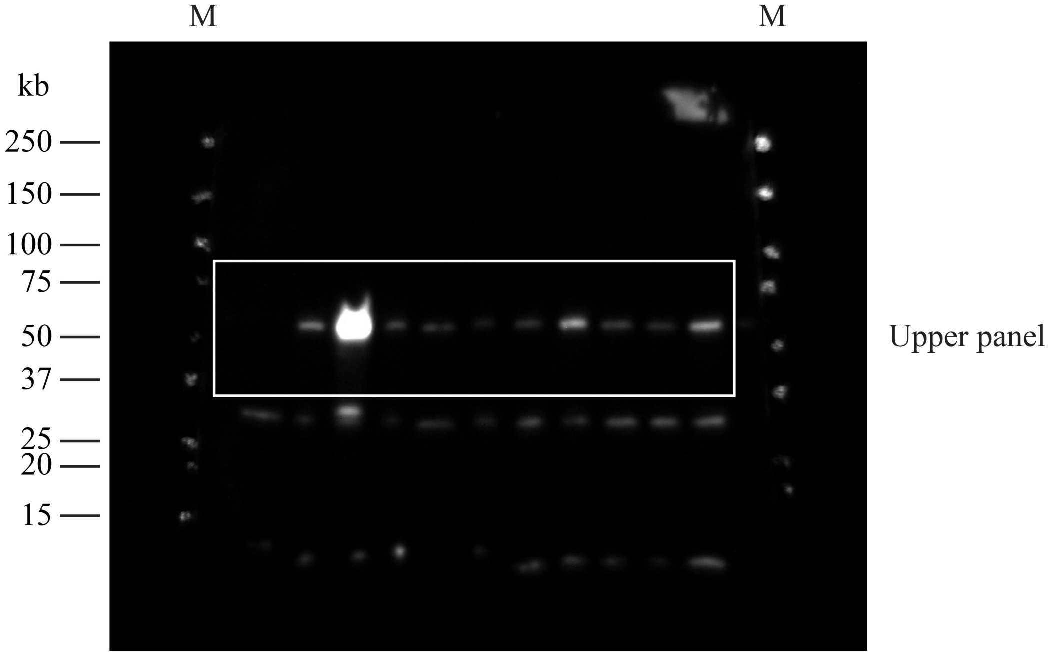

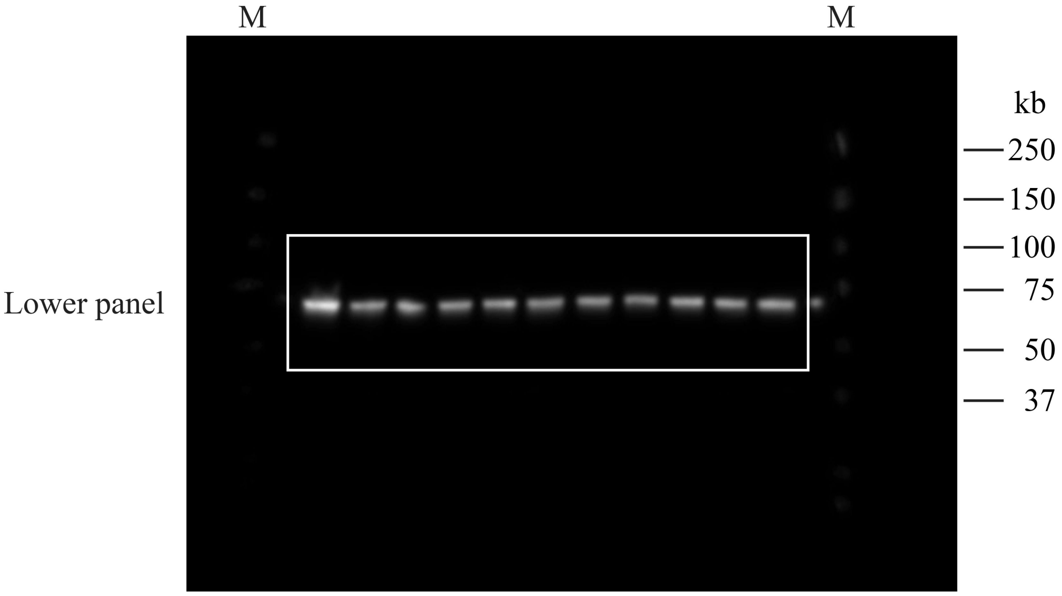

Raw image for S2 Fig. The image of a DNA agarose gel was taken using an Alpha Innotech FluorChem Q system. Individual panels were cropped from the raw image as defined by the white squares. The cropped panels were then adjusted for contrast using Photoshop; contrast adjustment was applied evenly across the entire image. The adjusted panels were assembled into S2 Fig using Adobe Illustrator. M: DNA marker.

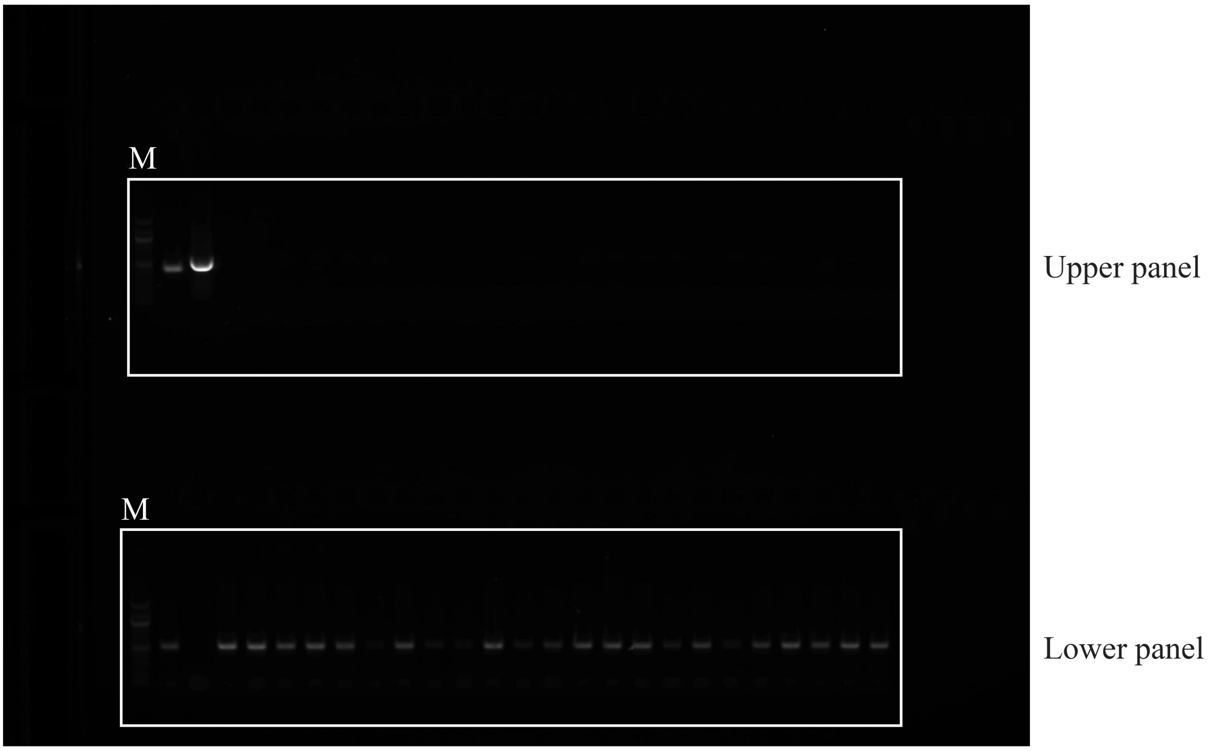

Raw image for S3 Fig. The image of a DNA agarose gel was taken using an Alpha Innotech FluorChem Q system. One panel was cropped from the raw image as defined by the white squares. The cropped panel was then adjusted for contrast using Photoshop; contrast adjustment was applied evenly across the entire image. The panel was then labelled using Adobe Illustrator to make S3 Fig. M: DNA marker.

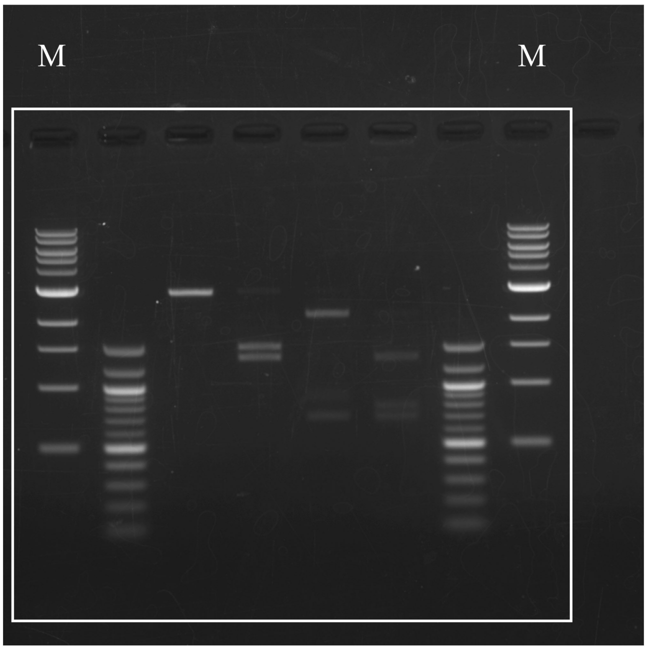

Raw image for S2 Appendix. The image of a DNA agarose gel was taken using an Alpha Innotech FluorChem Q system. One panel was cropped from the raw image as defined by the white squares. The cropped panel was then adjusted for contrast using Photoshop; contrast adjustment was applied evenly across the entire image. The panel was then labelled using Adobe Illustrator to make S2 Appendix. M: DNA marker.

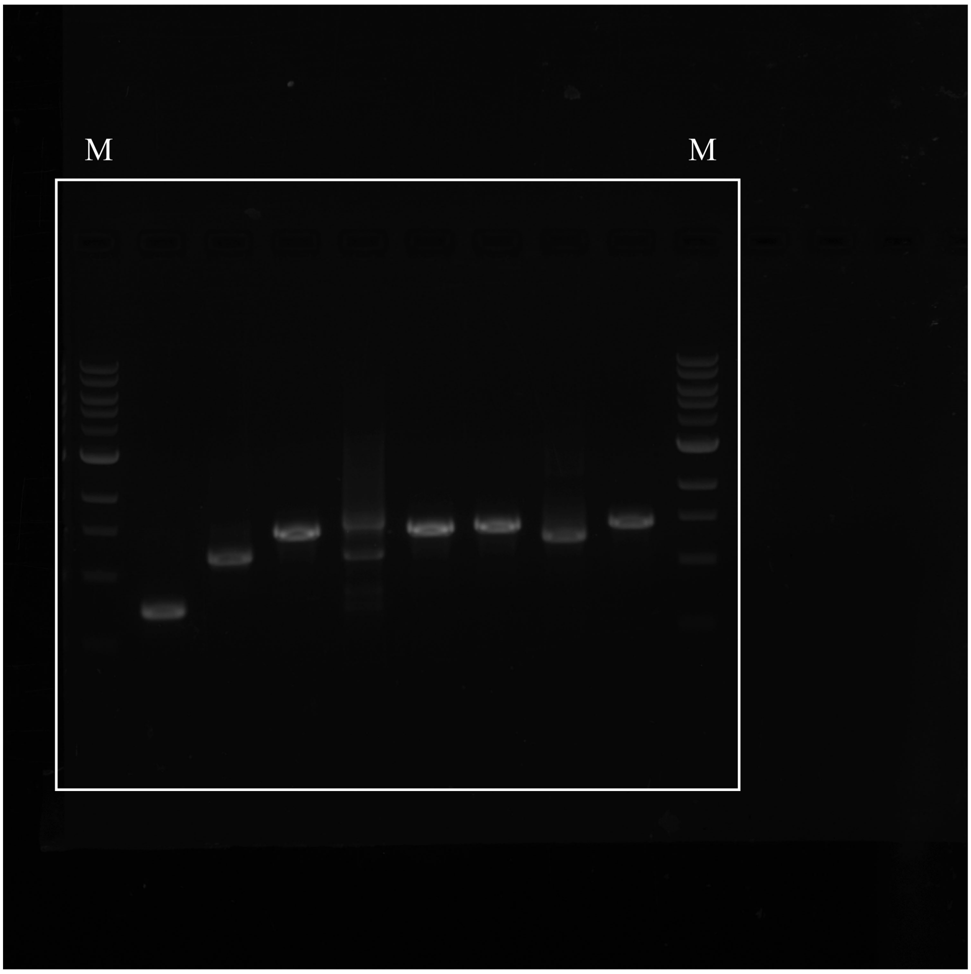

Supplement: S1 Raw images — (PDF) [file pone.0278972.s010.pdf]
