# Supplementary material for: Direct in situ protein tagging in Chlamydomonas reinhardtii utilizing TIM, a method for CRISPR/Cas9-based targeted insertional mutagenesis
Source: PLoS One. 2022 Dec 9;17(12):e0278972. doi: 10.1371/journal.pone.0278972 (PMC9733891; doi:10.1371/journal.pone.0278972)
Supplement: Attachment — Submitted filename: REV_PONE-D-22-20272_JRKim v1.docx [file pone.0278972.s011.docx]

**[Review comment]**

**PONE-D-22-20272**

**Decision : Minor Revision**

This paper introduces the most advanced technology that can be used for functional gene research using genome editing in microalgae. The tagging method based on the homologous recombination along with the TIM has a great advantage in that it can tag while maintaining the sequence of the endogenous gene and its expression. Unfortunately, these innovations do not provide new insights because they have already been applied to other species. However, if it is considered restricted to microalgae, it can be evaluated that it could contribute to technological progress.

It is required that more detailed information be supplemented in order to share more accurate technological innovation with readers through this paper.

Minor Review

1. Figure 1 should be re-draw in order to TIM and TIM-tagging methods can be understood conceptually. It should be different from ref [20].

2. Since this research is intended as an introduction to a novel gene editing strategy, a more detailed explanation is needed to help readers understand the TIM-tagging method. Add the structural description of TIM-tagging in the introduction or discussion.

3. Add the fluorescence image of mNeongreen in fig 11 or thereafter.
