# Supplementary material for: Direct in situ protein tagging in Chlamydomonas reinhardtii utilizing TIM, a method for CRISPR/Cas9-based targeted insertional mutagenesis
Source: PLoS One. 2022 Dec 9;17(12):e0278972. doi: 10.1371/journal.pone.0278972 (PMC9733891; doi:10.1371/journal.pone.0278972)
Supplement: Attachment — Submitted filename: Plos One _Witman_Review.docx [file pone.0278972.s012.docx]

The manuscript titled, ‘Direct in situ protein tagging in Chlamydomonas reinhardtii utilizing TIM, a method for CRISPR/Cas9-based targeted insertional mutagenesis’, by Hou et al has ued the green chlorophyte Chlamydomonas reinhardtii, a rather popular model system for flagellar studies to tag endogenous genes in situ using the modified TIM strategy. Undoubtedly, they have shown that this technique does circumvent the disadvantages of conventional approaches, the latter being more random. The authors have used this strategy on two genes: LF5/CDKL5, and Cre09.g416350/NAP1L1, both have not been studied previously in C. reinhardtii. The correct editing of these genes exhibit the success of the strategy (albeit at lower efficiencies).

I have only the following comments to make:

How equally useful is this strategy for other nuclear genes in *C. reinhardtii*?

What about the sfGFP in the final construct (lines 122-124)? Is it cleaved off?

Although the writing is simple and lucid, the authors have used three strains and must make mention of the type of strain they use in the M/M or wherever possible. It can get confusing, at times.

The authors have defended their modified TIM-based strategy to be relatively simple and quick, as opposed to the conventional approaches. A comparison in terms of time and ‘simplicity’ (whatever that implies) is required – especially since they claim this to be done for the first time in Chlamydomonas.
